# RAS signaling in lung adenocarcinoma is defined by lineage context and *DUSP4* loss

**DOI:** 10.1172/jci.insight.200912

**Published:** 2026-03-12

**Authors:** Minjeong Kim, Wisut Lamlertthon, Heejoon Jo, Yan Cui, Miyeon Yeon, Hyo Young Choi, Katherine A. Hoadley, Matthew P. Smeltzer, Michele C. Hayward, Matthew D. Wilkerson, Liza Makowski, D. Neil Hayes

**Affiliations:** 1 Department of Medicine, Division of Hematology and Oncology, College of Medicine, and; 2 UTHSC Center for Cancer Research, University of Tennessee Health Science Center, Memphis, Tennessee, USA.; 3 Princess Srisavangavadhana College of Medicine, Chulabhorn Royal Academy, Lak Si, Bangkok, Thailand.; 4 Department of Genetics, Genomics, and Informatics, and; 5 Department of Preventive Medicine, University of Tennessee Health Science Center, Memphis, Tennessee, USA.; 6 Department of Genetics, Lineberger Comprehensive Cancer Center, University of North Carolina at Chapel Hill, Chapel Hill, North Carolina, USA.; 7 Division of Epidemiology, Biostatistics, and Environmental Health, School of Public Health, University of Memphis, Memphis, Tennessee, USA.; 8 Lineberger Comprehensive Cancer Center, University of North Carolina at Chapel Hill, Chapel Hill, North Carolina, USA.; 9 Department of Anatomy, Physiology, and Genetics, Center for Military Precision Health, Uniformed Services University, Bethesda, Maryland, USA

**Keywords:** Cell biology, Clinical Research, Oncology, Biomarkers, Genetic variation, Lung cancer

## Abstract

**BACKGROUND:**

The molecular landscape of lung adenocarcinoma (LUAD) is often illustrated as a driver-oncogene pie chart, but identical mutations exhibit heterogeneous signaling shaped by comutations, transcriptional programs, and lineage context. We propose a lineage-integrated signaling framework using an EGFR mutation signature (mSig).

**METHODS:**

We defined EGFR mSig using differentially expressed genes in *EGFR*-mutant (*EGFR*-mt) LUADs. Semisupervised clustering and machine learning models were used to test reproducibility in different combinations of datasets. We analyzed molecular subtypes, lineage markers, co-occurring mutations, and *EGFR* copy number alterations in EGFR mSig-defined subtypes of LUAD.

**RESULTS:**

EGFR mSig showed robust classification performance (area under receiver operating characteristic curve = 0.83–0.95; mean negative predictive value = 96.3%). Validated gene expression subtypes and lung lineage markers were closely aligned with EGFR mSig status. Most EGFR mSig^+^ tumors, including many without *EGFR* mutations, belonged to the bronchioid subtype. A subset of canonical RAS mutations were mSig^+^ and mirrored the *EGFR* mutation pattern. *EGFR* WT/mSig^–^ tumors were enriched for nonbronchioid subtypes and had comutations in *TP53* or RAS/RAF/RTKs. We highlight a parsimonious collection of coordinated mutations, including RAS, *KEAP1*, *STK11*, *TP53*, and *CDKN2A*, that taken together suggest coordination of tumor signaling previously suggested but now reproduced and expanded.

**CONCLUSION:**

A potentially novel EGFR mSig that captures the transcriptional footprint of *EGFR* activation revealed a subset of *EGFR* WT LUADs with mt-like features. mSig refines LUAD taxonomy beyond mutation-only pie-chart models by incorporating lineage and comutation context. Lineage-directed stratification with coalteration identifies clinically relevant groups across *EGFR* and RAS states and highlights treatment opportunities for patients currently considered oncogene-negative.

**FUNDING:**

National Cancer Institute (NCI) U01CA272541, R01CA262296, U24CA264021, UG1CA233333, R01CA211939.

## Abstract 2

In lung cancer, a tumor’s “cell identity” shapes how key genes behave—revealing hidden drug targets even in patients previously labeled as having no actionable mutations.

## Introduction

More than 20 years ago, multiple investigators first reported frequent mutations in the epidermal growth factor receptor gene, *EGFR*, in association with therapeutic responses to selective kinase inhibitors (1). Progress in targeting *EGFR* inspired exhaustive efforts to identify additional oncogene targets with similar druggable potential, with numerous successes, including activating mutations of *BRAF*, *MET*, *FGFR*, *RET* and fusions of the genes *ROS1* and *ALK* as well as others (2). The precise fraction of lung adenocarcinoma (LUAD) with therapeutic targets remains an area of active research, as does the set of variants that are responsive to pharmacologic agents (3, 4). Despite sustained and intensive efforts to identified new druggable targets, many patients remain in the unsatisfying category of oncogene-negative (5, 6). Likewise, the therapeutic potential of some variants remains to be fully realized. For example, pharmacologic targeting of the most common driver, Kirsten rat sarcoma virus oncogene homologue, *KRAS*, has recently achieved notable success, representing a major step forward while continued efforts aim to expand efficacy (7–9). Failure to target oncogenic drivers by direct inhibition has led investigators to consider indirect strategies, including blockade of pathway-related signaling components. Downstream pathway blockade has been successful on occasion, such as MEK inhibition in some *BRAF*-mutant (*BRAF*-mt) tumors (10). However, in most cases, attempts to inhibit downstream signaling elements of activated oncogenes have yielded limited clinical benefit.

Partial explanations for the failure of downstream blockade have emerged in selected cases. For example, *BRAF*-mt colon cancers are refractory to BRAF inhibitors owing to compensatory upregulation of alternative pathway components (11). In contrast, for *KRAS*-mt LUAD, the mechanisms explaining failure of downstream inhibition appear to include heterogeneity in baseline signaling, even in the absence of pharmacologic inhibition (12). Identical *KRAS* sequence variants have alternative downstream signaling targets as a function of comutated genetic elements as well as the molecular context of expressed lineage transcription factors (13, 14). At least 3 well-established signaling subtypes of KRAS have been suggested as a function of the lineage marker *NKX2-1* (encoding the protein TTF1); mutations in *TP53*, *STK11*, and *KEAP1*; and other factors (15–19). While mostly untested, the hypothesis suggests that different groups might have different therapeutic options, including downstream blockade in cases where the correct KRAS class and downstream targets were characterized.

Early classification studies of LUAD identified 3 intrinsic molecular subtypes — bronchioid, magnoid, and squamoid — reflecting major axes of lineage-associated differentiation and tumor biology (20, 21). These bronchioid/magnoid/squamoid molecular signatures were subsequently validated in The Cancer Genome Atlas (TCGA) LUAD report, which adopted updated terminology, including terminal respiratory unit (TRU)/proximal-proliferative (PP)/proximal-inflammatory (PI), respectively (22). Regardless of nomenclature, bronchioid/TRU tumors typically retain *NKX2-1* expression and exhibit lepidic or papillary architecture (22–24). The magnoid/PP class, also referred to as “19p-depleted,” is characterized by frequent KEAP1 and STK11 (both genes located on chromosome 19p) mutations and deletions. In contrast, squamoid/PI tumors demonstrate inflammatory transcriptional programs and are commonly associated with solid morphologic features (22, 25, 26). Because these lineage-defined classes influence MAPK signaling output and therapeutic vulnerability independent of mutation status, we retain the bronchioid/magnoid/squamoid terminology for continuity with the original intrinsic classification, although the TRU/PP/PI terminology is equivalent.

Recognizing that recently identified therapeutic targets increasingly represent small, clinically narrow patient subsets within LUAD (27) and that the incremental proportion of patients who benefit from each newly discovered target appears increasingly modest at the population level, there remains a critical need for complementary strategies that capture shared lineage and transcriptional biology beyond mutation status alone. Given the data suggesting that multiple RAS pathway elements in LUAD appear to have heterogenous signaling alternative pathways (BRAF and KRAS), we hypothesized that the gene expression profiles of patients with mutated *EGFR* might provide insights into the signal status of other known and unknown downstream elements of RAS signaling. Based on the *BRAF* and *KRAS* experience, we also considered that the molecular context, such as previously validated gene expression subtypes (20–22, 26), comutated oncogenes (such as *TP53*), and transcription factors, might influence signaling networks and therapeutic targets and even unmask aspects of tumorigenesis.

## Results

### Patient characteristics.

A total of 792 patients with gene expression, mutation, and clinical data were available for analysis and divided into a training cohort (*n* = 192, Memorial-Sloan Kettering Cancer Center [MSKCC]) and 2 validation cohorts (validation cohort 1: *n* = 114, combined University of North Carolina at Chapel Hill [UNC] and Tumour Sequencing Project [TSP]; validation cohort 2: *n* = 486, TCGA; Table 1). Patients were assigned to gene expression subtypes using methods that have been previously reported (20, 21). Overall, the cohorts were similar in their composition with minor exceptions.

### EGFR mutation signature predicts EGFR-mutant-like LUAD tumors.

**Figure 1:**
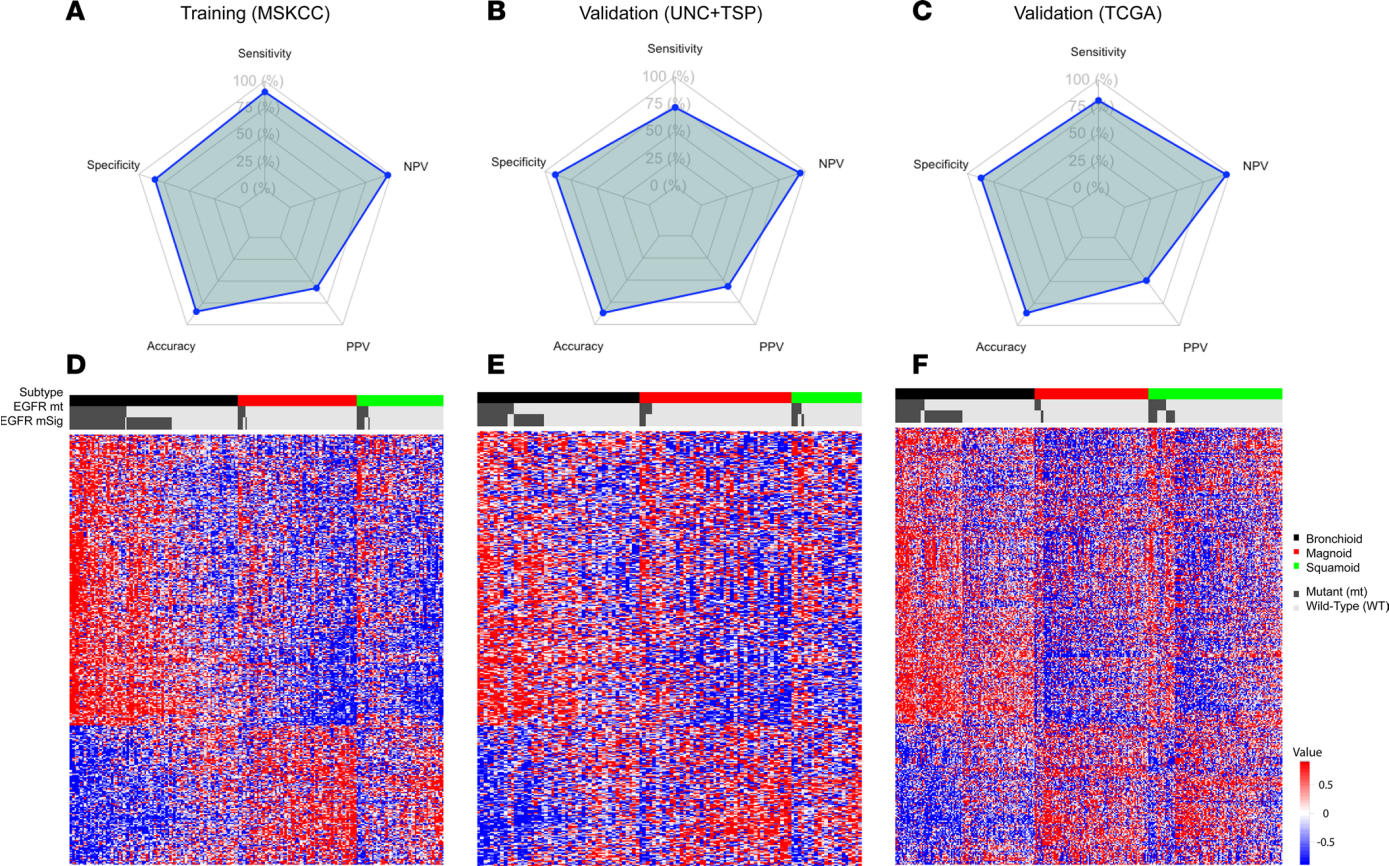
Performance and reproducibility of the EGFR mutation signature in LUAD. (**A**–**C**) Radar plots showing performance metrics of the EGFR mutation signature (mSig) across datasets. (**D**–**F**) Heatmaps of gene expression profiles of patients with LUAD semisupervised by EGFR mutation status and molecular subtype. Rows represent the 1,020 genes included in the EGFR mSig. Columns represent individual patients, clustered by LUAD molecular subtypes (bronchioid, magnoid, and squamoid), with *EGFR* mutation and mSig status using a semisupervised approach. Annotations indicate EGFR mutation status and EGFR mSig status. mt or mSig^+^ tumors are shown with dark gray bars, and WT tumors are shown with light gray bars. (**A** and **D**) Training cohort (MSKCC). (**B** and **E**) Validation cohort 1 (UNC + TSP). (**C** and **F**) Validation cohort 2 (TCGA). NPV, negative predictive value; PPV, positive predictive value; mt, mutant.

For the purpose of defining expression patterns associated with *EGFR* mutation activation, we constructed a series of supervised models predicting *EGFR* mutation status under a wide variety of conditions, all of which demonstrated highly similar performance (Figure 1 and Supplemental Figure 1; supplemental material available online with this article; https://doi.org/10.1172/jci.insight.200912DS1). From the training data set (MSKCC), a model with 1,020 differentially expressed genes was selected using the classification to the nearest centroids (ClaNC) technique (Supplemental Table 1 and Supplemental Figure 2). A total of 690 genes were overexpressed and 330 genes were underexpressed in *EGFR*-mutant (*EGFR*-mt) tumors compared with *EGFR* WT tumors. Varying the number of genes included in the model from a few dozen to several thousand had little effect on the effect on the ClaNC training performance. The choice of 1,020 genes was empiric and intended to be of sufficient size to allow other analyses to be performed, such as gene set enrichment–type studies.

In training, sensitivity (90%), specificity (84%), and negative predictive value (NPV, 97%) were high, while positive predictive value (PPV) was modest at 58%. Validation experiments showed little decline in performance (Figure 1, A–C, and Supplemental Table 2). Alternative supervised models, used to demonstrate robustness and generalizability of the EGFR mutation signature (mSig), showed similar training and testing properties, and, thus, alternative models will not be presented in further detail (Supplemental Figure 1). To understand the contributions of each gene toward the supervised analysis, we also generated a ranked order of genes using the SamR algorithm (Supplemental Table 1). As expected, *EGFR* gene expression ranked high among the most differential genes associated with mutation status. Pathway analysis of the over- and underexpressed genes documented signatures that have been previously reported in association with *EGFR* mutation such as upregulation of kinase activity and downregulation of apoptotic processes (Supplemental Figure 3) (21). Thus, we concluded that by using gene expression and across a wide variety of model types and conditions, it was possible to obtain high specificity and NPV for *EGFR*. However, sensitivity was modest, averaging 81%, and PPV was generally suboptimal at average 55%. In other words, it was possible to predict samples that lacked the EGFR mSig, although many cases with an identical gene expression pattern lacked the activating mutation. We interpreted this to mean that some samples lacking the *EGFR* mutation nonetheless demonstrated gene expression signaling overlapping with samples that contain the *EGFR* mutation. All cases predicted to have the signature of *EGFR* mutation were labeled as mSig^+^, whether the mutation was observed or not.

We then considered the properties of mSig^+^ samples lacking the *EGFR* mutation. Noting that prior studies have validated a statistically significant association between activating *EGFR* mutations and the bronchioid subtype (21), we considered the association between false positive calls for *EGFR* mutation and the molecular subtypes of LUAD. In each of the training and validation datasets, several reproducible patterns were observed when the supervised predictor genes for *EGFR* mutation status were considered (Figure 1, D–F). First, the EGFR mSig developed for the supervised analysis of *EGFR*-mt tumors versus *EGFR* WT tumors also clearly distinguished the bronchioid subtype from the magnoid and squamoid subtypes, with most mSig^+^ cases colabeled as bronchioid. Likewise, most of the *EGFR*-mt cases and nearly all of the false positive mSig^+^ patients (*EGFR* WT/mSig^+^) were associated with the bronchioid gene expression subtype. Conversely, most of the *EGFR*-mt cases predicted as mSig^–^ (false negative) were nonbronchioid samples. In other words, the supervised mSig^+^ prediction appeared to largely represent a subset of the unsupervised bronchioid molecular subtype. Putting this finding into a physiologic and histologic context, mSig^+^ patients were more likely to be represented by more differentiated histologic subtypes of LUAD (Supplemental Table 3) (23).

**Figure 2:**
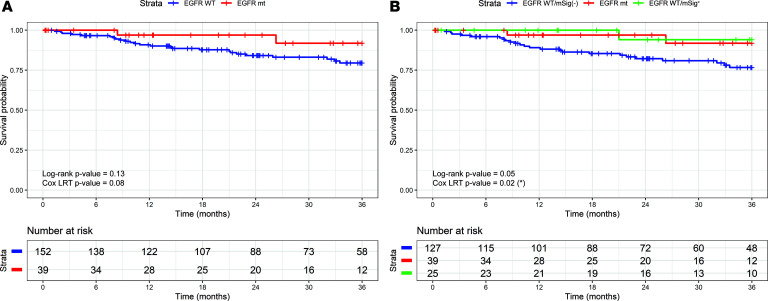
Three-year overall survival analysis based on EGFR-related signature groups in LUAD cohorts. Three-year Kaplan-Meier survival curves comparing (**A**) 2 groups (*EGFR* mt, red, and *EGFR* WT, blue) and (**B**) 3 groups (*EGFR* mt, red; *EGFR* WT/mSig^–^, blue; *EGFR* WT/mSig^+^, green). *P* values were calculated using the log-rank test and the Cox proportional hazards model (likelihood ratio test [LRT]).

We next considered whether *EGFR* WT/mSig^+^ cases share other properties with *EGFR*-mt cases, including clinical outcomes. First, we confirmed that *EGFR*-mt patients have more favorable 3-year overall survival outcomes than *EGFR* WT patients (log-rank, *P* = 0.13; Figure 2A) (28). We then stratified the outcomes of those patients with EGFR mSig^+^ as a function of EGFR mutation status and found similarly favorable outcomes for both *EGFR*-mt and EGFR WT/mSig^+^ when compared with EGFR WT/mSig^–^ patients (log-rank, *P* = 0.05; Cox likelihood ratio test, *P* = 0.02) (Figure 2B). In many cohorts, including the MSKCC, UNC and TCGA cohorts, it has been previously reported that the bronchioid subtype also has a similar favorable outcome compared with that of the magnoid and squamoid subtypes (20). In short, bronchioid subtype, *EGFR*-mt, and *EGFR* WT/mSig^+^ belong to a group with shared gene expression and differential patient outcome.

### EGFR mSig correlates with LUAD lineage markers, unsupervised expression subtypes, and driver genomic alteration events in LUAD.

**Figure 3:**
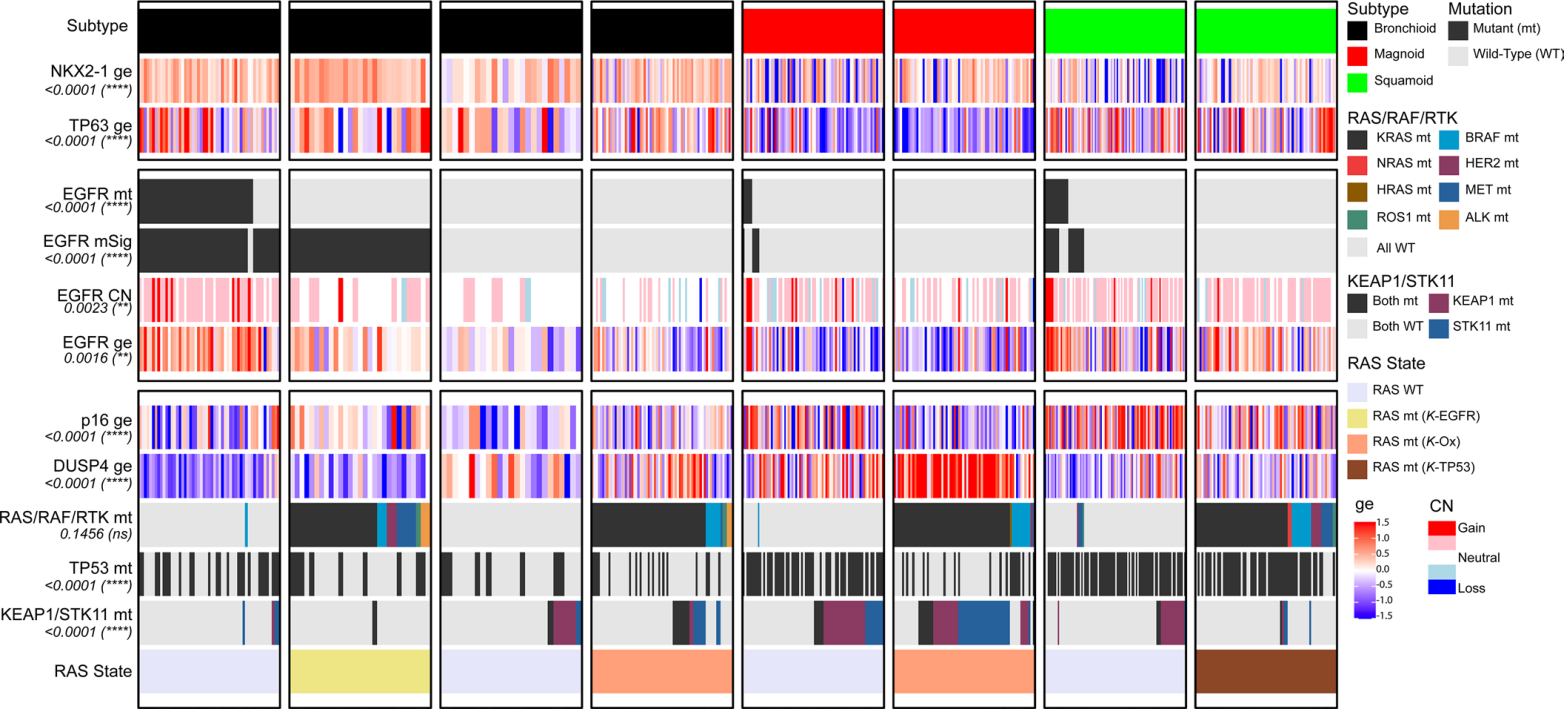
Integrative analysis of genomic alterations and gene expression across molecular subtypes of LUAD. Samples (*n* = 486, TCGA LUAD) are represented in columns and grouped by molecular subtype. Within each subtype, tumors were further stratified according to EGFR mutation status and mSig classification. White space separators indicate the presence or absence of mutations within the RAS/RAF/RTK signaling pathway. RAS mutations include 3 different states: *EGFR*-like *KRAS* (*K*-EGFR), oxidative stress–related *KRAS* (*K*-Ox), and *TP53*-associated *KRAS* (*K*-TP53). Gene expression differences were assessed using 1-way ANOVA, and categorical genomic alterations were evaluated using the χ^2^ test. Statistical significance reflects subtype-specific differences in the genomic features (**P < 0.01; ****P < 0.0001). ge, gene expression; mt, mutant; CN, copy number.

We next considered whether the shared EGFR pathway, expression signature, and clinical outcomes might correlate with other molecular markers, such as lineage-directing transcription factors or other driver gene mutations. To further define true and false positive mSig^+^ patients, we examined the set of commonly altered driver LUAD genes and selected pulmonary lineage markers, the most highly associated with the canonical markers *NKX2-1* and *TP63* (29, 30). Lung lineage genes were statistically associated with expression of the bronchioid subtype in our study as well (Figure 3). The squamoid group was highly associated with *TP63* and with overall lower expression of *NKX2-1*, while the magnoid group had relatively low expression for both markers. Such a pattern demonstrates that unsupervised molecular subtypes, supervised mutation expression signatures, and *EGFR* mutation itself correlate with expression of critical lung lineage markers. While we highlighted only 2 transcription factors commonly used in clinical classification for more than 20 years, a more nuanced set of pulmonary-associated transcription factors, such as *SOX2*, *FOXA1*/*2,* and others added further support to the concept that gene expression subtypes and their associated oncogenes reflect underlying states of cell of origin and stage of differentiation features of LUAD (Supplemental Figure 4) (31–34).

We further investigated the correlation of driver gene alterations and mSig^+^ cases as a function of unsupervised expression subtypes. Seventy-five percent of mSig^+^ patients (*n* = 80/106) were of bronchioid subtype (bronchioid vs. EGFR mSig: OR = 9.2, *P* < 0.001); the remaining 25% were mostly of the squamoid subtype, and there were very few of the magnoid subtype (Supplemental Figure 5A and Supplemental Table 4). Inspection of *EGFR* gene expression followed a similar pattern, with most of the highly expressed cases in the bronchioid subgroup found in association with *EGFR*-mt, *EGFR* amplification, or both in association with mSig^+^ status (Supplemental Figure 5, A and B, and Supplemental Table 5). A significantly lower or absent *EGFR* expression and absent amplification was observed across the magnoid group in association with *EGFR* WT and mSig^–^ status (Supplemental Figure 5C). The few *EGFR*-mt magnoid cases that were observed did not generally present with an associated mSig^+^ status, a pattern also observed to a lesser degree in squamoid samples (Supplemental Figure 5D). Interestingly, approximately half of the mSig^+^ bronchioid patients expressed *EGFR* at a much more modest level and were *EGFR* WT, and nearly all of those patients had a documented alternative canonical RAS-activating mutation had a documented alternative canonical RAS-activating mutation. In contrast, although most squamoid samples expressed *EGFR* at higher levels, the mSig^+^ signature was generally absent, and although RAS mutations were common, almost none demonstrated the mSig^+^ pattern.

The bronchioid subtype was remarkable in that 75% of cases had either a canonical *EGFR* mutation or RAS mutation, with 50% of the tumors with RAS mutations demonstrating the mSig^+^ signature and 50% showing an alternative signaling pathway. The observation that RAS mutations assume more than one signaling configuration has been previously reported by Skoulidis and others (13, 35), although to our knowledge the emphasis was on differential signaling in association with *TP53* mutation, *CDKN2A* loss, *STK11* loss, and low *NKX2-1* gene expression and not the RAS-mt group described here, which is most remarkable for demonstrating the EGFR mSig^+^ signal in combination with high expression of *NKX2-1*, *TP53* WT and *STK11* WT. In support of the previously reported heterogenous KRAS signaling subtype characterization (13), we investigated the role of *CDKN2A* alteration, including inactivating mutations/losses (data not shown) and gene expression (Figure 3). We confirmed patterns of differential *CDKN2A* gene expression, including relatively low expression in the *EGFR*-mt samples, a finding that could be interpreted as the loss of senescence pathways in tumors with an oncogene addiction to activated *EGFR*. Likewise, RAS-mt tumors of the magnoid subgroup, but not other RAS-mt samples, demonstrated statistically significant decreased *CDKN2A* expression (RAS/RAF/RTK-mt vs. p16/CDKN2A ge: OR = 0.33, *P* < 0.001). Although beyond the scope of the current report, additional driver mutations were demonstrated in association with molecular subtypes and mSig status (Supplemental Table 6) (22, 36). To further clarify the classification of RAS mutation states as well as build on prior works (13, 35), we relied on the previously suggested class names of “*STK11*/*LKB1*-dependent” and “*TP53*-associated” and added our subtype of EGFR-pathway-like *KRAS* mutation. For the purposes of abbreviation, we used the terms oxidative stress-related *KRAS* (*K*-Ox), and *TP53*-associated *KRAS* (*K*-TP53), and *EGFR*-like *KRAS* (*K*-EGFR), respectively.

### DUSP4 and other potential therapeutic targets of EGFR WT LUAD.

Since most *EGFR* mutations are in the bronchioid subtype and most *EGFR* WTs are nonbronchioid, comparison of *EGFR*-mt and WT could be confounded by genes that define tumor molecular subtype. To the extent that the differences in molecular subtypes are driven by differences in driver genes, confounding may not be a problem. However, as Figure 3 demonstrates, other factors, such as lineage transcription factors, are a strong component of the tumor subtype expression signatures. For example, since bronchioid and magnoid differ dramatically by *NKX2-1* expression, *EGFR* expression, copy number, and mutation, genes associated with lineage and EGFR signaling will be highly confounded. Lineage confounding (and other potential unknown confounders) of *EGFR* mutation status might be partially overcome by stratifying differential gene expression between driver gene variants within a single molecular subtype. Although a number of supervised analysis strata could be entertained, we considered *EGFR* mutation status for mt versus WT within the bronchioid subtype and across all samples and all subtypes (Supplemental Figure 6A). Although the prediction direction and effects were highly correlated for many genes within the bronchioid subtype versus across all patients, candidates related to RAS signaling not previously appreciated rose in their discriminating power. Differentially expressed genes in bronchioid tumors recapitulated the distinct molecular profiles observed across all subtypes in multiple cohorts (Supplemental Figure 6, B and C). Most notably, the gene dual specificity phosphatase 4 (*DUSP4*) was identified as significantly upregulated in the *EGFR*-mt/mSig^+^ versus EGFR mSig^–^ within the bronchioid subtype via GSEA (normalized enrichment score = 1.3, nominal *P* < 0.01, data not shown) but not across all subtypes.

DUSP4 is a protein phosphatase that inactivates kinases by dephosphorylating both the phosphoserine/threonine and phosphotyrosine residues. It negatively regulates members of the MAP kinase superfamily (MAPK/ERK, SAPK/JNK) (37–39) and prevents oxidative stress–driven apoptosis by blocking the phosphorylation of p38 (40). We evaluated whether *DUSP4* is supported as a therapeutic target using model system data, including both pharmacologic and genetic perturbation approaches. Human cell line data were obtained from DepMap (41), focusing on LUAD models that recapitulated the mutation and gene expression patterns observed in the clinical cohort shown in Figure 3. We annotated those cell lines according to gene expression subtype, mSig status, and RAS and *EGFR* mutation status (*n* = 45; Supplemental Figure 7). In the investigation of CRISPR/Cas9 knockout screening-based gene dependency scores and the Sanger Genomics of Drug Sensitivity in Cancer (GDSC1) dataset (42, 43), two illustrative examples emerged: A549 (*EGFR* WT/*KRAS*-mt) and EKVX (*EGFR* WT/*KRAS* WT). Both were classified as magnoid subtype. As anticipated, the *KRAS*-mt A549 cell line demonstrated marked dependency on *DUSP4* knockout with higher sensitivity to cisplatin [dependency probability >0.2; ln(IC_50_) ~ 1], whereas the *KRAS* WT EKVX cell line was less dependent on *DUSP4* knockout and more resistant to cisplatin [dependency probability ~0; ln(IC_50_) > 5] (41–43). Taken together, the combination of prior functional studies and supporting analyses using cell line data suggests that differential *DUSP4* gene expression might correlate with differential activation of the MAPK pathway. Projecting *DUSP4* expression onto the integrated driver data revealed intriguing patterns both for the patients with *EGFR* WT magnoids as well as other subgroups of patients with LUAD. For all *EGFR*-mt and mSig^+^/RAS-mt tumors, *DUSP4* expression was uniformly low. In contrast, *DUSP4* demonstrated significantly elevated expression, especially in *EGFR* WT/*KRAS*-mt bronchioid and magnoid tumors but not *KRAS*-mt squamoid tumors.

### Coordination of lineage, oncogene signaling, proliferation, and metabolic/oxidative stress in LUAD.

**Figure 4:**
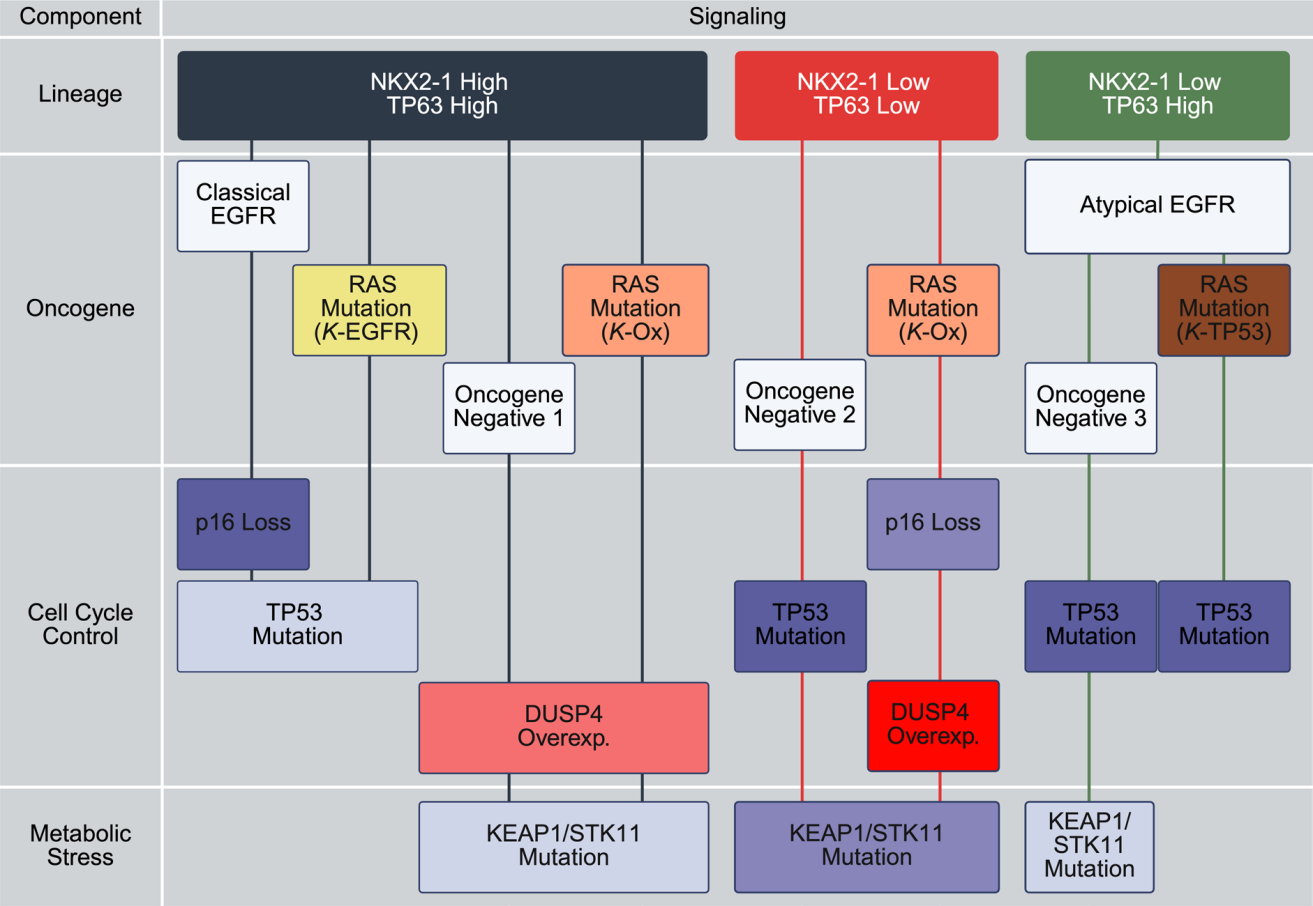
A proposed framework linking 4 biological components across lineage-defined molecular subtypes of LUAD. This schematic model illustrates the proposed subtypes of LUAD stratified by lineage features (*NKX2-1* and *TP63* expression) and integrates alterations across key oncogenic, cell cycle control, and metabolic stress components. Each component highlights distinct molecular features that collectively define the biological heterogeneity and potential therapeutic vulnerabilities of each subtype. p16 loss and the frequencies of *TP53* and *KEAP1*/*STK11* mutations are shown using a purple gradient. *DUSP4* expression levels are shown using a red gradient. This figure was created with https://app.biorender.com

We next sought to augment the recently reported RAS classification proposed by Skoulidis et al. with unsupervised gene expression subtypes that were first reported more than 20 years ago (13, 20). In this scheme, 3 unsupervised tumor classes (bronchioid, squamoid, magnoid) correlate highly with the lineage markers *NKX2-1* and *TP63*, suggesting an underlying cellular context of differentiation or cell of origin among tumors characterized as LUAD (Figure 4). Within each cellular context, tumors demonstrated differential patterns of alterations in common driver genes associated with oncogene activation (mutations in *EGFR* or RAS or oncogene-negative), altered proliferation (loss or mutation of tumor suppressor genes, including *DUSP4*, a major regulator of MAPK signaling pathway), and metabolic/oxidative stress (AMPK signaling failure due to *STK11* mutation and redox homeostasis failure due to KEAP1-NRF2 dysregulation) (18, 19). Oncogene activation was defined by either an mSig^+^ signature associated with *EGFR*-mt or previously defined RAS mutations (*K*-EGFR and *K*-Ox) (35). The remaining RAS-mt samples, entirely limited to squamoid subtype, demonstrated neither mSig signature nor high *DUSP4* expression, suggesting a RAS mutation (*K*-TP53) for its high proportion of *TP53* mutations. More than 90% of all squamoid tumors, including those in RAS-mt patients, had *TP53* mutations. In contrast, *EGFR*-mt/mSig^+^, mSig^+^/RAS-mt (*K*-EGFR), and mSig^–^/RAS-mt (*K*-Ox) tumors were infrequently *TP53*-mt squamoid. Interestingly, squamoid samples were overall lacking mSig^+^ cases, despite approximately 50% RAS mutation, frequent amplifications of EGFR, and higher EGFR gene expression. We empirically describe this group as demonstrating atypical *EGFR* expression based on lack of mSig and the coordinated expression of *EGFR* in the presence of RAS mutation.

Skoulidis et al. described not only the association of one RAS mutation with *TP53* mutation such as we see in squamoid expression class, but also an alternative means of affecting proliferation through altered *CDKN2A* (13). We also observed this to a limited degree with RAS-mt (*K*-Ox) cases but recorded more prominent losses of *CDKN2A* in *EGFR*-mt/mSig^+^ patients. Considering DUSP4, a repressor of RAS signaling as the third most prominent proliferation signa, demonstrates a high degree of coordination across the subtypes proposed in the model. Finally, as suggested by both Skoulidis and others (22, 44), altered metabolic/oxidative stress mutations co-occur in reproducible ways, especially in the magnoid group where nearly every RAS-mt case (*K*-Ox) has concomitant metabolic/oxidative stress mutations, whereas *K*-EGFR or *K*-TP53 cases have almost none. An alternative view of the data in which only *KRAS* mutations are shown does not appreciably change the interpretation (Supplemental Figure 8).

Considerable prior work has suggested that, beyond *EGFR* mutation status alone, alternative biomarkers, including gene expression signatures, can identify patients likely to respond to EGFR-directed therapies. In particular, an *EGFR* gene expression signature has been shown to correlate with sensitivity to the EGFR inhibitor gefitinib in a manner similar to *EGFR* mutation status (45). Such signatures may also identify tumors dependent on downstream EGFR signaling, including subsets of *KRAS*-mt cancers. The subclassification of *KRAS* mutations therefore opens new possibilities for EGFR pathway–directed therapies. We hypothesized that tumors classified as mSig^+^, including many *K*-EGFR *KRAS*-mt cases, might exhibit EGFR pathway dependency related to drug sensitivity signatures. To explore this, we characterized mSig^+^ tumors in the context of RAS mutation status and previously published gefitinib sensitivity scores (45), with particular attention to *KRAS* comutation patterns, long considered biomarkers of resistance to EGFR-directed therapy (Supplemental Figure 9). EGFR inhibitor responsiveness scores varied significantly as a function of combined *EGFR* and *KRAS* mutation status. *EGFR*-mt tumors and *K*-EGFR RAS-mt tumors (the majority of which were EGFR mSig^+^) demonstrated high predicted responsiveness scores. In contrast, *K*-Ox and *K*-TP53 tumors exhibited low EGFR responsiveness scores. Thus, EGFR-mt and *K*-EGFR tumors share common downstream signaling dependencies that may be therapeutically targetable, potentially through MEK or ERK inhibition (46–48). In contrast, *K*-Ox and *K*-TP53 tumors may require alternative treatment strategies. Prior efforts targeting downstream EGFR signaling in *EGFR*-mt disease may therefore provide a framework for therapeutic development in selected KRAS-mt subsets.

## Discussion

EGFR kinase mutations generate a reproducible gene expression signature, with consistent prediction accuracy across predictor type, model parameters, and expression assays. The prominence of a single EGFR mSig is unexpected since other LUAD oncogenes, including *BRAF* and *KRAS*, often display multiple, context-dependent signatures (49). For BRAF, it signals differently depending on tissue and anatomic location (e.g., colonic epithelium vs. melanocyte) (50). KRAS signaling is heterogeneous within a single tissue, shaped by comutations (*TP53*, *KEAP1*, *STK11*, etc.) and pulmonary lineage marker *NKX2-1* (15–17). Context is also crucial for *EGFR*. Although EGFR signaling is relevant in many tumor types, kinase mutations occur almost exclusively in LUAD. Herein, nearly all *EGFR*-mt cases were mSig^+^, a signature enriched in *NKX2-1*/*TP63* coexpressing bronchioid cells. Importantly, mSig^+^ is not unique to *EGFR* mutations; we report that canonical RAS mutations can also yield this profile. Thus, while mSig^+^ is critical for certain bronchioid tumors, it is not synonymous with the bronchioid subtype. Roughly half of bronchioid tumors are mSig^–^, many driven by canonical RAS mutations signaling with MAPK repressor DUSP4. Another quarter lack both mSig^+^ and canonical RAS mutations but appear to follow DUSP4-driven signaling.

Prior work links bronchioid tumors to a more peripheral TRU phenotype (20). TRU refers to the distal lung, respiratory bronchioles, alveolar ducts, and alveoli, where gas exchange occurs. The bronchioid subtype was historically associated with “bronchioloalveolar carcinoma,” often reflecting lepidic or differentiated LUAD histology, sharing associations such as *EGFR* mutation, nonsmoker status, higher *NKX2-1* expression, and favorable outcomes (21, 23). This report strengthens the view that these diverse phenotypes, genotypes, and morphologies may reflect a unified molecular classification with clinical relevance. At least 75% of bronchioid tumors were *EGFR*- or RAS-driven through mSig^+^ *EGFR* mutation or *K*-EGFR/*K*-Ox RAS mutations. The remaining 25% lacked clear oncogenes but signaling cascades were identified that are similar to *K*-Ox RAS-mt cases. By contrast, squamoid tumors were uniformly *TP53*-mt, showing moderate-to-high *EGFR* expression, which is atypical, as these tumors are overwhelmingly EGFR mSig^–^. The squamoid transcription factor profile reflects reduced *NKX2-1* dependence, consistent with a subset of LUAD but rarely contextualized alongside mutation patterns, as in this report. About half of squamoid tumors display evidence of combined *TP53* mutations with canonical RAS mutations, a known pattern that we show is linked to this expression subtype. The other half are oncogene negative but display signaling distinct from bronchioids, as mSig^–^ squamoids are *DUSP4*-low, unlike the *DUSP4*-high mSig^–^ bronchioids. The magnoid subtype, historically associated with large cell undifferentiated carcinoma histology, exhibited very low expression of lung lineage markers (*NKX2*-1, *TP63*) and absent *EGFR* expression. Magnoids are a large cell–like histologic subtype of LUAD, and they fall into 2 genotypes. Roughly half of the magnoid samples carry RAS mutations with *STK11* and/or *KEAP1* mutations, combined with high *DUSP4* and *TP53* WT status. The remainder of tumors from the magnoid subtype were *TP53*-mt and either were oncogene-negative or had a comutation for *KEAP1*/*STK11*, with moderate *DUSP4* expression. It is plausible to expect that the oncogene-negative magnoids would demonstrate signaling unique to that subtype (and different from that of bronchioid and squamoid) for the purposes of modeling and therapy.

The discovery of *EGFR* mutations as therapeutic targets in LUAD paralleled landmark findings such as *BCR*-*ABL* fusions in chronic myelogenous leukemia (CML) and *HER2* amplifications in breast cancer, fueling modern cancer genomics (51–53). The focus on druggable somatic variants as the basis for LUAD classification has been especially productive in LUAD patient care, but this focus may overshadow the role of lineage and differentiation state for understanding tumorigenesis and model systems. Treatment focus in leukemia illustrates the alternative approach of emphasizing the role of lineage and differentiation state, as opposed to simply focusing on druggable targets, as has been the case in LUAD. For example, the *BCR*-*ABL* fusion defines CML as a disease, as well as defining a variety of therapeutic pathways (54–57). Similarly, the transcription factor fusion, *PML*-*RARA*, defines acute promyelocytic leukemia, where differentiation arrest occurs at the promyelocyte stage (58). Sarcomas also demonstrate lineage-defining lesions, such as *SS18*-*SSX* in synovial sarcoma and *EWSR1*-*ATF1* in clear cell sarcoma (36, 59, 60). In epithelial tumors, oncogenic variants often align with the cell of origin and differentiation. Salivary gland cancers exemplify this: secretory carcinoma harbors *ETV6*-*NTRK3* fusions in >95% of cases (61); the ductal variant shows *AR* overexpression in >70% and *HER2* (62); mucoepidermoid carcinoma has *CRTC1*-*MAML2* (63). While salivary carcinomas showcase striking correlations between morphologic subtype and molecular lesion, many solid tumors with less distinct morphology can also be subclassified through molecular profiling, which reveals underlying cell of origin. Copy number alterations also show tissue specificity, embedding canonical targets within tumor-specific genomic patterns. Thus, oncogenesis is not random but rather highly tissue-context dependent: drivers active in one tissue may be inert in another, even when pathways overlap.

Just as transcriptional and genomic features distinguish tumor subtypes, oncogenic signaling behavior is context dependent (64). *BRAF* mutations, for example, are targetable in melanoma but resistant in colorectal cancer (65). Similarly, EGFR kinase mutations are restricted to LUAD, despite *EGFR* amplification in other tumors. If universally oncogenic, EGFR kinase mutations would appear across tissues; instead, their selection depends on lineage, reinforced by coexpression of *NKX2-1* (23). *EGFR* mutations also align with expression subtypes, mirroring lineage-dependent subgroups in breast cancer (66). Histopathology echoes this, with differentiated morphologies in bronchioids and solid/undifferentiated morphologies in magnoids. Together, these findings emphasize that cell identity, defined by origin, differentiation, and lineage factors, shapes malignant phenotypes. Even without clear histology, molecular lesions can reveal lineage, as in triple-negative breast cancer.

Although substantial effort has been devoted to identifying new therapeutic targets, many known drivers remain difficult to target, such as transcription factors (*TP63*, *MYC*), hormone receptors, and “hard-to-drug” genes like RAS and *PIK3CA* (20, 67–72). Some candidates (e.g., FGFR inhibitors) face challenges due to rarity, low efficacy, or toxicity. Thus, LUAD drug development is often illustrated as a pie chart in which small slices represent sparse subsets of “oncogene-positive” cases, while a large portion remains “other genes” or “oncogene-negative.” This pie chart model is appealing but it is oversimplified by lumping together oncogene-negative tumors and ignoring cooccurrence patterns (e.g., RAS with *KEAP1*/*STK11*). The pie chart approach fails to highlight expression subtype contexts, making therapeutic translational research even more difficult (Supplemental Figure 10) (73). Therefore, we proposed a layered cake concept, integrating oncogenes, mutations, and signaling cascades across multiple layers that together lead to oncogenesis and tumor subtype phenotypes.

This study proposes a lineage-directed framework integrating driver genes and expression subtypes. By examining EGFR signaling, LUAD gene expression subtypes, and canonical drivers, we developed an integrated model (Figure 4) that offers an improved structure compared with the pie chart. Oncogene-negative cases are stratified by subtype context, and difficult targets like *KRAS* are logically grouped. Although the multiple strata suggested by this model would challenge clinical trialists, this framework reflects the heterogeneity of the disease and appears feasible and comparable in complexity to other heterogeneous diseases such as leukemia with its many lineage-driven subsets. The framework shown by Figure 4 is intended to emphasize selected aspects of cell lineage and signaling and is not intended to be comprehensive. Complementary classifications or refinements are expected that might include histologic components (such as mucinous morphology) or druggable targets such as KRAS G12C (74).

In summary, *EGFR* mutations are tightly linked to the mSig^+^ pattern, especially in bronchioid LUAD, though RAS mutations can also generate this profile. Magnoid tumors show little or no mSig^+^ signal, even with *EGFR* mutations, while squamoid tumors express *EGFR* abundantly yet remain mSig^–^. These findings underscore that LUAD taxonomy is best understood through lineage and comutation context. Our framework illustrates how clinically relevant groups can be defined without overwhelming sparsity, potentially reframing “oncogene-negative” tumors within a broader taxonomy. Even absent druggable targets, such stratification may enable more personalized treatment approaches.

## Methods

### Sex as a biological variable.

This study did not consider sex as a biological variable. Both male and female patients were included in the analyzed cohorts; however, the analyses were not stratified by sex because the objective was to identify *EGFR*-associated genomic and transcriptomic signatures independent of sex. The findings are therefore expected to be broadly applicable regardless of sex.

### Data acquisition.

For the purposes of generating expression signatures of *EGFR* activation, primary gene expression data were obtained from multiple independent sources, including RNA characterization, DNA mutation data (*EGFR* mutation at a minimum), and clinical characteristics (21, 75). Cohort eligibility included the requirement that patients were primarily selected as part of an initial diagnosis and were previously untreated. Only validated kinase-activating mutations in exons 18–21 of *EGFR* were considered as mutated for the purpose of the study. To support the generalizability of expression signatures, we obtained RNA derived from 3 complimentary technology platforms: Illumina 1-color gene expression arrays (MSKCC, *n* = 192, GSE68465, ref. 75, and TSP, *n* = 41, GEO GSE12667) (76), Agilent 2-color gene expression arrays (UNC, *n* = 73, GEO GSE36471) (21), and a cohort based on a next-generation mRNA-seq protocol (TCGA, *n* = 486, Genomic Data Commons [GDC] Portal) (77). The MSKCC cohort was selected as the training dataset, and all other cohorts were withheld as independent validation cohorts. For additional integrated analysis, we considered copy number and additional mutation data from TCGA datasets. For integrative analysis, mutation calls and copy number of selected driver oncogenes, including *EGFR*, *TP53*, *CDKN2A*, *KEAP1*, *STK11*, *KRAS*, *NRAS*, *HRAS*, *BRAF*, *HER2*, *ALK*, *MET*, and *ROS1*, were obtained from prior published datasets and curated (44). Other genes and driver mutation data shown in Supplemental Table 6 were downloaded from cBioPortal (78, 79).

### Data preprocessing.

Pipelines for RNA processing and normalization have been previously reported (21, 22). Briefly, Agilent 2-color gene expression array assays were background corrected and normalized according to standard protocols of the platforms. Specifically, for Agilent 2-color microarray MSKCC and UNC data, Lowess normalization was applied to obtain expression values. For Affymetrix 1-colored microarray TSP data, the robust multiarray average algorithm was applied for background correction and normalization. Each dataset was then subjected to median centering. For TCGA LUAD mRNA-seq data, RNA-seq by expectation-maximization–normalized expression data (fragments per kilobase of transcript per million mapped reads) was obtained (80). All datasets were *z*-score normalized before visualization in heatmaps in R version 4.3.1 (81). For all other analyses, quantile normalization was applied via preprocessCore R package 1.64.0 (82).

### Establishment of EGFR mSig.

To investigate the role of gene expression in predicting *EGFR* mutation, we calculated the Δ score for each gene in the supervised testing *EGFR*-mt tumors versus *EGFR* WT tumors using the training cohort (MSKCC) by the significance analysis of microarray R package (SamR, version 3.0) (83). In this manner, genes could be ranked for positive and negative correlation with mutation status. In parallel, we generated a family of centroid-based predictors for mt versus WT using the ClaNC method (84, 85).

To visualize expression profiles of each molecular subtype, semisupervised clustering was used and visualized via customized heatmap function based on gplots R package (version 3.1.3.1) (86) and complexHeatmap R package (version 2.18.0) (87, 88). We applied 6 machine learning (ML) algorithms to validate EGFR mSig prediction performance — Support Vector Machines, with different kernel functions, including Linear, Polynomial, and Radial Basis Function, and the Ensemble Learning algorithms, Random Forest, AdaBoost, and LogitBoost. The top 1,000 genes were selected using 2-tailed *t* test and were used as input features for ML. We used the receiver operating characteristic (ROC) curve based on 10-fold cross-validation to evaluate the performance of ML models. The area under the ROC curve values were calculated and used as the primary performance metric. Other performance metrics, including sensitivity, specificity, accuracy, PPV, and NPV were also calculated. For external validation (or independent testing), the ML performances were further tested using different combinations of training and testing sets.

For the purpose of attributing biologic relevance to lists of genes developed in the study, we used Database for Annotation, Visualization and Integrated Discovery (DAVID) and GSEA (version 4.3.2) (89–91). Gene lists derived from DAVID were documented in terms of enrichment for functional gene groups by biological pathways, and statistical significance (*P* < 0.05 or highly enriched fold enrichment >2) was assessed for the purpose of visualization using the dplyr R package (version 1.1.3) (92) and ggplot2 R package (version 3.5.1) (93).

### Prediction performance test.

In prediction performance tests, *EGFR*-mt/mSig^+^ patients were classified as true positives, *EGFR* WT/mSig^–^ patients as true negatives, *EGFR* WT/mSig^+^ patients as false positives, and *EGFR*-mt/mSig^–^ patients as false negatives. The performance test was calculated in R (version 4.3.1.) (81) and visualized via caret R package (version 6.0.94) (94).

### Statistics.

In patient demographic data, molecular subtype, stage at diagnosis, and smoking history were tested by χ^2^ test. Pack-years were tested by 1-way ANOVA. Overall survival data from the MSKCC cohort were summarized with Kaplan-Meier survival curves. Overall survival was defined as the time from diagnosis of NSCLC to death from any causes or time of last follow-up within 36 months. Right censoring was applied to the last follow-up time point where no event (death) had occurred. Visualization of the curves and calculation of the log rank *P* value was conducted via survival R package (version 3.7.0) (95, 96). To assess mutual exclusivity between two events, OR was calculated, and the *P* values for mutual exclusivity were calculated by Fisher’s exact test. OR and *P* values were visualized in heatmaps via ComplexHeatmap (R package version 2.18.0) (87, 88). All data handling and statistical analyses were performed in the R version 4.3.1. environment (81). A *P* value of less than 0.05 was considered significant.

### Study approval.

This study was reviewed and approved by the University of Tennessee Health Science Center Institutional Review Board (25-10419-NHSR).

### Data availability.

The data analyzed in this study were obtained from Gene Expression Omnibus (GEO) at GSE68465, GSE12667, and GSE36471 as well as GDC Portal) (77). LUAD cell line dependency score, omics datasets (hotspot mutation, gene-level copy number, and log_2_ TPM gene expression data, all release 25Q3), and Sanger GDSC1 drug response data were obtained from DepMap (41–43). All software used for EGFR mSig score calculation is available at GitHub (https://github.com/hayeslab/EGFRmSig). Values for all data points in graphs are reported in the Supporting Data Values file.

## Author contributions

Funding acquisition: YC, LM, and DNH. Supervision: LM and DNH. Conceptualization: MK, WL, KAH, MPS, and DNH. Data collection: MK, WL, HJ, HYC, MDW, MCH, KAH, and DNH. Data generation and curation: MK, WL, HJ, and DNH. Writing: MK, LM, KAH, MPS, and DNH. Code availability: MK and MY. Reviewing and editing: all authors. All authors approved the final version of the manuscript. MK and WL contributed equally as co–first authors; the order was determined by MK’s role in finalizing the study.

## Conflict of interest

DNH and MDW have patents (US 10,829,819 B2 and US 10,934,595 B2) on lung cancer and molecular markers.

## Funding support

This work is the result of NIH funding, in whole or in part, and is subject to the NIH Public Access Policy. Through acceptance of this federal funding, the NIH has been given a right to make the work publicly available in PubMed Central.

- NCI of the NIH under U01CA272541 (to LM), which is part of the Metabolic Dysregulation and Cancer Risk Program Consortium; NCI R01CA262296 (to YC and DNH); U24CA264021 (to DNH); UG1CA233333 (to DNH); and NCI R01CA211939 (to DNH).

## Supplementary Material

Supplemental data

ICMJE disclosure forms

Supporting data values

## Figures and Tables

**Table 1:**
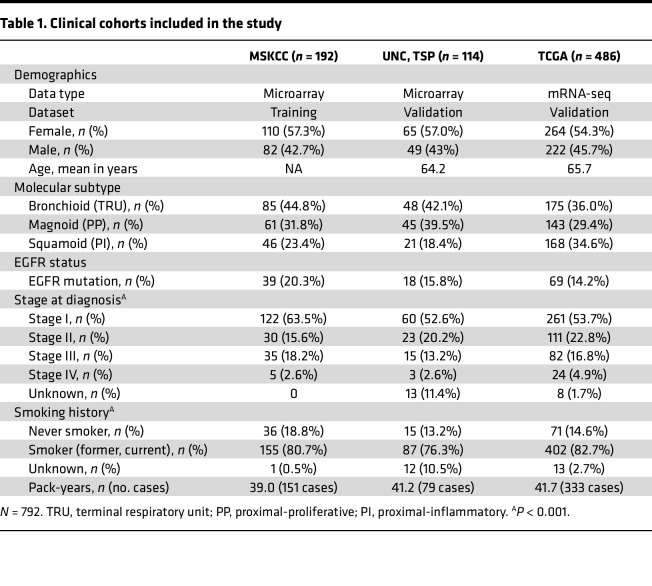
Clinical cohorts included in the study

## Version 1 03/12/2026

In-Press Preview

## Version 2 04/22/2026

Electronic publication
