# Supplementary material for: RAS signaling in lung adenocarcinoma is defined by lineage context and DUSP4 loss
Source: JCI Insight. 2026 Mar 12;11(8):e200912. doi: 10.1172/jci.insight.200912 (PMC13135389; doi:10.1172/jci.insight.200912)
Supplement: Supplemental data [file jciinsight-11-200912-s192.pdf]

Supplemental Data

RAS signaling in lung adenocarcinoma is defined by lineage context and *DUSP4* loss

Minjeong Kim, Wisut Lamlertthon, Heejoon Jo, Yan Cui, Miyeon Yeon, Hyo Young Choi, Katherine A. Hoadley, Matthew P. Smeltzer, Michele C. Hayward, Matthew D. Wilkerson, Liza Makowski, D. Neil Hayes.

Supplemental Figures

S1. Machine learning-based performance evaluation and validation of the EGFR mSig

S2. Selection and validation of EGFR mSig genes

S3. Pathway enrichment analysis of EGFR mSig genes

S4. Lineage-defined molecular subtypes of LUAD and its interplay with other transcription factors

S5. Co-occurrence and mutual exclusivity of selected genomic alterations

S6. Differentially expressed genes in the Bronchioid subtype and distinctive molecular profiles defined by the EGFR mSig

S7. Integrative analysis of genomic alterations and gene expression across molecular subtypes of LUAD cell lines

S8. Integrative analysis of genomic alterations and gene expression across molecular subtypes of LUAD (*KRAS* mutation only)

S9. EGFR mSig stratifies predicted gefitinib sensitivity beyond mutation status

S10. Conventional pie chart of oncogenic driver gene mutation frequencies in LUAD

Supplemental Figure S1. **Machine learning-based performance evaluation and validation of the EGFR mSig.**

The top 1,000 genes selected by t-test were used as input features for 10-fold cross-validation. Receiver operating characteristic (ROC) curves of six different machine learning models are shown in distinct colors. The *x*-axes represent false positive value (1-specificity). The *y*-axes represent true positive value (sensitivity). (**A**) MSKCC, (**B**) UNC + TSP, (**C**) MSKCC + UNC + TSP (**D**) TCGA, (**E**) Training: MSKCC, Validation: UNC+TSP, (**F**) Training: UNC+TSP, Validation: MSKCC.

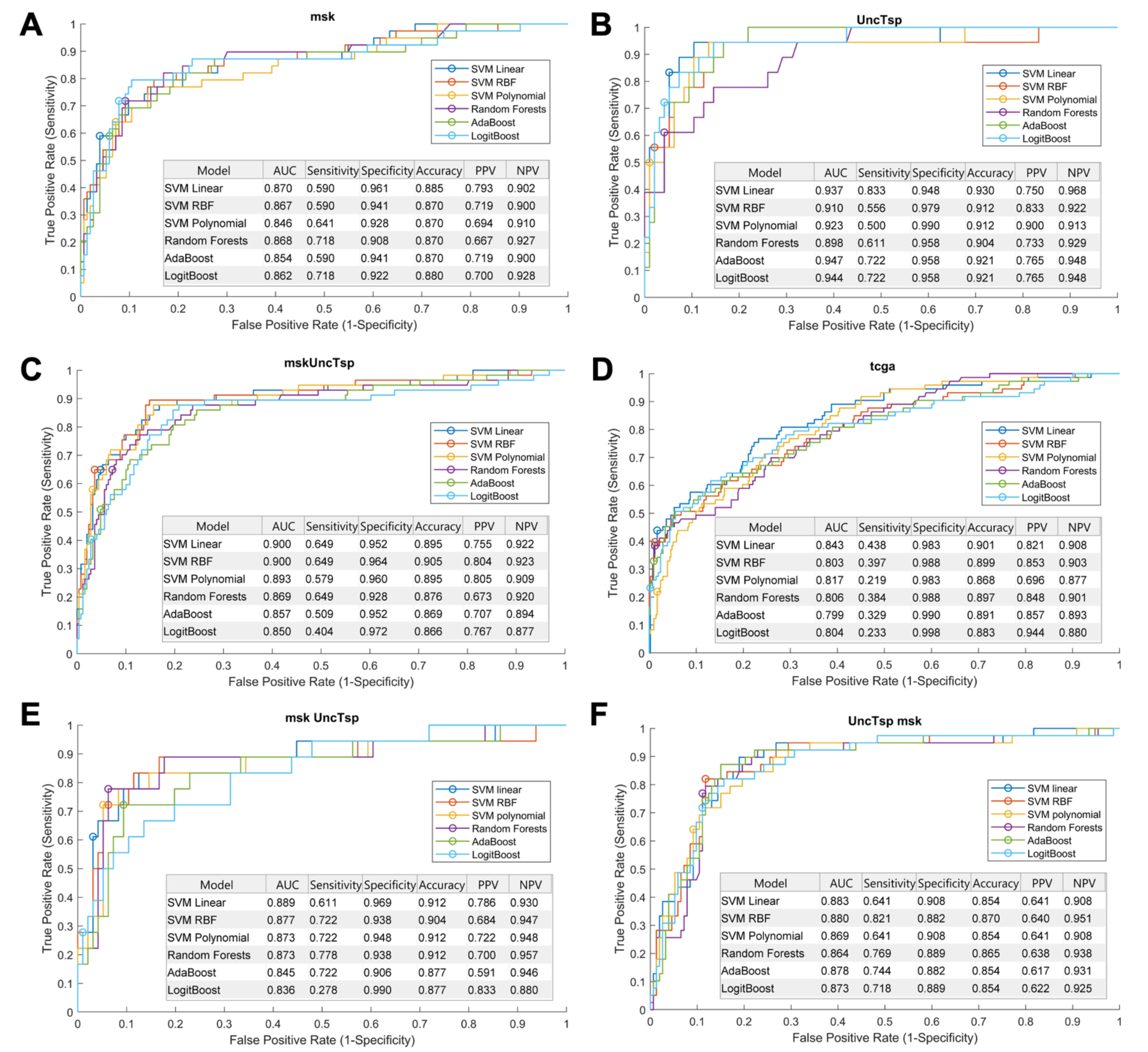

Supplemental Figure S2. Selection and validation of EGFR mSig genes.

(**A**) DEGs between *EGFR*-mt and EGFR WT LUAD samples were identified using SamR, and only statistically significant DEGs with a false discovery rate (FDR) < 1% were selected as candidate signature genes. The *x*-axis represents expected SamR score. *y*-axis represents observed score. (**B**) Cross-validation of *EGFR*-related signature genes was performed using ClaNC. A total of 1,020 genes were selected as the final EGFR mSig based on the lowest classification error rate (Error rate < 0.2). Red and green lines indicate different patient groups used in the classification model. The *x*-axis represent genes per class. The *y*-axis represents cross-validation error rate.

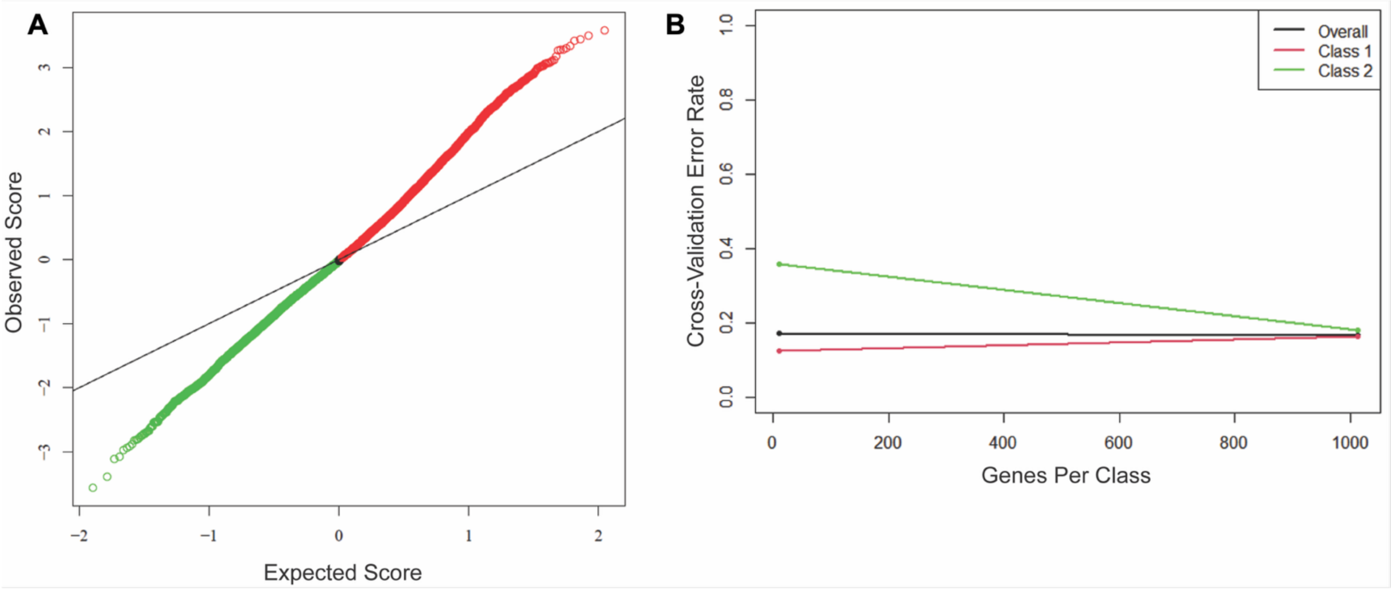

Supplemental Figure S3. Pathway enrichment analysis of EGFR mSig genes.

Biological pathways associated with 1,020 *EGFR* mSig genes (690 upregulated, 330 downregulated) were analyzed using the web-based DAVID functional annotation tool. Gene Ontology (GO) term enrichment results are visualized in a dot plot. The x-axis indicates fold enrichment for each pathway, dot size represents the number of genes involved, and dot color reflects statistical significance based on p-values.

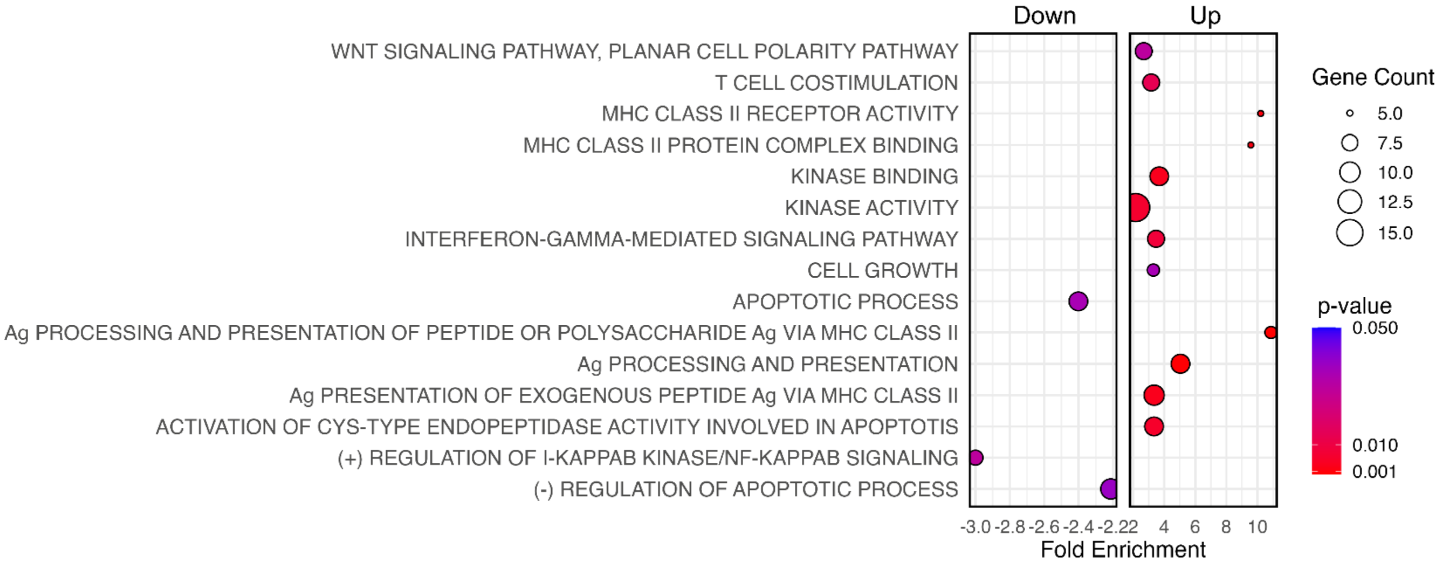

Supplemental Figure S4. Lineage-defined molecular subtypes of LUAD and its interplay with other transcription factors.

Samples (n = 486, TCGA LUAD) are represented in columns and grouped by molecular subtype. Sample orders are retained as shown in Figure 3. Gene expression differences were assessed using one-way ANOVA. Statistical significance reflects subtype-specific differences in the genomic features (****, p < 0.0001). ge, gene expression.

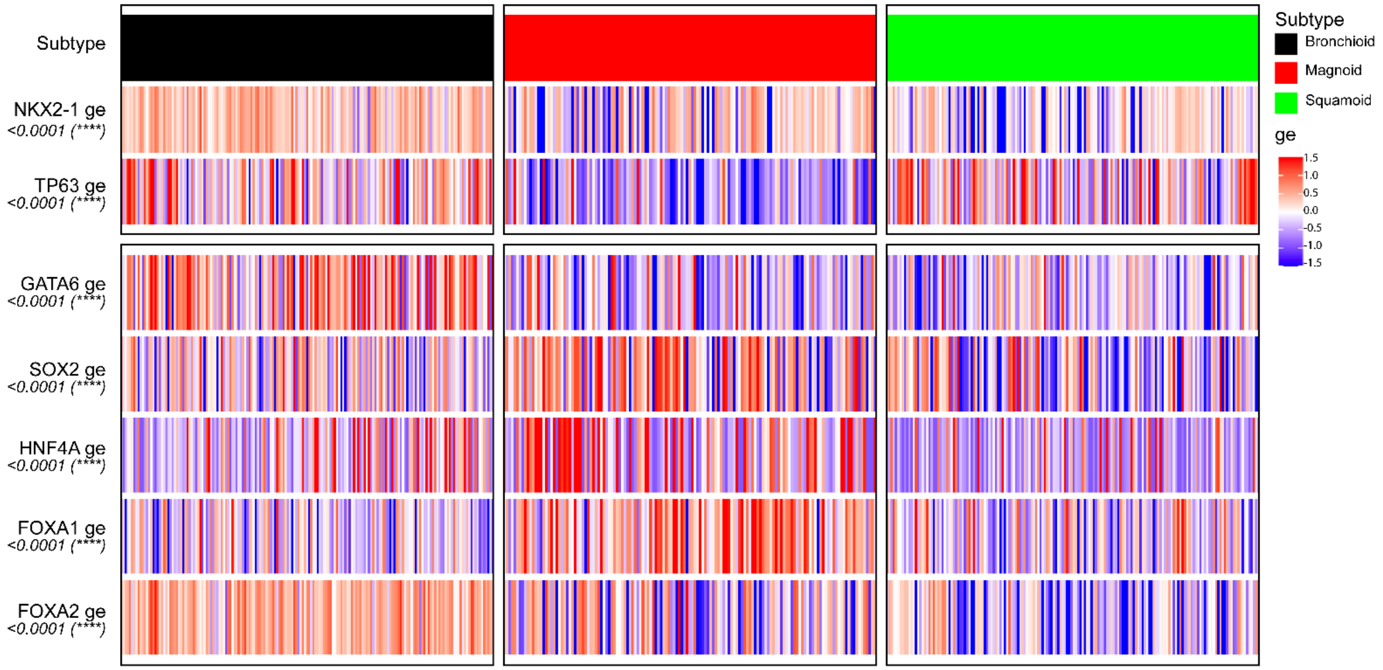

Supplemental Figure S5. Co-occurrence and mutual exclusivity of selected genomic alterations. Heatmaps display odds ratios for the co-occurrence of genomic events between pairs of genes, with color indicating the magnitude of the odds ratio. Co-occurring events exhibit odds ratios greater than 1, while mutually exclusive events have odds ratios less than 1. The strength of mutual exclusivity (or co-occurrence) increases as the odds ratio deviates further from 1. P-values were calculated using Fisher's exact test. ge, gene expression; mt, mutant; CN, copy number. (**A**) Global comparison across all LUAD samples. (**B-D**) Subtype-specific analyses of mutual exclusivity within the (**B**) Bronchioid, (**C**) Magnoid, and (**D**) Squamoid subtypes.

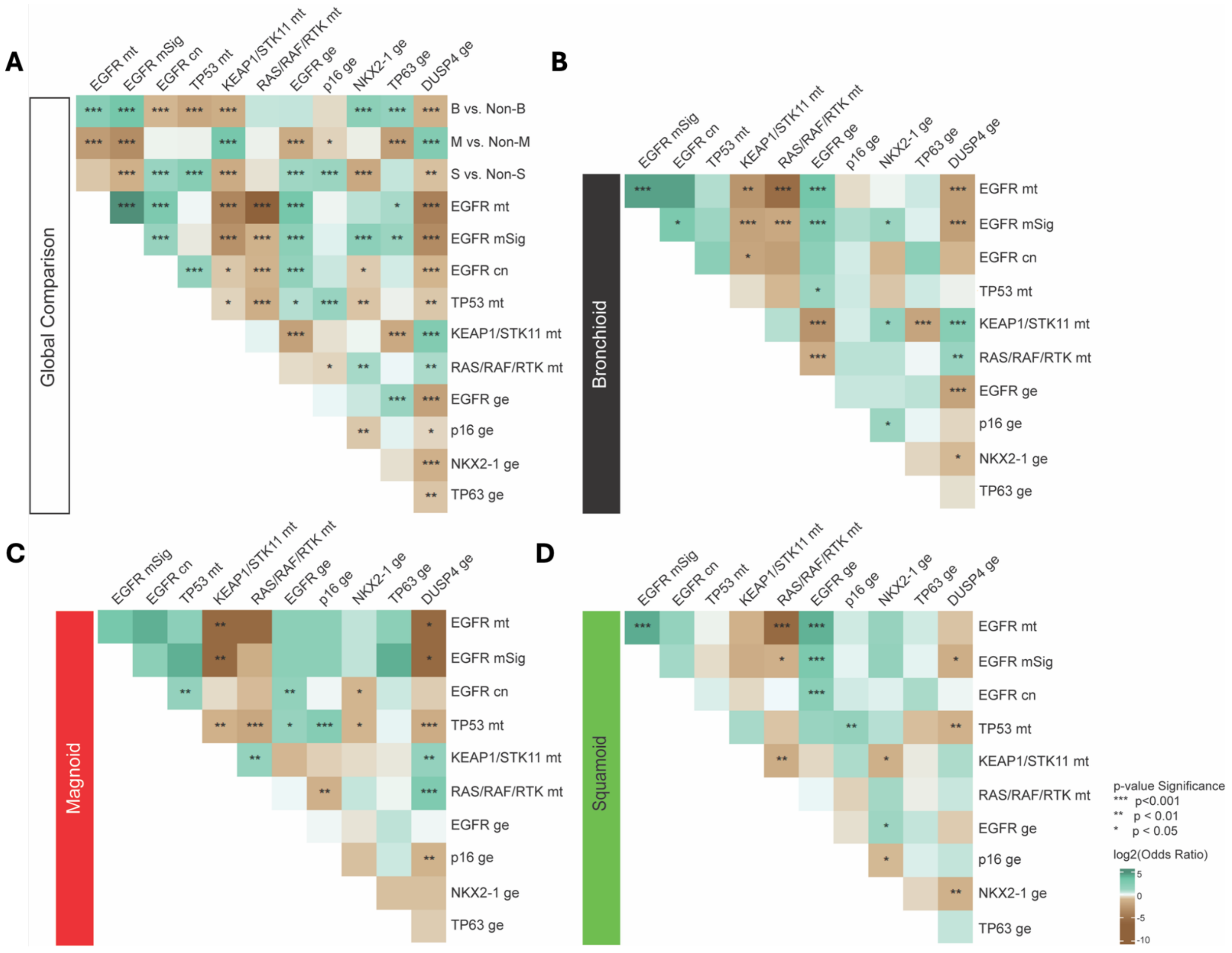

Supplemental Figure S6. Differentially expressed genes in the Bronchioid subtype and distinctive molecular profiles defined by the EGFR mSig.

(**A**) Dot plot showing the distribution of delta scores calculated by SamR for 11,807 genes. Each dot represents a gene, with the x-axis showing the delta score for genes in the *EGFR*-predicted group across all LUAD subtypes, and the y-axis showing the delta score for genes in the *EGFR*-predicted group specifically within the Bronchioid subtype. Genes are colored based on empirically chosen cutoff values indicated by red vertical lines (delta score > 1.6 or < -1.7) and blue horizontal lines (delta score > 1.5 or < -1.7), capturing statistically significant differential expression. The 221 genes highlighted in color were selected for further analysis shown in **B** and **C**. (**B**, **C**) Heatmaps display gene expression profiles of the selected 221 genes across different LUAD subtypes, with top annotations for *EGFR* mutation status and *EGFR* prediction in the (**B**) MSKCC and (**C**) TCGA cohort. Each row represents one gene, with the left color bar indicating subgroup classification from **A**. Dot colors correspond to gene expression patterns as indicated by the heatmap color bars.

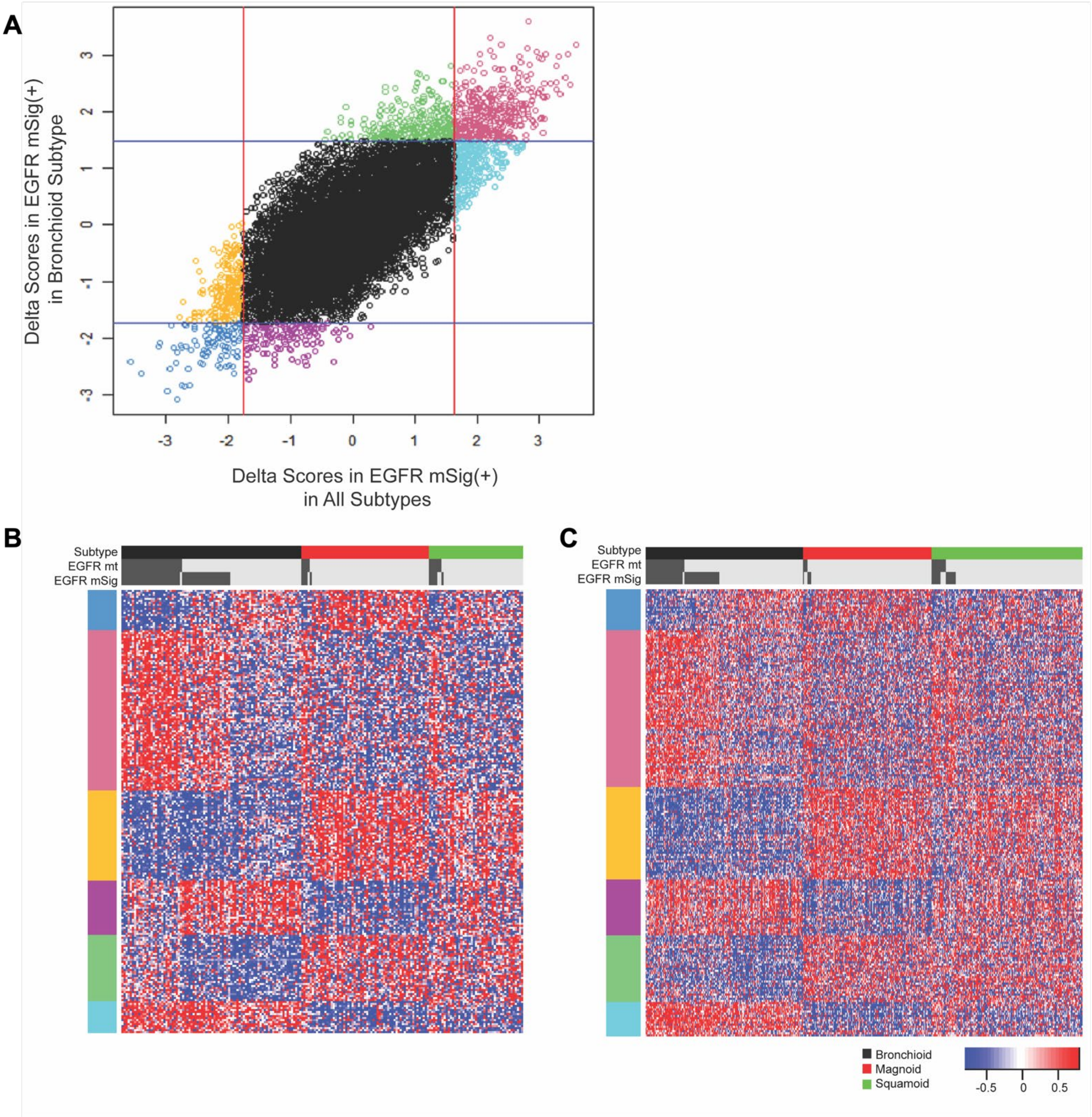
A
Delta Scores in EGFR mSig(+)
in Bronchioid Subtype
Delta Scores in EGFR mSig(+)
in All Subtypes
-3
-2
-1
0
1
2
3
B
C
Subtype
EGFR mt
EGFR mSig
Bronchioid
Magnoid
Squamoid
-0.5
0
0.5

Supplemental Figure S7. Integrative analysis of genomic alterations and gene expression across molecular subtypes of LUAD cell lines.

Samples (n = 45, DepMap LUAD cell lines) are represented in columns and grouped by molecular subtype. RAS/RAF/RTK includes *KRAS*, *HRAS*, *NRAS*, *BRAF*, *HER2*, *MEK*, and *ALK*. ge, gene expression; WT, wild-type; mt, mutant; CN, copy number.

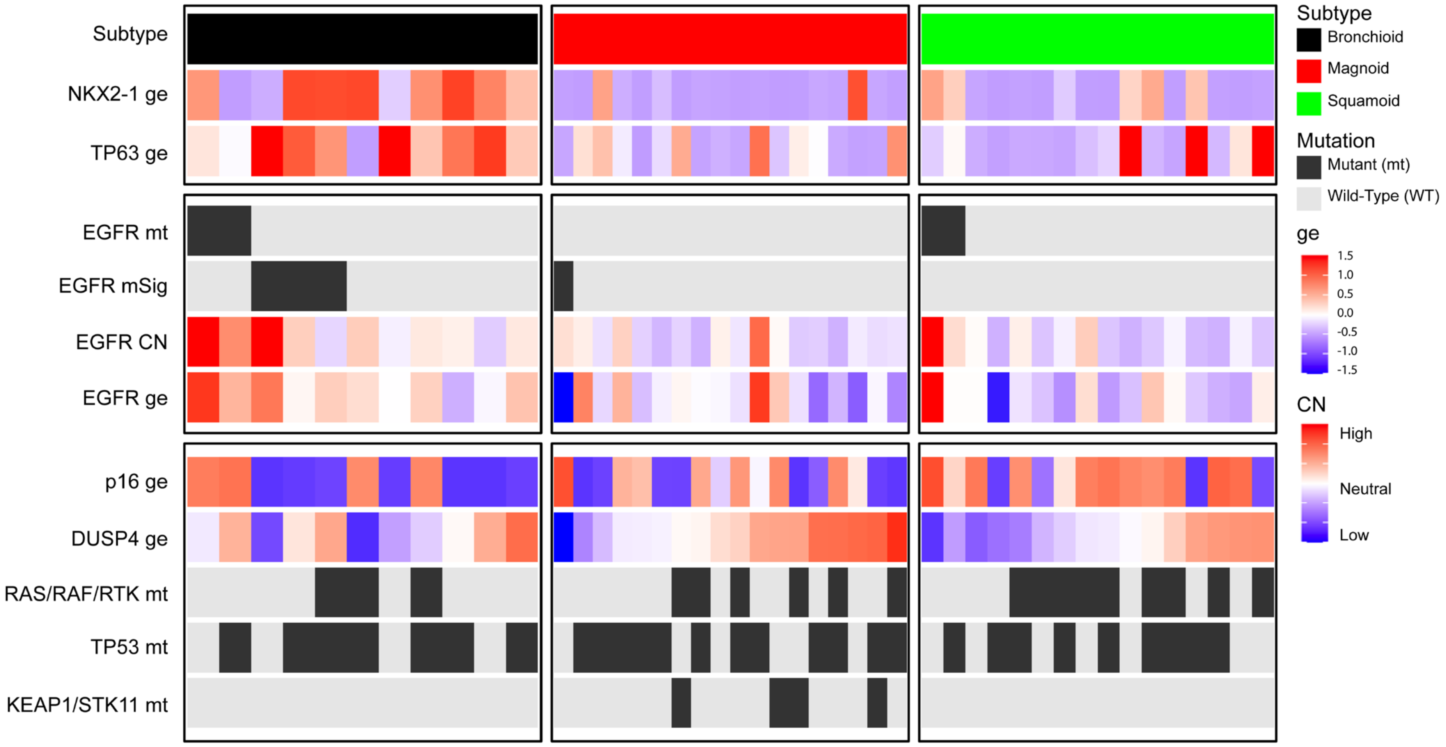

Supplemental Figure S8. Integrative analysis of genomic alterations and gene expression across molecular subtypes of LUAD (*KRAS* mutation only).

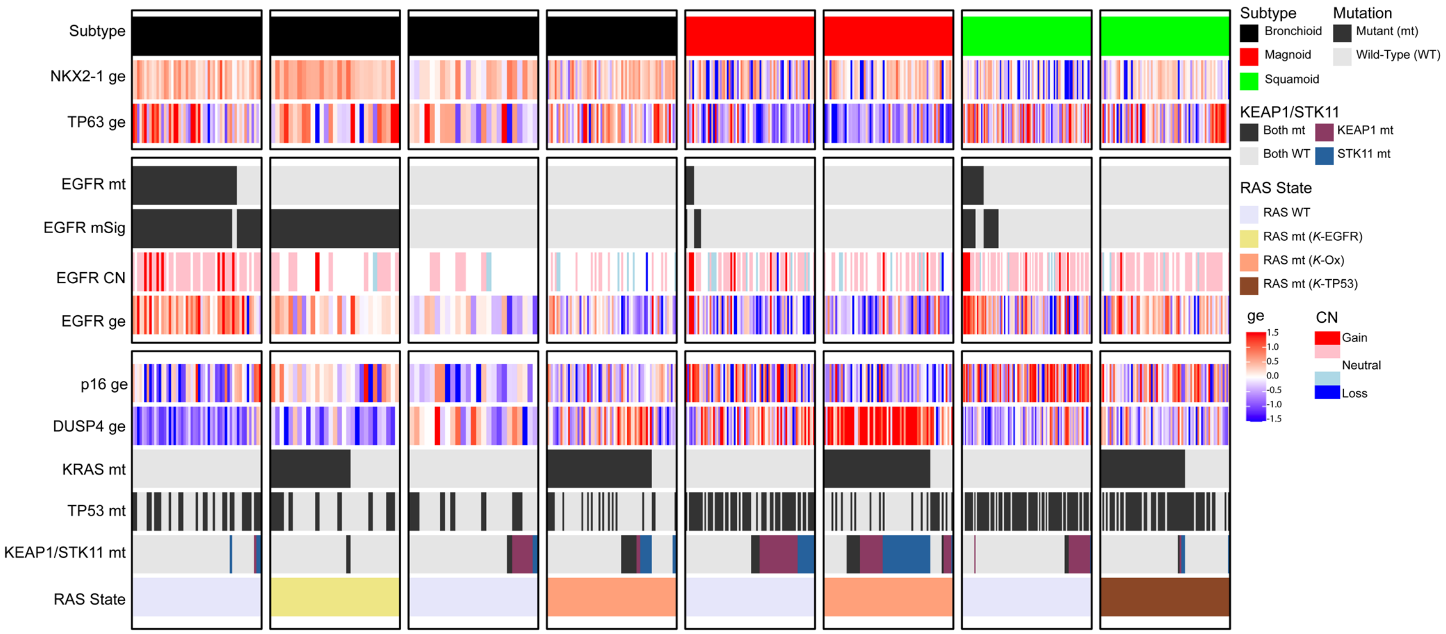

Supplemental Figure S9. EGFR mSig stratifies predicted gefitinib sensitivity beyond mutation status.

Predicted gefitinib sensitivities were compared among the five groups using the Kruskal-Wallis test (two-sided P).

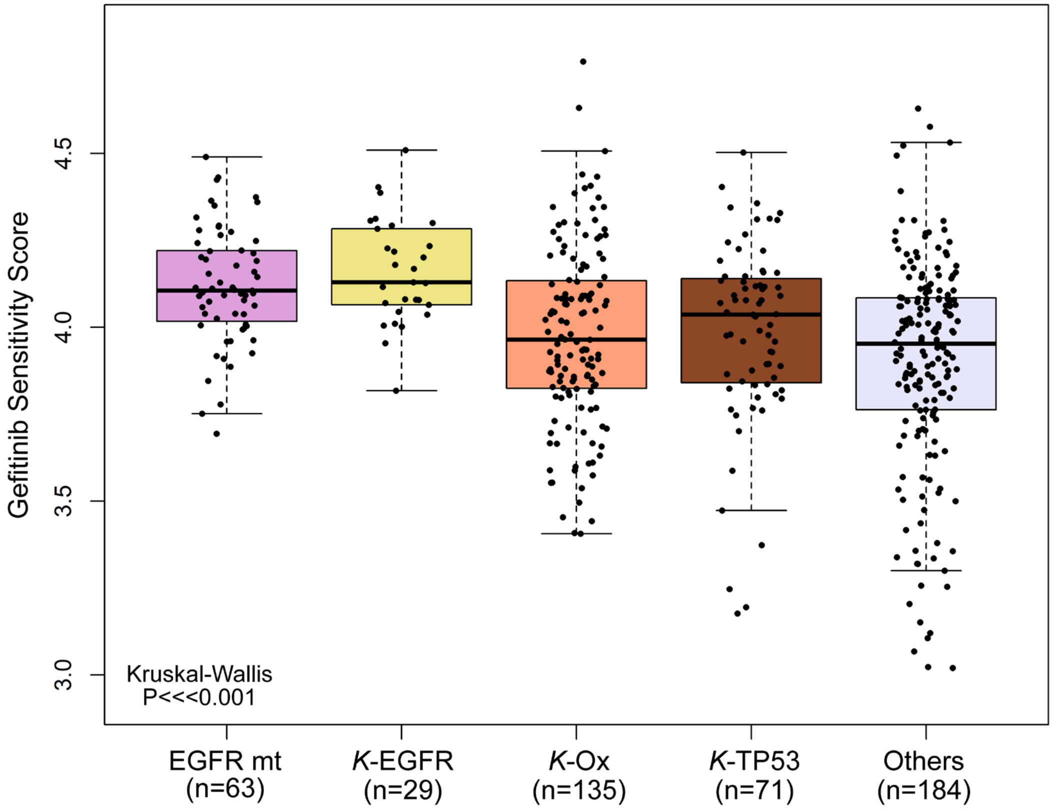

Supplemental Figure S10. Conventional pie chart of oncogenic driver gene mutation frequencies in LUAD.

This figure is presented to exemplify the traditional use of pie charts for mutation frequency representation (TCGA PanCancer Atlas LUAD, dataset from cBioPortal).

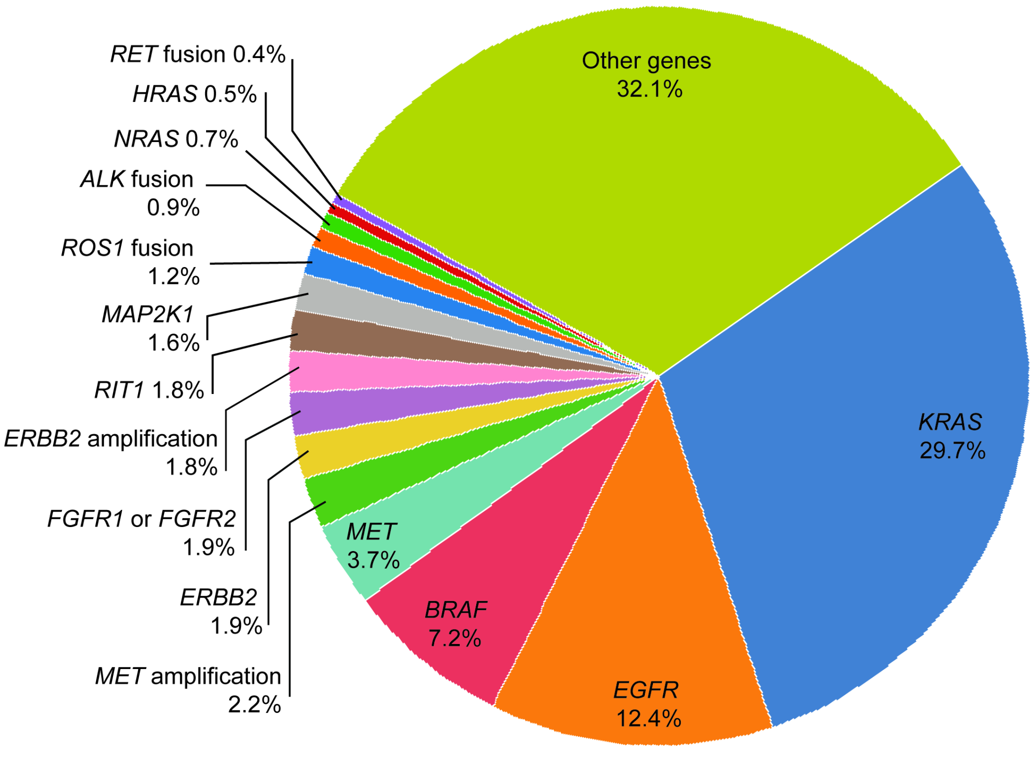

Supplemental Tables

S1. EGFR mutation signature (EGFR mSig) gene list in ranked order

S2. Performance test results between EGFR-like mutant group and wild-type group based on *EGFR*-related gene signature

S3. Association between mSig status and histological subtypes

S4. Odds Ratio and p-value; each subtype vs. all others

S5. Odds Ratio and p-value; within subtypes

S6. Driver mutation index of TCGA LUAD samples

Supplemental Table S1. EGFR mutation signature (EGFR mSig) gene list in ranked order.

Rank	*Gene*	Delta Score	Fold Change	q-value
1	HIP1	3.58	2.21	0.00
2	*PIGQ*	3.49	2.17	0.00
3	C16orf58	3.44	2.15	0.00
4	*UPK3A*	3.41	2.14	0.00
5	HMOX2	3.33	2.10	0.00
6	*RNF40*	3.30	2.09	0.00
7	GALNT10	3.28	2.08	0.00
8	*KIAA0319L*	3.27	2.08	0.00
9	KIAA0494	3.27	2.07	0.00
10	*GPR177*	3.17	2.03	0.00
11	C1orf149	3.11	2.01	0.00
12	*EGFR*	3.10	2.01	0.00
13	FLJ14154	3.08	2.00	0.00
14	*FTSJ2*	3.07	1.99	0.00
15	MKL2	3.06	1.99	0.00
16	*LRRC31*	3.06	1.99	0.00
17	KIAA0495	3.05	1.99	0.00
18	*RERE*	3.02	1.98	0.00
19	B4GALT7	3.02	1.98	0.00
20	*ADCY9*	3.02	1.97	0.00
21	LRRC47	3.01	1.97	0.00
22	*C1orf174*	2.99	1.96	0.00
23	HHLA3	2.99	1.96	0.00
24	*GPR172B*	2.98	1.96	0.00
25	PEX10	2.96	1.95	0.00
26	*CTF1*	2.94	1.94	0.00
27	GUSB	2.93	1.94	0.00
28	*LDLRAP1*	2.89	1.92	0.00
29	ROGDI	2.89	1.92	0.00
30	*FLJ10986*	2.89	1.92	0.00
31	LYRM1	2.88	1.92	0.00
32	*ZNF688*	2.87	1.91	0.00
33	HIRIP3	2.86	1.91	0.00
34	*PHLDB1*	2.86	1.91	0.00
35	PPFIBP2	2.85	1.91	0.00
36	*PPIE*	2.85	1.90	0.00
37	TRAPPC3	2.84	1.90	0.00
38	*MMP15*	2.84	1.90	0.00
39	COL21A1	2.81	1.89	0.00
40	*NSUN5*	2.81	1.89	0.00
41	VWA1	2.80	1.89	0.00
42	*RHCE*	2.79	1.88	0.00
43	RAD51L1	2.79	1.88	0.00
44	*PRKCSH*	2.79	1.88	0.00
45	GTF2I	2.79	1.88	0.00
46	*KCNK5*	2.78	1.88	0.00
47	THUMPD1	2.78	1.88	0.00
48	*CCDC101*	2.77	1.87	0.00

49	PDXDC1	2.77	1.87	0.00
50	*PIGV*	2.75	1.87	0.00
51	MGRN1	2.75	1.87	0.00
52	*ZNF263*	2.74	1.86	0.00
53	RPA2	2.72	1.85	0.00
54	*SLC29A1*	2.72	1.85	0.00
55	PARK7	2.72	1.85	0.00
56	*TBL3*	2.71	1.85	0.00
57	ZFP36L1	2.71	1.85	0.00
58	*HMGCL*	2.71	1.85	0.00
59	PEF1	2.71	1.85	0.00
60	*GGA2*	2.70	1.85	0.00
61	ANKMY2	2.70	1.84	0.00
62	*FAAH*	2.69	1.84	0.00
63	LRRC41	2.69	1.84	0.00
64	*IFT140*	2.68	1.84	0.00
65	TELO2	2.68	1.84	0.00
66	*ATP9A*	2.67	1.84	0.00
67	HSPB1	2.67	1.83	0.00
68	*MANBA*	2.67	1.83	0.00
69	TRMT5	2.67	1.83	0.00
70	*CTNNBIP1*	2.67	1.83	0.00
71	SALL2	2.66	1.83	0.00
72	*PTK7*	2.66	1.83	0.00
73	PRNPIP	2.66	1.83	0.00
74	*RPS6KA1*	2.65	1.83	0.00
75	FUCA1	2.64	1.82	0.00
76	*WDTC1*	2.64	1.82	0.00
77	MR1	2.63	1.82	0.00
78	*C7orf23*	2.63	1.82	0.00
79	LOC643641	2.62	1.81	0.00
80	*IGSF3*	2.61	1.81	0.00
81	VPS13D	2.61	1.81	0.00
82	*LDOC1*	2.61	1.81	0.00
83	OGDH	2.61	1.81	0.00
84	*BCL7C*	2.60	1.81	0.00
85	HYI	2.60	1.81	0.00
86	*SEZ6L2*	2.60	1.81	0.00
87	STX4	2.60	1.81	0.00
88	*H2AFV*	2.60	1.81	0.00
89	CAMTA1	2.60	1.81	0.00
90	*CABIN1*	2.59	1.80	0.00
91	LEFTY2	2.59	1.80	0.00
92	*DDAH1*	2.59	1.80	0.00
93	CTA-216E10.6	2.58	1.80	0.00
94	*NPTXR*	2.58	1.80	0.00
95	ZBTB48	2.57	1.80	0.00
96	*ZDHHC11*	2.55	1.79	0.00
97	NFYC	2.55	1.79	0.00
98	*HLA-DMA*	2.54	1.79	0.00
99	C1orf160	2.54	1.78	0.00
100	*NUBP2*	2.54	1.78	0.00

101	MGC16824	2.54	1.78	0.00
102	*C14orf94*	2.53	1.78	0.00
103	NPC2	2.53	1.78	0.00
104	*TSPAN13*	2.53	1.78	0.00
105	BLVRA	2.53	1.78	0.00
106	*HSD17B8*	2.53	1.78	0.00
107	LYPLA2	2.52	1.78	0.00
108	*GGTLA4*	2.52	1.78	0.00
109	LTBP2	2.51	1.77	0.00
110	*LTBP3*	2.51	1.77	0.00
111	SLC22A18	2.50	1.77	0.00
112	*DIRAS3*	2.50	1.77	0.00
113	TMEM159	2.50	1.77	0.00
114	*HAGH*	2.50	1.77	0.00
115	NUDCD3	2.50	1.77	0.00
116	*AUTS2*	2.49	1.77	0.00
117	BCKDK	2.49	1.77	0.00
118	*CD2BP2*	2.49	1.76	0.00
119	SDC1	2.48	1.76	0.00
120	*PLEKHM1*	2.48	1.76	0.00
121	HCFC1R1	2.47	1.76	0.00
122	*SSBP3*	2.47	1.76	0.00
123	DDEFL1	2.46	1.75	0.00
124	*ZDHHC4*	2.45	1.75	0.00
125	TSPAN6	2.45	1.75	0.00
126	*CA10*	2.44	1.75	0.00
127	ZMIZ2	2.44	1.75	0.00
128	*STOML1*	2.44	1.75	0.00
129	NCALD	2.44	1.75	0.00
130	*MMP24*	2.44	1.75	0.00
131	NME3	2.44	1.75	0.00
132	*ELN*	2.43	1.74	0.00
133	FOXJ1	2.43	1.74	0.00
134	*UROD*	2.43	1.74	0.00
135	ATP6V0A1	2.43	1.74	0.00
136	*CPT2*	2.42	1.74	0.00
137	EFHC2	2.42	1.74	0.00
138	*C1orf123*	2.41	1.73	0.00
139	CDC42EP1	2.40	1.73	0.00
140	*MAGED1*	2.40	1.73	0.00
141	TMEM39B	2.40	1.73	0.00
142	*STYXL1*	2.40	1.73	0.00
143	APLP2	2.40	1.73	0.00
144	*ZNF629*	2.40	1.73	0.00
145	GBAS	2.39	1.73	0.00
146	*RHBDF1*	2.39	1.73	0.00
147	RNASE1	2.39	1.73	0.00
148	*GLS2*	2.39	1.73	0.00
149	ITPR3	2.39	1.73	0.00
150	*FMO4*	2.39	1.73	0.00
151	MEGF6	2.38	1.73	0.00
152	*ADAMTSL2*	2.38	1.72	0.00

153	FHOD1	2.38	1.72	0.00
154	*C7orf42*	2.38	1.72	0.00
155	SPSB3	2.37	1.72	0.00
156	*MIR16*	2.37	1.72	0.00
157	PUM1	2.37	1.72	0.00
158	*CES3*	2.37	1.72	0.00
159	SLC2A4RG	2.37	1.72	0.00
160	*GPR116*	2.37	1.72	0.00
161	SERPIND1	2.37	1.72	0.00
162	*PPCS*	2.36	1.72	0.00
163	RABEP2	2.36	1.72	0.00
164	*GPBP1L1*	2.36	1.72	0.00
165	MRPL20	2.36	1.72	0.00
166	*APOH*	2.35	1.71	0.00
167	RAB11FIP3	2.35	1.71	0.00
168	*SPATA6*	2.35	1.71	0.00
169	PHKB	2.35	1.71	0.00
170	*ORC3L*	2.35	1.71	0.00
171	ZNF219	2.34	1.71	0.00
172	*C16orf42*	2.34	1.71	0.00
173	SLC15A2	2.34	1.71	0.00
174	*KIAA0841*	2.34	1.71	0.00
175	DECR2	2.34	1.71	0.00
176	*CLCN7*	2.33	1.71	0.00
177	TSC2	2.33	1.70	0.00
178	*HPCAL4*	2.33	1.70	0.00
179	MYST1	2.32	1.70	0.00
180	*CREBBP*	2.32	1.70	0.00
181	MVP	2.32	1.70	0.00
182	*GPC4*	2.32	1.70	0.00
183	CADPS2	2.32	1.70	0.00
184	*THRA*	2.32	1.70	0.00
185	FLJ10781	2.31	1.70	0.00
186	*NUBP1*	2.31	1.70	0.00
187	NCDN	2.31	1.70	0.00
188	*SLC9A1*	2.31	1.70	0.00
189	AKR7A2	2.30	1.70	0.00
190	*PKD1*	2.30	1.70	0.00
191	MALL	2.30	1.70	0.00
192	*CDIPT*	2.30	1.70	0.00
193	LCT	2.30	1.69	0.00
194	*PRELP*	2.30	1.69	0.00
195	ROR1	2.30	1.69	0.00
196	*CST5*	2.29	1.69	0.00
197	SF3A3	2.29	1.69	0.00
198	*UBN1*	2.28	1.69	0.00
199	CCT6B	2.28	1.69	0.00
200	*ETV5*	2.28	1.69	0.00
201	FZD1	2.27	1.68	0.00
202	*ALPL*	2.27	1.68	0.00
203	CTSH	2.27	1.68	0.00
204	*DPY19L1*	2.27	1.68	0.00

205	TMEM112	2.27	1.68	0.00
206	*ETNK2*	2.26	1.68	0.00
207	COMP	2.26	1.68	0.00
208	*PCSK1N*	2.26	1.68	0.00
209	GATAD1	2.26	1.68	0.00
210	*UBE2D4*	2.26	1.68	0.00
211	ARMCX6	2.25	1.68	0.00
212	*ABCA4*	2.25	1.68	0.00
213	CEBPA	2.25	1.68	0.00
214	*NARFL*	2.25	1.67	0.00
215	DPP4	2.25	1.67	0.00
216	*CPSF3L*	2.24	1.67	0.00
217	PRSS16	2.24	1.67	0.00
218	*TRAPPC2*	2.24	1.67	0.00
219	RNPS1	2.24	1.67	0.00
220	*ZNF500*	2.23	1.67	0.00
221	SDF4	2.23	1.67	0.00
222	*CLDN4*	2.23	1.67	0.00
223	EXOD1	2.23	1.67	0.00
224	*PER3*	2.23	1.67	0.00
225	LGALS3BP	2.22	1.67	0.00
226	*C16orf35*	2.22	1.66	0.00
227	HMGN2	2.22	1.66	0.00
228	*DDR1*	2.21	1.66	0.00
229	URG4	2.21	1.66	0.00
230	*RCP9*	2.21	1.66	0.00
231	BAZ1B	2.21	1.66	0.00
232	*MYO1D*	2.21	1.66	0.00
233	METRN	2.20	1.66	0.00
234	*BSDC1*	2.20	1.66	0.00
235	FASTK	2.20	1.66	0.00
236	*GDPD5*	2.20	1.66	0.00
237	ATP6V0E2	2.20	1.66	0.00
238	*LIMK1*	2.19	1.66	0.00
239	TAOK2	2.19	1.66	0.00
240	*SFRP4*	2.19	1.66	0.00
241	PDPK1	2.19	1.65	0.00
242	*KIAA1305*	2.19	1.65	0.00
243	CLEC16A	2.19	1.65	0.00
244	*CD207*	2.19	1.65	0.00
245	PHACTR4	2.18	1.65	0.00
246	*AKR1A1*	2.17	1.65	0.00
247	ZNF107	2.17	1.65	0.00
248	*ARSD*	2.17	1.65	0.00
249	DCLK1	2.17	1.65	0.00
250	*APOD*	2.16	1.64	0.00
251	FOLR1	2.16	1.64	0.00
252	*YIPF2*	2.16	1.64	0.00
253	C16orf5	2.15	1.64	0.00
254	*RGL1*	2.15	1.64	0.00
255	POLR2C	2.15	1.64	0.00
256	*C16orf53*	2.15	1.64	0.00

257	RRAD	2.15	1.64	0.00
258	*MAP3K13*	2.15	1.64	0.00
259	ORAI3	2.14	1.64	0.00
260	*HOXD1*	2.14	1.63	0.00
261	CST2	2.14	1.63	0.00
262	*ARID1A*	2.13	1.63	0.00
263	SCP2	2.13	1.63	0.00
264	*BBS9*	2.13	1.63	0.00
265	ARHGEF10L	2.13	1.63	0.00
266	*TOMM7*	2.12	1.63	0.00
267	IPO13	2.12	1.63	0.00
268	*KPNA6*	2.12	1.63	0.00
269	ADCK2	2.12	1.63	0.00
270	*TRIOBP*	2.12	1.63	0.00
271	CLDN3	2.11	1.63	0.00
272	*VKORC1*	2.11	1.63	0.00
273	OSGEP	2.11	1.63	0.00
274	*STUB1*	2.11	1.63	0.00
275	NTHL1	2.10	1.62	0.00
276	*FKBPL*	2.10	1.62	0.00
277	TYRP1	2.10	1.62	0.00
278	*TNNI3*	2.10	1.62	0.00
279	FLJ10357	2.10	1.62	0.00
280	*MPZL2*	2.10	1.62	0.00
281	BCAM	2.09	1.62	0.00
282	*RHOT2*	2.09	1.62	0.00
283	FAM3A	2.09	1.62	0.00
284	*ELOVL1*	2.09	1.62	0.00
285	APITD1	2.09	1.62	0.00
286	*UNC84A*	2.09	1.62	0.00
287	DAAM1	2.08	1.62	0.00
288	*NPAL3*	2.08	1.62	0.00
289	PHKG2	2.08	1.61	0.00
290	*ZNF34*	2.07	1.61	0.00
291	C1orf116	2.07	1.61	0.00
292	*HN1L*	2.07	1.61	0.00
293	ABCC6	2.07	1.61	0.00
294	*SCUBE2*	2.06	1.61	0.00
295	AGRN	2.06	1.61	0.00
296	*FGF18*	2.06	1.61	0.00
297	PHF1	2.06	1.61	0.00
298	*RAC1*	2.06	1.61	0.00
299	CLUAP1	2.06	1.61	0.00
300	*WDR91*	2.06	1.61	0.00
301	PCTK3	2.06	1.61	0.00
302	*ZNF212*	2.05	1.61	0.00
303	COL10A1	2.05	1.61	0.00
304	*TMEM50A*	2.05	1.60	0.00
305	ICMT	2.05	1.60	0.00
306	*STEAP3*	2.05	1.60	0.00
307	TMED9	2.05	1.60	0.00
308	*STK19*	2.05	1.60	0.00

309	ABHD11	2.05	1.60	0.00
310	*RER1*	2.05	1.60	0.00
311	EPB41L1	2.04	1.60	0.00
312	*ZNF764*	2.04	1.60	0.00
313	NINJ2	2.04	1.60	0.00
314	*CD1E*	2.04	1.60	0.00
315	SOX13	2.04	1.60	0.00
316	*C5orf3*	2.04	1.60	0.00
317	GCDH	2.04	1.60	0.00
318	*TCEB3*	2.04	1.60	0.00
319	CD74	2.04	1.60	0.00
320	*BAIAP3*	2.03	1.60	0.00
321	USP7	2.03	1.60	0.00
322	*SCMH1*	2.03	1.60	0.00
323	MOSPD3	2.03	1.60	0.00
324	*MAPK8IP3*	2.03	1.60	0.00
325	MPG	2.03	1.60	0.00
326	*TLR2*	2.03	1.60	0.00
327	ZMYM3	2.03	1.60	0.00
328	*GJB1*	2.03	1.60	0.00
329	CLSTN1	2.03	1.60	0.00
330	*ATP7A*	2.03	1.60	0.00
331	TMEM63A	2.03	1.60	0.00
332	*TMEM112B*	2.02	1.59	0.00
333	KIAA0240	2.02	1.59	0.00
334	*MTCH1*	2.02	1.59	0.00
335	ARHGEF9	2.02	1.59	0.00
336	*PNPLA4*	2.02	1.59	0.00
337	SFRS4	2.02	1.59	0.00
338	*GRB14*	2.02	1.59	0.00
339	SARM1	2.01	1.59	0.00
340	*PLA2G1B*	2.01	1.59	0.00
341	NUDC	2.01	1.59	0.00
342	*PDK2*	2.00	1.59	0.00
343	CLDN9	2.00	1.59	0.00
344	*ACAD8*	2.00	1.59	0.00
345	GFER	2.00	1.59	0.00
346	*CXorf56*	2.00	1.59	0.00
347	CEACAM4	2.00	1.59	0.00
348	*RAPGEF5*	2.00	1.58	0.00
349	MRPS18B	1.99	1.58	0.00
350	*ZNF354A*	1.99	1.58	0.00
351	C19orf56	1.99	1.58	0.00
352	*C18orf1*	1.99	1.58	0.00
353	POU2F3	1.99	1.58	0.00
354	*PRSS8*	1.99	1.58	0.00
355	SGSH	1.99	1.58	0.00
356	*WWP2*	1.99	1.58	0.00
357	WBSCR22	1.99	1.58	0.00
358	*TRIM26*	1.99	1.58	0.00
359	AEBP1	1.99	1.58	0.00
360	*CUEDC1*	1.99	1.58	0.00

361	THOC6	1.98	1.58	0.00
362	*MGP*	1.98	1.58	0.00
363	TCFL5	1.98	1.58	0.13
364	*REV1*	1.98	1.58	0.13
365	KIAA0467	1.98	1.58	0.13
366	*ITGA9*	1.98	1.58	0.13
367	UBTD1	1.98	1.58	0.13
368	*AHR*	1.98	1.58	0.13
369	PPP1R3C	1.98	1.58	0.13
370	*GALNT11*	1.97	1.58	0.13
371	PGRMC1	1.97	1.58	0.13
372	*WDR13*	1.97	1.58	0.13
373	IPP	1.97	1.58	0.13
374	*DOK5*	1.97	1.57	0.13
375	RRN3	1.96	1.57	0.13
376	*ALG12*	1.96	1.57	0.13
377	HLA-DPB1	1.96	1.57	0.13
378	*HSPB7*	1.96	1.57	0.13
379	CDH1	1.96	1.57	0.13
380	*MUTYH*	1.96	1.57	0.13
381	F11	1.96	1.57	0.13
382	*HLA-DPA1*	1.95	1.57	0.13
383	UXS1	1.95	1.57	0.13
384	*ZFHX3*	1.95	1.57	0.13
385	LOC81691	1.95	1.57	0.13
386	*AMOT*	1.95	1.57	0.13
387	TP53AP1	1.95	1.57	0.13
388	*CALCOCO2*	1.95	1.57	0.13
389	AGPAT1	1.94	1.57	0.13
390	*C1orf50*	1.94	1.57	0.13
391	FLJ22222	1.94	1.57	0.13
392	*GPR137*	1.94	1.57	0.13
393	IL27RA	1.94	1.56	0.13
394	*CASD1*	1.94	1.56	0.13
395	NOD1	1.93	1.56	0.13
396	*VPS41*	1.93	1.56	0.13
397	FBXO42	1.93	1.56	0.13
398	*ST7*	1.93	1.56	0.13
399	COL8A2	1.93	1.56	0.13
400	*GSPT1*	1.92	1.56	0.13
401	HAND1	1.92	1.56	0.13
402	*ZNF12*	1.92	1.56	0.13
403	GGCX	1.92	1.56	0.13
404	*C7orf26*	1.92	1.56	0.13
405	GBL	1.92	1.56	0.13
406	*SPINT2*	1.92	1.56	0.13
407	CNNM3	1.92	1.56	0.13
408	*ZMYM6*	1.92	1.56	0.13
409	ECHDC2	1.92	1.56	0.13
410	*HSD11B2*	1.91	1.56	0.13
411	SKIV2L	1.91	1.56	0.13
412	*NUPL2*	1.91	1.56	0.13

413	STK17A	1.91	1.55	0.13
414	*ENC1*	1.91	1.55	0.13
415	AZGP1	1.91	1.55	0.13
416	*LRRC20*	1.91	1.55	0.13
417	SLC13A3	1.91	1.55	0.13
418	*SH3BGR*	1.91	1.55	0.13
419	IFI6	1.91	1.55	0.13
420	*ARAF*	1.90	1.55	0.13
421	USP11	1.90	1.55	0.13
422	*GRAMD3*	1.90	1.55	0.13
423	CLN3	1.89	1.55	0.13
424	*TMEM186*	1.89	1.55	0.13
425	RABL4	1.89	1.55	0.13
426	*DIO1*	1.89	1.55	0.13
427	SULT1C2	1.89	1.55	0.13
428	*WIPI2*	1.89	1.55	0.13
429	UBE2I	1.89	1.55	0.30
430	*PARN*	1.88	1.55	0.30
431	CRYM	1.88	1.55	0.30
432	*TBC1D10B*	1.88	1.54	0.30
433	ACTB	1.88	1.54	0.30
434	*MFN2*	1.88	1.54	0.30
435	GGT1	1.88	1.54	0.30
436	*MGC10334*	1.88	1.54	0.30
437	TBL2	1.88	1.54	0.30
438	*TXLNA*	1.88	1.54	0.30
439	BSCL2	1.88	1.54	0.30
440	*RASGRF1*	1.88	1.54	0.30
441	C7orf10	1.87	1.54	0.30
442	*SLC2A10*	1.87	1.54	0.30
443	FCGBP	1.87	1.54	0.30
444	*TCEB2*	1.87	1.54	0.30
445	C20orf103	1.87	1.54	0.30
446	*DAP*	1.87	1.54	0.30
447	EIF2C1	1.87	1.54	0.30
448	*RAP1GAP*	1.87	1.54	0.30
449	PIB5PA	1.86	1.54	0.30
450	*WBSCR16*	1.86	1.54	0.30
451	DOC2A	1.86	1.54	0.30
452	*RNF216*	1.86	1.54	0.30
453	LTBP1	1.86	1.54	0.30
454	*RORB*	1.86	1.54	0.30
455	PPT1	1.86	1.54	0.30
456	*ZNF434*	1.86	1.54	0.30
457	ARHGDIB	1.85	1.53	0.30
458	*FAF1*	1.85	1.53	0.30
459	SUSD4	1.85	1.53	0.30
460	*AP4S1*	1.85	1.53	0.30
461	PPL	1.85	1.53	0.30
462	*MYOZ1*	1.85	1.53	0.30
463	DNAJA3	1.85	1.53	0.30
464	*RIMS3*	1.84	1.53	0.30

465	NOTCH2	1.84	1.53	0.30
466	*CYB5R1*	1.84	1.53	0.30
467	MRPS34	1.84	1.53	0.30
468	*RING1*	1.84	1.53	0.30
469	TLE2	1.84	1.53	0.30
470	*CD1A*	1.84	1.53	0.30
471	DLK1	1.84	1.53	0.30
472	*THTPA*	1.83	1.53	0.30
473	B3GNT1	1.83	1.53	0.30
474	*SHROOM2*	1.83	1.53	0.30
475	KIAA1026	1.83	1.53	0.30
476	*SIRT3*	1.83	1.53	0.30
477	WFDC2	1.82	1.53	0.30
478	*PINK1*	1.82	1.52	0.30
479	TRIM24	1.82	1.52	0.30
480	*CD82*	1.82	1.52	0.30
481	BBS1	1.82	1.52	0.30
482	*HHLA2*	1.82	1.52	0.30
483	PLXNA2	1.82	1.52	0.30
484	*BTG3*	1.82	1.52	0.30
485	MED8	1.82	1.52	0.30
486	*WDR8*	1.81	1.52	0.30
487	MAGED2	1.81	1.52	0.30
488	*NSUN5C*	1.81	1.52	0.30
489	ABCB8	1.81	1.52	0.30
490	*BNIP1*	1.81	1.52	0.30
491	DHX58	1.81	1.52	0.30
492	*HNF1B*	1.81	1.52	0.30
493	PTPRF	1.81	1.52	0.30
494	*KRIT1*	1.81	1.52	0.30
495	S100A13	1.80	1.52	0.30
496	*CDR2*	1.80	1.52	0.30
497	JUN	1.80	1.52	0.40
498	*FLJ20920*	1.80	1.52	0.40
499	PPP1R9A	1.80	1.52	0.40
500	*C6orf47*	1.80	1.52	0.40
501	PRKD1	1.80	1.52	0.40
502	*GPC3*	1.80	1.52	0.40
503	WWC1	1.80	1.52	0.40
504	*ATAD4*	1.80	1.52	0.40
505	SYNPO	1.79	1.52	0.40
506	*RAB38*	1.79	1.51	0.40
507	IFNGR2	1.79	1.51	0.40
508	*MYH2*	1.79	1.51	0.40
509	FBXW11	1.79	1.51	0.40
510	*LBA1*	1.79	1.51	0.40
511	USP13	1.79	1.51	0.40
512	*CAPZB*	1.79	1.51	0.40
513	FLJ20323	1.79	1.51	0.40
514	*PEX14*	1.78	1.51	0.40
515	TAX1BP1	1.78	1.51	0.40
516	*CXCL14*	1.78	1.51	0.40

517	EMP2	1.78	1.51	0.40
518	*DNAJC4*	1.78	1.51	0.40
519	C7orf24	1.78	1.51	0.40
520	*SLC12A9*	1.78	1.51	0.40
521	NDUFB2	1.78	1.51	0.40
522	*THYN1*	1.77	1.51	0.40
523	ZNF768	1.77	1.51	0.40
524	*PDGFD*	1.77	1.51	0.40
525	SYT17	1.77	1.51	0.40
526	*HLA-DRA*	1.77	1.51	0.40
527	NKX2-1	1.77	1.51	0.40
528	*NUMA1*	1.77	1.51	0.40
529	UBB	1.76	1.51	0.40
530	*PRODH*	1.76	1.50	0.40
531	PXMP4	1.76	1.50	0.40
532	*EXPH5*	1.76	1.50	0.40
533	AURKAIP1	1.76	1.50	0.40
534	*TCEAL4*	1.76	1.50	0.40
535	E4F1	1.76	1.50	0.40
536	*MICALL2*	1.76	1.50	0.40
537	KIAA0247	1.76	1.50	0.40
538	*BEX4*	1.76	1.50	0.40
539	SLC1A7	1.75	1.50	0.40
540	*MXRA8*	1.75	1.50	0.40
541	ZNF174	1.75	1.50	0.40
542	*HLA-DMB*	1.75	1.50	0.40
543	ZNF673	1.75	1.50	0.40
544	*GFRA3*	1.75	1.50	0.40
545	CSDC2	1.75	1.50	0.40
546	*EIF4H*	1.75	1.50	0.40
547	CNKSR1	1.75	1.50	0.40
548	*HSPG2*	1.75	1.50	0.40
549	TRADD	1.75	1.50	0.40
550	*BGN*	1.74	1.50	0.40
551	PDE9A	1.74	1.50	0.40
552	*HLA-DOA*	1.74	1.50	0.40
553	TACSTD2	1.74	1.50	0.40
554	*XAB2*	1.74	1.50	0.40
555	SRCAP	1.74	1.50	0.40
556	*PEX3*	1.74	1.50	0.40
557	ZNF702	1.74	1.50	0.40
558	*FOXJ3*	1.74	1.50	0.40
559	CROCC	1.73	1.49	0.40
560	*DHDDS*	1.73	1.49	0.40
561	LRRC36	1.73	1.49	0.40
562	*GDF11*	1.73	1.49	0.40
563	F8A1	1.73	1.49	0.40
564	*CBX6*	1.73	1.49	0.40
565	C1orf66	1.72	1.49	0.40
566	*NELL1*	1.72	1.49	0.40
567	PSMC2	1.72	1.49	0.40
568	*LOC89944*	1.72	1.49	0.40

569	SRRM1	1.72	1.49	0.40
570	*MBIP*	1.72	1.49	0.40
571	PKD2	1.72	1.49	0.40
572	*NASP*	1.72	1.49	0.40
573	VTCN1	1.72	1.49	0.66
574	*MMP28*	1.71	1.49	0.66
575	C11orf61	1.71	1.49	0.66
576	*NRIP3*	1.71	1.49	0.66
577	ADORA1	1.71	1.49	0.66
578	*ABCD3*	1.71	1.49	0.66
579	TRPM4	1.71	1.49	0.66
580	*CETN2*	1.71	1.49	0.66
581	NPTX2	1.71	1.49	0.66
582	*SNX13*	1.71	1.49	0.66
583	AP2A2	1.71	1.49	0.66
584	*C6orf60*	1.71	1.49	0.66
585	PIK3R3	1.70	1.48	0.66
586	*ZNF302*	1.70	1.48	0.66
587	WDR42A	1.70	1.48	0.66
588	*AP1M2*	1.70	1.48	0.66
589	CCDC121	1.70	1.48	0.66
590	*TNFSF10*	1.70	1.48	0.66
591	VPS52	1.70	1.48	0.66
592	*TRIM68*	1.70	1.48	0.66
593	GNAS	1.69	1.48	0.66
594	*CNNM1*	1.69	1.48	0.66
595	ECM1	1.69	1.48	0.66
596	*ROS1*	1.69	1.48	0.66
597	DAAM2	1.69	1.48	0.66
598	*TNFRSF12A*	1.69	1.48	0.66
599	PFKL	1.69	1.48	0.66
600	*EN2*	1.69	1.48	0.66
601	ANPEP	1.69	1.48	0.66
602	*CHST12*	1.69	1.48	0.66
603	FGF13	1.69	1.48	0.66
604	*C20orf149*	1.69	1.48	0.66
605	FLJ23861	1.68	1.48	0.66
606	*FAM90A1*	1.68	1.48	0.66
607	SSPN	1.68	1.48	0.66
608	*ABR*	1.68	1.48	0.66
609	ROM1	1.68	1.48	0.66
610	*ATPBD1B*	1.68	1.48	0.66
611	CRYZ	1.68	1.48	0.66
612	*C1orf109*	1.68	1.48	0.66
613	CD1C	1.68	1.48	0.66
614	*LAPTM4A*	1.68	1.48	0.66
615	CRABP2	1.68	1.48	0.66
616	*IFI44*	1.68	1.48	0.66
617	DKK3	1.68	1.48	0.66
618	*RBM23*	1.68	1.48	0.66
619	GCAT	1.68	1.48	0.66
620	*RANBP17*	1.67	1.47	0.66

621	RBM9	1.67	1.47	0.66
622	*BCORL1*	1.67	1.47	0.66
623	MDK	1.67	1.47	0.66
624	*SPRED2*	1.67	1.47	0.66
625	DOK4	1.67	1.47	0.66
626	*LSP1*	1.67	1.47	0.66
627	TUFM	1.67	1.47	0.66
628	*PGCP*	1.66	1.47	0.66
629	FOXRED2	1.66	1.47	0.66
630	*C11orf68*	1.66	1.47	0.66
631	IDS	1.66	1.47	0.66
632	*C9orf127*	1.66	1.47	0.66
633	FAM134C	1.66	1.47	0.66
634	*TNFSF15*	1.66	1.47	0.66
635	SLC17A3	1.66	1.47	0.66
636	*DEXI*	1.66	1.47	0.66
637	ST3GAL6	1.66	1.47	0.66
638	*HLA-A*	1.66	1.47	0.66
639	HOXC4	1.66	1.47	0.66
640	*MYH10*	1.66	1.47	0.66
641	CAPN2	1.66	1.47	0.66
642	*PIK3IP1*	1.66	1.47	0.66
643	TMEM8	1.66	1.47	0.66
644	*NPHP4*	1.65	1.47	0.66
645	KIAA0644	1.65	1.47	0.66
646	*ICAM1*	1.65	1.47	0.66
647	SPOP	1.65	1.47	0.66
648	*NFIX*	1.65	1.47	0.66
649	PHC2	1.65	1.47	0.66
650	*KIF22*	1.65	1.47	0.66
651	C14orf133	1.65	1.47	0.66
652	*MACROD1*	1.65	1.47	0.66
653	B3GAT1	1.65	1.47	0.66
654	*KIAA0082*	1.65	1.47	0.66
655	SIDT1	1.65	1.47	0.66
656	*TMEM59*	1.65	1.47	0.66
657	RPS6KA3	1.64	1.47	0.66
658	*ZNF43*	1.64	1.46	0.66
659	ITGBL1	1.64	1.46	0.66
660	*IL10RB*	1.64	1.46	0.66
661	SLC4A5	1.64	1.46	0.66
662	*KIAA0562*	1.64	1.46	0.66
663	TSPAN4	1.64	1.46	0.66
664	*SELENBP1*	1.64	1.46	0.66
665	MATN3	1.64	1.46	0.66
666	*SLC35E1*	1.64	1.46	0.66
667	FABP3	1.64	1.46	0.66
668	*POMGNT1*	1.64	1.46	0.66
669	ZNF137	1.64	1.46	0.66
670	*TMPRSS2*	1.64	1.46	0.66
671	AK1	1.64	1.46	0.66
672	*ADAT1*	1.64	1.46	0.66

673	SPRY1	1.64	1.46	0.66
674	*CHCHD2*	1.64	1.46	0.66
675	AKT1	1.64	1.46	0.66
676	*NDUFA6*	1.64	1.46	0.66
677	SYNJ2BP	1.64	1.46	0.66
678	*FAM129A*	1.63	1.46	0.66
679	ZNF76	1.63	1.46	0.66
680	*NEU1*	1.63	1.46	0.66
681	TSPAN9	1.63	1.46	0.66
682	*CPM*	1.63	1.46	0.66
683	ETV4	1.63	1.46	0.66
684	*NLRX1*	1.63	1.46	0.66
685	BZRAP1	1.63	1.46	0.66
686	*INHBB*	1.63	1.46	0.66
687	RAMP1	1.63	1.46	0.66
688	*KRCC1*	1.62	1.46	0.66
689	DHRS3	1.62	1.46	0.66
690	*RXRB*	1.62	1.46	0.66
691	PRDM4	-3.57	0.45	0.00
692	*GTF2E2*	-3.39	0.47	0.00
693	TXNRD1	-3.11	0.50	0.00
694	*DUSP4*	-3.08	0.50	0.00
695	LEPROTL1	-2.98	0.51	0.00
696	*SNF1LK*	-2.94	0.52	0.00
697	DDX21	-2.92	0.52	0.00
698	*KCTD9*	-2.88	0.52	0.00
699	TNFRSF10B	-2.83	0.53	0.00
700	*CHMP7*	-2.81	0.53	0.00
701	PPP2R2A	-2.81	0.53	0.00
702	*PAPD1*	-2.78	0.53	0.00
703	ZCCHC2	-2.76	0.54	0.00
704	*RC3H2*	-2.74	0.54	0.00
705	DCTN6	-2.73	0.54	0.00
706	*PPP1CC*	-2.72	0.54	0.00
707	KIAA1033	-2.70	0.54	0.00
708	*RPL7A*	-2.69	0.54	0.00
709	RPLP0	-2.68	0.54	0.00
710	*ID1*	-2.67	0.54	0.00
711	ENTPD4	-2.63	0.55	0.00
712	*GCH1*	-2.61	0.55	0.00
713	RHOQ	-2.61	0.55	0.00
714	*STK24*	-2.60	0.55	0.00
715	HRB	-2.55	0.56	0.00
716	*DYNC1LI1*	-2.54	0.56	0.00
717	KIAA0020	-2.54	0.56	0.00
718	*PTCD3*	-2.54	0.56	0.00
719	MEMO1	-2.54	0.56	0.00
720	*LSM1*	-2.54	0.56	0.00
721	WRN	-2.54	0.56	0.00
722	*TDG*	-2.53	0.56	0.00
723	ERLIN1	-2.49	0.57	0.00
724	*UBE2E1*	-2.47	0.57	0.00

725	AGPAT5	-2.46	0.57	0.00
726	*GNL3*	-2.46	0.57	0.00
727	ARPP-19	-2.46	0.57	0.00
728	*STAM*	-2.44	0.57	0.00
729	CYB5R4	-2.43	0.57	0.00
730	*EIF3A*	-2.43	0.57	0.00
731	ORC2L	-2.41	0.58	0.00
732	*ISG20*	-2.41	0.58	0.00
733	FGG	-2.40	0.58	0.00
734	*BAG1*	-2.40	0.58	0.00
735	CHUK	-2.39	0.58	0.00
736	*PDSS1*	-2.39	0.58	0.00
737	INTS6	-2.39	0.58	0.00
738	*RAB35*	-2.39	0.58	0.00
739	RPS6	-2.36	0.58	0.00
740	*CNOT7*	-2.35	0.58	0.00
741	PTP4A1	-2.34	0.59	0.00
742	*C8orf41*	-2.34	0.59	0.00
743	UBXD6	-2.32	0.59	0.00
744	*URM1*	-2.32	0.59	0.00
745	MUC4	-2.32	0.59	0.00
746	*IRS2*	-2.31	0.59	0.00
747	MRPS2	-2.31	0.59	0.00
748	*RFK*	-2.30	0.59	0.00
749	NOLA3	-2.29	0.59	0.00
750	*TEX10*	-2.29	0.59	0.00
751	SLC39A14	-2.28	0.59	0.00
752	*KIF5B*	-2.28	0.59	0.00
753	EIF3J	-2.27	0.59	0.00
754	*DDHD2*	-2.26	0.60	0.00
755	EIF4A1	-2.26	0.60	0.00
756	*AVPI1*	-2.25	0.60	0.00
757	DNM1L	-2.25	0.60	0.13
758	*NR4A2*	-2.24	0.60	0.13
759	ARHGEF10	-2.22	0.60	0.13
760	*FBXL2*	-2.22	0.60	0.13
761	SEC61B	-2.22	0.60	0.13
762	*SHOC2*	-2.22	0.60	0.13
763	SOCS6	-2.21	0.60	0.13
764	*UTP3*	-2.21	0.60	0.13
765	RBM13	-2.21	0.60	0.13
766	*POLR1D*	-2.21	0.60	0.13
767	KIAA0368	-2.21	0.60	0.13
768	*RANBP5*	-2.21	0.60	0.13
769	CHMP1B	-2.20	0.60	0.13
770	*SNRPD1*	-2.20	0.60	0.13
771	KLHL9	-2.20	0.60	0.13
772	*C18orf8*	-2.20	0.60	0.13
773	ACTR3	-2.20	0.60	0.13
774	*MYC*	-2.18	0.61	0.13
775	KRAS	-2.18	0.61	0.13
776	*ADAM10*	-2.17	0.61	0.13

777	MAPKAPK5	-2.17	0.61	0.13
778	*PSMA4*	-2.17	0.61	0.13
779	SLC7A11	-2.17	0.61	0.13
780	*GOLGA7*	-2.16	0.61	0.13
781	PPIF	-2.16	0.61	0.13
782	*SETX*	-2.16	0.61	0.13
783	KCMF1	-2.16	0.61	0.13
784	*NARS*	-2.15	0.61	0.13
785	RAP2A	-2.15	0.61	0.13
786	*GPX2*	-2.15	0.61	0.13
787	SRP72	-2.14	0.61	0.13
788	*CTSB*	-2.14	0.61	0.13
789	TTLL4	-2.14	0.61	0.13
790	*PIK3C3*	-2.14	0.61	0.13
791	RPL35	-2.13	0.61	0.13
792	*KPNA3*	-2.13	0.61	0.13
793	BIN3	-2.13	0.61	0.13
794	*AGTPBP1*	-2.13	0.61	0.30
795	PTPN11	-2.13	0.61	0.30
796	*UBAC1*	-2.13	0.61	0.30
797	GSR	-2.12	0.61	0.30
798	*EHBP1*	-2.12	0.61	0.30
799	RPL29	-2.12	0.61	0.30
800	*MTHFD2*	-2.12	0.61	0.30
801	SOD2	-2.12	0.61	0.30
802	*SET*	-2.12	0.61	0.30
803	HMOX1	-2.11	0.62	0.30
804	*VDAC3*	-2.10	0.62	0.30
805	VDAC2	-2.10	0.62	0.30
806	*ZMYM2*	-2.10	0.62	0.30
807	GABPB2	-2.09	0.62	0.30
808	*RPL6*	-2.09	0.62	0.30
809	MEIS2	-2.09	0.62	0.30
810	*PPP3CC*	-2.09	0.62	0.30
811	SMAD2	-2.08	0.62	0.30
812	*COPS2*	-2.08	0.62	0.30
813	TMF1	-2.08	0.62	0.30
814	*TXNL4A*	-2.07	0.62	0.30
815	XPO7	-2.07	0.62	0.30
816	*PPP2R1B*	-2.07	0.62	0.30
817	ODC1	-2.07	0.62	0.30
818	*SCARB1*	-2.06	0.62	0.30
819	MRPS35	-2.06	0.62	0.30
820	*POLR3D*	-2.06	0.62	0.30
821	NDUFV2	-2.06	0.62	0.30
822	*HYPK*	-2.06	0.62	0.30
823	IKBKAP	-2.06	0.62	0.30
824	*STX2*	-2.05	0.62	0.30
825	CHD7	-2.05	0.62	0.30
826	*MAPK6*	-2.05	0.62	0.30
827	RAN	-2.05	0.62	0.30
828	*MFHAS1*	-2.05	0.62	0.30

829	PDE4D	-2.05	0.62	0.30
830	*RPS17*	-2.04	0.62	0.30
831	GTPBP4	-2.04	0.62	0.30
832	*PWP1*	-2.04	0.62	0.30
833	RCBTB1	-2.04	0.63	0.30
834	*ITGB1*	-2.03	0.63	0.30
835	KIAA0157	-2.03	0.63	0.30
836	*SLC16A3*	-2.03	0.63	0.30
837	GSTO1	-2.03	0.63	0.30
838	*PDHB*	-2.03	0.63	0.30
839	KYNU	-2.03	0.63	0.30
840	*CTDP1*	-2.02	0.63	0.30
841	VDAC1	-2.02	0.63	0.30
842	*GALK2*	-2.02	0.63	0.30
843	PLEKHJ1	-2.02	0.63	0.30
844	*C8orf4*	-2.02	0.63	0.30
845	C4orf16	-2.02	0.63	0.30
846	*TMEM93*	-2.02	0.63	0.30
847	PUS1	-2.02	0.63	0.30
848	*VPS33B*	-2.02	0.63	0.30
849	UCK2	-2.02	0.63	0.30
850	*PLAUR*	-2.02	0.63	0.30
851	CEBPB	-2.01	0.63	0.30
852	*CCDC59*	-2.01	0.63	0.30
853	UBE2N	-2.01	0.63	0.30
854	*SGCB*	-2.01	0.63	0.30
855	PPP6C	-2.00	0.63	0.30
856	*VAPA*	-2.00	0.63	0.30
857	RTF1	-2.00	0.63	0.30
858	*FAM60A*	-2.00	0.63	0.30
859	C10orf22	-2.00	0.63	0.30
860	*RPS7*	-2.00	0.63	0.30
861	RPS3	-2.00	0.63	0.30
862	*PLAA*	-2.00	0.63	0.30
863	ATF4	-1.99	0.63	0.30
864	*PPP1R12A*	-1.99	0.63	0.30
865	RY1	-1.99	0.63	0.30
866	*MTMR9*	-1.99	0.63	0.30
867	YME1L1	-1.99	0.63	0.30
868	*GTF3A*	-1.99	0.63	0.30
869	C5orf30	-1.98	0.63	0.30
870	*PMPCA*	-1.98	0.63	0.30
871	TATDN2	-1.98	0.63	0.30
872	*NRBF2*	-1.98	0.63	0.40
873	WIPI1	-1.98	0.63	0.40
874	*CLDND1*	-1.98	0.63	0.40
875	SLC25A37	-1.98	0.63	0.40
876	*HN1*	-1.97	0.63	0.40
877	FECH	-1.97	0.64	0.40
878	*CTSL1*	-1.96	0.64	0.40
879	C12orf5	-1.96	0.64	0.40
880	*FZD3*	-1.96	0.64	0.40

881	KCTD3	-1.96	0.64	0.40
882	*TNFRSF1A*	-1.96	0.64	0.40
883	MRPL42	-1.96	0.64	0.40
884	*MBD2*	-1.96	0.64	0.40
885	SPCS3	-1.96	0.64	0.40
886	*RPS19*	-1.96	0.64	0.40
887	RNF34	-1.95	0.64	0.40
888	*S100P*	-1.95	0.64	0.40
889	CYP24A1	-1.95	0.64	0.40
890	*GZMB*	-1.95	0.64	0.40
891	PPP4R2	-1.95	0.64	0.40
892	*C12orf29*	-1.94	0.64	0.40
893	CLPX	-1.94	0.64	0.40
894	*ACTR6*	-1.94	0.64	0.40
895	PFKP	-1.94	0.64	0.40
896	*ASH2L*	-1.94	0.64	0.40
897	EMR2	-1.94	0.64	0.40
898	*CCT7*	-1.94	0.64	0.40
899	CUL2	-1.94	0.64	0.40
900	*GLCE*	-1.94	0.64	0.40
901	AVEN	-1.93	0.64	0.40
902	*SART3*	-1.93	0.64	0.40
903	WASF1	-1.93	0.64	0.40
904	*RPL13A*	-1.93	0.64	0.40
905	KIAA0701	-1.93	0.64	0.40
906	*C15orf15*	-1.93	0.64	0.40
907	ROD1	-1.93	0.64	0.40
908	*LTF*	-1.92	0.64	0.40
909	ETNK1	-1.92	0.64	0.40
910	*GCN1L1*	-1.92	0.64	0.40
911	PYROXD1	-1.92	0.64	0.40
912	*WSB2*	-1.92	0.64	0.40
913	GRB2	-1.92	0.64	0.40
914	*RPS6KB1*	-1.92	0.64	0.40
915	USP15	-1.91	0.64	0.40
916	*STOML2*	-1.91	0.64	0.40
917	KIAA1012	-1.91	0.64	0.40
918	*PTS*	-1.91	0.64	0.40
919	RIOK3	-1.91	0.64	0.40
920	*PDCD6IP*	-1.91	0.64	0.40
921	C19orf21	-1.90	0.64	0.40
922	*MAFF*	-1.90	0.64	0.40
923	TRIM32	-1.90	0.64	0.40
924	*ANKRD12*	-1.90	0.64	0.40
925	FAM20B	-1.90	0.64	0.40
926	*CHST11*	-1.90	0.65	0.40
927	ATP6V0A2	-1.90	0.65	0.40
928	*HERC2*	-1.89	0.65	0.40
929	SERPINB8	-1.89	0.65	0.40
930	*TRIP12*	-1.89	0.65	0.40
931	GTF2A2	-1.89	0.65	0.40
932	*SS18L2*	-1.88	0.65	0.40

933	PITRM1	-1.88	0.65	0.40
934	*TMEM176A*	-1.88	0.65	0.40
935	CAMSAP1	-1.88	0.65	0.40
936	*MED27*	-1.88	0.65	0.40
937	PARD3	-1.88	0.65	0.40
938	*PBK*	-1.88	0.65	0.40
939	PLUNC	-1.87	0.65	0.40
940	*NUP88*	-1.87	0.65	0.40
941	IL8	-1.87	0.65	0.40
942	*ZCCHC6*	-1.86	0.65	0.40
943	TXNL1	-1.86	0.65	0.40
944	*POMP*	-1.86	0.65	0.40
945	MAK10	-1.85	0.65	0.40
946	*IL15RA*	-1.85	0.65	0.40
947	MTMR2	-1.85	0.65	0.40
948	*DDX54*	-1.85	0.65	0.40
949	XPNPEP1	-1.85	0.65	0.66
950	*ECT2*	-1.85	0.65	0.66
951	CXCL2	-1.85	0.65	0.66
952	*CDV3*	-1.85	0.65	0.66
953	BASP1	-1.85	0.65	0.66
954	*CHML*	-1.85	0.65	0.66
955	NOL8	-1.85	0.65	0.66
956	*SLC7A1*	-1.85	0.65	0.66
957	LRFN4	-1.84	0.65	0.66
958	*ISG20L1*	-1.84	0.65	0.66
959	HIP2	-1.84	0.65	0.66
960	*IPPK*	-1.84	0.65	0.66
961	RSRC2	-1.84	0.65	0.66
962	*PCM1*	-1.84	0.65	0.66
963	RARRES1	-1.84	0.65	0.66
964	*FBXO21*	-1.84	0.65	0.66
965	APPL2	-1.84	0.65	0.66
966	*SCD*	-1.83	0.65	0.66
967	COX5A	-1.83	0.65	0.66
968	*TP53BP1*	-1.83	0.65	0.66
969	INTS7	-1.83	0.65	0.66
970	*EDG2*	-1.83	0.65	0.66
971	UBE1C	-1.83	0.65	0.66
972	*ZC3H15*	-1.83	0.65	0.66
973	C14orf161	-1.83	0.66	0.66
974	*CHFR*	-1.82	0.66	0.66
975	BUB1B	-1.82	0.66	0.66
976	*OXSR1*	-1.82	0.66	0.66
977	NAB1	-1.82	0.66	0.66
978	*AGPS*	-1.82	0.66	0.66
979	TIMM17A	-1.82	0.66	0.66
980	*TNFSF5IP1*	-1.82	0.66	0.66
981	PRMT1	-1.82	0.66	0.66
982	*TEAD4*	-1.81	0.66	0.66
983	CDK7	-1.81	0.66	0.66
984	*C19orf10*	-1.81	0.66	0.66

985	USP34	-1.81	0.66	0.66
986	*PRPF4*	-1.81	0.66	0.66
987	IARS	-1.81	0.66	0.66
988	*PSMD9*	-1.81	0.66	0.66
989	GNLY	-1.81	0.66	0.66
990	*FBXW2*	-1.80	0.66	0.66
991	UGDH	-1.80	0.66	0.66
992	*VCP*	-1.80	0.66	0.66
993	PRPF40A	-1.80	0.66	0.66
994	*ADAMDEC1*	-1.80	0.66	0.66
995	P15RS	-1.80	0.66	0.66
996	*ARL4C*	-1.80	0.66	0.66
997	SEC16A	-1.79	0.66	0.66
998	*CSDA*	-1.79	0.66	0.66
999	PAK2	-1.79	0.66	0.66
1000	*RIC8B*	-1.79	0.66	0.66
1001	TMEM176B	-1.78	0.66	0.66
1002	*STK4*	-1.78	0.66	0.66
1003	AGPAT7	-1.78	0.66	0.66
1004	*IVNS1ABP*	-1.78	0.66	0.66
1005	MED13L	-1.78	0.66	0.66
1006	*BNIP2*	-1.77	0.66	0.66
1007	ENDOG	-1.77	0.66	0.66
1008	*PDCL*	-1.77	0.66	0.66
1009	MBD1	-1.77	0.66	0.66
1010	*YARS2*	-1.77	0.66	0.66
1011	P2RX5	-1.77	0.66	0.66
1012	*RABEPK*	-1.77	0.66	0.66
1013	CLN8	-1.77	0.66	0.66
1014	*ENTPD7*	-1.76	0.66	0.66
1015	RPS27A	-1.76	0.66	0.66
1016	*SPATS2*	-1.76	0.67	0.66
1017	INTS9	-1.76	0.67	0.66
1018	*KLHL18*	-1.76	0.67	0.66
1019	F3	-1.76	0.67	0.66
1020	*JMJD6*	-1.76	0.67	0.66

Supplemental Table S2. Performance test results between EGFR-like mutant group and wild-type group based on *EGFR* -related gene signature. mt, mutant, WT, wild-type, PPV, positive predictive value, NPV, negative predictive value.

Dataset	Cohort	EGFR mSig	EGFR Status		Total	Performance				
			mt	WT		Sensitivity	Specificity	Accuracy	PPV	NPV
Training	MSKCC	+	35	25	60	0.90	0.84	0.85	0.58	0.97
		-	4	128	132					
		Total	39	153	192					
Validation	UNC+TSP	+	13	10	23	0.72	0.90	0.87	0.57	0.95
		-	5	86	91					
		Total	18	96	114					
Validation	TCGA	+	52	54	106	0.81	0.87	0.86	0.49	0.97
		-	12	368	380					
		Total	64	422	486					
Average						0.81	0.87	0.86	0.55	0.96

Supplemental Table S3. Association between mSig status and histological subtypes. Detailed histology were grouped by previously defined predominant growth pattern (PMID: 25079552). P-value was calculated by Fisher's Exact Test.

Histology Subtype	Histology Description	Total	mSig(+)	mSig(-)	Odd Ratio	CI95_low	CI95_high	P-value
Lepidic	Lepidic adenocarcinoma and bronchioloalveolar carcinoma (non-mucinous)	21	7	14	1.85	0.61	5.05	0.19
Papillary	Papillary adenocarcinoma	21	7	14	1.85	0.61	5.05	0.19
Acinar	Acinar cell carcinoma	23	7	16	1.61	0.54	4.27	0.31
Mucinous	Invasive and mucinous adenocarcinoma	16	2	14	0.50	0.05	2.25	0.54
Solid	Solid carcinoma	5	0	5	0.00	0.00	3.92	0.59
Others	Adenocarcinoma, not otherwise specified, adenocarcinoma with mixed subtypes, signet ring cell carcinoma, clear cell carcinoma	400	83	317	0.72	0.41	1.29	0.25

Supplemental Table S4. Odds Ratio and p-value; each subtype vs. all others.

Odds Ratio	EGFR mt	EGFR mSig	EGFR cn	TP53 mt	KEAP1/STK11 mt	RAS/RAF/RTK mt	EGFR ge	p16/CDKN2A ge	NKX2-1 ge	TP63 ge	DUSP4 ge
Bronchioid	4.48	9.18	0.50	0.26	0.35	1.44	1.46	0.71	4.08	2.73	0.43
Magnoid	0.17	0.09	0.91	0.89	8.09	0.90	0.31	0.66	0.87	0.20	5.52
Squamoid	0.59	0.39	2.23	4.65	0.32	0.77	1.94	2.09	0.28	1.44	0.56
EGFR mt	NA	29.18	6.89	0.97	0.07	0.01	8.76	0.93	1.55	1.80	0.05
EGFR mSig	NA	NA	2.45	0.83	0.11	0.43	4.08	1.16	3.84	1.99	0.08
EGFR cn	NA	NA	NA	2.67	0.60	0.45	3.10	1.37	0.62	1.28	0.49
TP53 mt	NA	NA	NA	NA	0.66	0.36	1.62	2.27	0.58	0.89	0.60
KEAP1/STK11 mt	NA	NA	NA	NA	NA	1.11	0.21	0.76	0.92	0.29	5.73
RAS/RAF/RTK mt	NA	NA	NA	NA	NA	NA	0.72	0.67	1.81	1.03	1.75
EGFR ge	NA	NA	NA	NA	NA	NA	NA	1.05	1.37	2.11	0.22
p16/CDKN2A ge	NA	NA	NA	NA	NA	NA	NA	NA	0.60	1.12	0.68
NKX2-1 ge	NA	NA	NA	NA	NA	NA	NA	NA	NA	0.76	0.40
TP63 ge	NA	NA	NA	NA	NA	NA	NA	NA	NA	NA	0.58
DUSP4 ge	NA	NA	NA	NA	NA	NA	NA	NA	NA	NA	NA

P-values	EGFR mt	EGFR mSig	EGFR cn	TP53 mt	KEAP1/STK11 mt	RAS/RAF/RTK mt	EGFR ge	p16/CDKN2A ge	NKX2-1 ge	TP63 ge	DUSP4 ge
Bronchioid	8.97E-08	4.29E-21	3.27E-04	1.06E-11	2.73E-06	5.92E-02	5.86E-02	8.88E-02	1.50E-12	2.85E-07	1.27E-05
Magnoid	1.32E-05	2.96E-12	6.90E-01	6.18E-01	5.33E-22	6.19E-01	1.81E-08	4.63E-02	5.50E-01	6.31E-14	2.28E-15
Squamoid	0.092	0.000	0.000	0.000	0.000	0.182	0.001	0.000	0.000	0.070	0.003
EGFR mt	NA	0.000	0.000	1.000	0.000	0.000	0.000	0.893	0.140	0.044	0.000
EGFR mSig	NA	NA	0.000	0.441	0.000	0.000	0.000	0.583	0.000	0.003	0.000
EGFR cn	NA	NA	NA	0.000	0.012	0.000	0.000	0.102	0.011	0.204	0.000
TP53 mt	NA	NA	NA	NA	0.046	0.000	0.011	0.000	0.004	0.585	0.006
KEAP1/STK11 mt	NA	NA	NA	NA	NA	0.619	0.000	0.195	0.765	0.000	0.000
RAS/RAF/RTK mt	NA	NA	NA	NA	NA	NA	0.085	0.037	0.001	0.928	0.003
EGFR ge	NA	NA	NA	NA	NA	NA	NA	0.856	0.102	0.000	0.000
p16/CDKN2A ge	NA	NA	NA	NA	NA	NA	NA	NA	0.006	0.586	0.046
NKX2-1 ge	NA	NA	NA	NA	NA	NA	NA	NA	NA	0.147	0.000
TP63 ge	NA	NA	NA	NA	NA	NA	NA	NA	NA	NA	0.004
DUSP4 ge	NA	NA	NA	NA	NA	NA	NA	NA	NA	NA	NA

Supplemental Table S5. Odds Ratio and p-value; within subtypes. mt, mutant, cn, copy number, ge, gene expression. Inf, infinite value due to zero count.

Bronchioid

Odds Ratio	EGFR mt	EGFR mSig	EGFR cn	TP53 mt	KEAP1/STK11 mt	RAS/RAF/RTK mt	EGFR ge	p16/CDKN2A ge	NKX2-1 ge	TP63 ge	DUSP4 ge
EGFR mt	NA	47.81	Inf	1.65	0.09	0.01	9.67	0.72	0.93	1.34	0.15
EGFR mSig	NA	NA	7.20	1.96	0.20	0.24	5.65	1.70	2.05	1.18	0.15
EGFR cn	NA	NA	NA	4.16	0.22	0.15	3.74	1.28	0.49	3.74	0.49
TP53 mt	NA	NA	NA	NA	0.73	0.51	2.04	1.34	0.59	1.34	0.89
KEAP1/STK11 mt	NA	NA	NA	NA	NA	1.61	0.08	1.26	2.54	0.21	4.74
RAS/RAF/RTK mt	NA	NA	NA	NA	NA	NA	0.31	1.55	1.55	0.97	2.26
EGFR ge	NA	NA	NA	NA	NA	NA	NA	1.41	1.41	1.54	0.19
p16/CDKN2A ge	NA	NA	NA	NA	NA	NA	NA	NA	2.04	1.07	0.68
NKX2-1 ge	NA	NA	NA	NA	NA	NA	NA	NA	NA	0.68	0.51
TP63 ge	NA	NA	NA	NA	NA	NA	NA	NA	NA	NA	0.74
DUSP4 ge	NA	NA	NA	NA	NA	NA	NA	NA	NA	NA	NA

P-values	EGFR mt	EGFR mSig	EGFR cn	TP53 mt	KEAP1/STK11 mt	RAS/RAF/RTK mt	EGFR ge	p16/CDKN2A ge	NKX2-1 ge	TP63 ge	DUSP4 ge
EGFR mt	NA	2.87E-15	1.15E-01	1.93E-01	2.01E-03	5.94E-18	4.72E-08	3.84E-01	8.62E-01	4.83E-01	1.38E-06
EGFR mSig	NA	NA	4.03E-02	5.26E-02	8.70E-04	7.97E-06	7.73E-08	9.56E-02	2.29E-02	6.50E-01	5.12E-09
EGFR cn	NA	NA	NA	2.75E-01	4.26E-02	7.95E-02	9.93E-02	7.47E-01	4.96E-01	9.93E-02	4.96E-01
TP53 mt	NA	NA	NA	NA	5.26E-01	5.18E-02	3.66E-02	4.23E-01	1.10E-01	4.23E-01	7.50E-01
KEAP1/STK11 mt	NA	NA	NA	NA	NA	3.09E-01	1.25E-06	6.85E-01	4.08E-02	4.89E-04	8.94E-04
RAS/RAF/RTK mt	NA	NA	NA	NA	NA	NA	2.43E-04	1.72E-01	1.72E-01	1.00E+00	9.68E-03
EGFR ge	NA	NA	NA	NA	NA	NA	NA	2.91E-01	2.91E-01	1.75E-01	2.09E-07
p16/CDKN2A ge	NA	NA	NA	NA	NA	NA	NA	NA	2.35E-02	8.80E-01	2.28E-01
NKX2-1 ge	NA	NA	NA	NA	NA	NA	NA	NA	NA	2.28E-01	3.45E-02
TP63 ge	NA	NA	NA	NA	NA	NA	NA	NA	NA	NA	3.66E-01
DUSP4 ge	NA	NA	NA	NA	NA	NA	NA	NA	NA	NA	NA

Magnoid

Odds Ratio	EGFR mt	EGFR mSig	EGFR cn	TP53 mt	KEAP1/STK11 mt	RAS/RAF/RTK mt	EGFR ge	p16/CDKN2A ge	NKX2-1 ge	TP63 ge	DUSP4 ge
EGFR mt	NA	8.07	Inf	3.85	0.00	0.00	4.08	4.08	1.50	4.08	0.00
EGFR mSig	NA	NA	4.08	Inf	0.00	0.26	4.08	4.08	1.50	Inf	0.00
EGFR cn	NA	NA	NA	2.70	0.72	0.53	3.04	0.97	0.50	1.36	0.62
TP53 mt	NA	NA	NA	NA	0.34	0.15	2.40	5.00	0.49	1.09	0.24
KEAP1/STK11 mt	NA	NA	NA	NA	NA	2.61	0.50	0.64	0.72	0.81	2.66
RAS/RAF/RTK mt	NA	NA	NA	NA	NA	NA	1.03	0.33	1.44	0.92	6.13
EGFR ge	NA	NA	NA	NA	NA	NA	NA	0.97	0.78	1.52	0.97
p16/CDKN2A ge	NA	NA	NA	NA	NA	NA	NA	NA	0.56	1.36	0.39
NKX2-1 ge	NA	NA	NA	NA	NA	NA	NA	NA	NA	0.56	0.56
TP63 ge	NA	NA	NA	NA	NA	NA	NA	NA	NA	NA	0.62
DUSP4 ge	NA	NA	NA	NA	NA	NA	NA	NA	NA	NA	NA

P-values	EGFR mt	EGFR mSig	EGFR cn	TP53 mt	KEAP1/STK11 mt	RAS/RAF/RTK mt	EGFR ge	p16/CDKN2A ge	NKX2-1 ge	TP63 ge	DUSP4 ge
EGFR mt	NA	0.17	0.06	0.37	0.01	0.06	0.37	0.37	1.00	0.37	0.03
EGFR mSig	NA	NA	0.37	0.06	0.01	0.37	0.37	0.37	1.00	0.06	0.03
EGFR cn	NA	NA	NA	0.00	0.39	0.07	0.00	1.00	0.05	0.40	0.18
TP53 mt	NA	NA	NA	NA	0.00	0.00	0.01	0.00	0.04	0.87	0.00
KEAP1/STK11 mt	NA	NA	NA	NA	NA	0.01	0.06	0.23	0.39	0.61	0.01
RAS/RAF/RTK mt	NA	NA	NA	NA	NA	NA	1.00	0.00	0.32	0.87	0.00
EGFR ge	NA	NA	NA	NA	NA	NA	NA	1.00	0.51	0.24	1.00
p16/CDKN2A ge	NA	NA	NA	NA	NA	NA	NA	NA	0.10	0.40	0.01
NKX2-1 ge	NA	NA	NA	NA	NA	NA	NA	NA	NA	0.10	0.10
TP63 ge	NA	NA	NA	NA	NA	NA	NA	NA	NA	NA	0.18
DUSP4 ge	NA	NA	NA	NA	NA	NA	NA	NA	NA	NA	NA

Squamoid

Odds Ratio	EGFR mt	EGFR mSig	EGFR cn	TP53 mt	KEAP1/STK11 mt	RAS/RAF/RTK mt	EGFR ge	p16/CDKN2A ge	NKX2-1 ge	TP63 ge	DUSP4 ge
EGFR mt	NA	20.53	2.52	0.90	0.34	0.00	17.82	1.32	2.37	1.32	0.57
EGFR mSig	NA	NA	1.85	0.73	0.25	0.33	7.29	1.11	2.81	1.11	0.27
EGFR cn	NA	NA	NA	1.21	0.70	1.00	4.42	1.30	1.17	1.78	0.95
TP53 mt	NA	NA	NA	NA	1.79	0.58	2.11	2.82	1.60	0.55	0.35
KEAP1/STK11 mt	NA	NA	NA	NA	NA	0.23	0.69	1.74	0.31	0.83	1.74
RAS/RAF/RTK mt	NA	NA	NA	NA	NA	NA	1.05	0.65	1.87	0.87	1.40
EGFR ge	NA	NA	NA	NA	NA	NA	NA	0.75	1.95	1.46	0.62
p16/CDKN2A ge	NA	NA	NA	NA	NA	NA	NA	NA	0.51	1.21	1.46
NKX2-1 ge	NA	NA	NA	NA	NA	NA	NA	NA	NA	0.68	0.34
TP63 ge	NA	NA	NA	NA	NA	NA	NA	NA	NA	NA	1.46
DUSP4 ge	NA	NA	NA	NA	NA	NA	NA	NA	NA	NA	NA

P-values	EGFR mt	EGFR mSig	EGFR cn	TP53 mt	KEAP1/STK11 mt	RAS/RAF/RTK mt	EGFR ge	p16/CDKN2A ge	NKX2-1 ge	TP63 ge	DUSP4 ge
EGFR mt	NA	5.05E-07	1.78E-01	1.00E+00	4.71E-01	2.98E-05	2.84E-04	7.94E-01	1.88E-01	7.94E-01	4.31E-01
EGFR mSig	NA	NA	3.30E-01	5.82E-01	2.04E-01	3.58E-02	7.20E-04	1.00E+00	6.00E-02	1.00E+00	1.79E-02
EGFR cn	NA	NA	NA	7.02E-01	5.03E-01	1.00E+00	2.10E-05	5.18E-01	7.47E-01	1.06E-01	1.00E+00
TP53 mt	NA	NA	NA	NA	4.49E-01	1.45E-01	6.68E-02	9.95E-03	2.73E-01	1.43E-01	9.95E-03
KEAP1/STK11 mt	NA	NA	NA	NA	NA	4.65E-03	5.23E-01	2.86E-01	1.77E-02	8.31E-01	2.86E-01
RAS/RAF/RTK mt	NA	NA	NA	NA	NA	NA	1.00E+00	2.15E-01	6.28E-02	7.57E-01	3.53E-01
EGFR ge	NA	NA	NA	NA	NA	NA	NA	4.41E-01	4.45E-02	2.80E-01	1.65E-01
p16/CDKN2A ge	NA	NA	NA	NA	NA	NA	NA	NA	4.45E-02	6.44E-01	2.80E-01
NKX2-1 ge	NA	NA	NA	NA	NA	NA	NA	NA	NA	2.80E-01	1.13E-03
TP63 ge	NA	NA	NA	NA	NA	NA	NA	NA	NA	NA	2.80E-01
DUSP4 ge	NA	NA	NA	NA	NA	NA	NA	NA	NA	NA	NA

Supplemental Table S6. Driver mutation index of TCGA LUAD samples. Genes included in this study were curated and others downloaded from cBioPortal. Subtype: 1, Bronchioid; 2, Magnoid; 3, Squamoid. mSig: 1, mSig(+); 2, mSig(-). Mutation: 1, altered; 2, No alteration.

sampleID	Subtype	mSig	RAS_type	EGFR	EGFR_CN	TP53	CDKN2A	CDKN2A_CN	KEAP1	STK11	KRAS	NRAS	HRAS	BRAF	ERBB2	ALK	MET	ROS1	PIK3CA	MAP2K1	RIT1	PPP3CA	SOS1	VAV1	AKT1	CDK4	ERBB4	EPHA3	KDR	FGFR4	NTRK1	NTRK2	NTRK3	GNAS	U2AF1	RB1	NF1	SMARCA4	RBM10	ATM	ARID1A	APC	LRP1B	MGA	SETD2	DOT1L	CMTR2	KLF5	EP300	CREBBP	B2M	TRAF3	ARHGAP35
TCGA-05-4244	1	2	K-Ox	2	2	2	2	2	2	2	1	2	2	2	2	2	2	2	2	2	2	2	2	2	2	2	2	2	2	1	2	2	2	2	2	2	2	2	2	1	2	2	2	2	2	2	2	2	2	2	2	2	2
TCGA-05-4249	1	2	K-Ox	2	2	2	2	2	2	2	1	2	2	2	2	2	2	2	1	2	1	2	2	2	2	2	2	2	2	2	1	2	2	2	2	2	2	2	2	2	2	2	1	2	2	2	2	2	2	2	2	2	2
TCGA-05-4250	3	2	K-TP53	2	4	2	2	2	2	2	1	2	2	2	2	2	2	2	2	2	2	2	2	2	2	2	2	2	2	2	2	2	1	2	2	2	2	2	2	2	2	2	2	2	2	2	2	2	2	1	2	2	2
TCGA-05-4382	3	2	RAS WT	2	4	1	1	1	2	2	2	2	2	2	2	2	2	2	2	2	2	2	2	2	2	2	1	2	1	2	2	2	2	2	2	2	2	2	2	1	1	1	1	2	2	2	2	2	2	2	2	2	2
TCGA-05-4384	1	2	RAS WT	2	3	1	2	2	1	2	2	2	2	2	2	2	2	2	2	2	1	2	2	2	2	2	2	2	1	2	2	2	2	2	2	2	2	2	2	2	2	2	2	2	2	1	2	2	2	2	2	2	2
TCGA-05-4389	2	2	K-Ox	2	3	2	2	1	1	2	2	2	2	1	2	2	2	2	2	2	1	2	2	2	2	2	2	2	2	2	2	2	2	2	2	2	2	2	2	2	2	1	1	2	2	2	2	2	2	2	2	2	2
TCGA-05-4390	2	2	K-Ox	2	4	2	2	4	2	2	1	2	2	2	2	2	2	2	2	2	2	1	2	2	2	2	2	2	2	2	2	1	2	2	2	2	2	2	2	1	2	2	2	2	2	2	2	2	2	2	2	2	2
TCGA-05-4395	3	2	K-TP53	2	4	1	2	2	1	1	1	2	2	2	2	2	2	2	2	2	2	2	2	2	2	2	2	2	2	2	2	2	1	2	2	2	2	2	1	2	2	2	1	2	2	2	2	2	2	2	2	2	2
TCGA-05-4396	2	2	RAS WT	2	3	1	1	2	2	2	2	2	2	2	2	2	2	2	2	2	2	2	1	2	2	2	1	2	2	2	2	2	2	2	2	2	2	2	2	2	1	1	1	1	2	1	2	2	1	2	2	2	1
TCGA-05-4397	3	2	RAS WT	2	3	1	2	3	2	2	2	2	2	2	2	2	2	2	1	2	2	2	2	2	2	2	2	2	2	2	2	2	2	2	2	1	2	2	2	2	2	1	1	2	2	2	2	2	2	2	2	2	2
TCGA-05-4398	3	2	RAS WT	2	4	1	2	3	2	2	2	2	2	2	2	2	2	2	2	2	2	2	2	2	2	2	1	2	2	2	1	2	2	2	2	2	2	2	2	1	2	2	1	2	2	2	2	2	2	2	2	2	2
TCGA-05-4402	1	1	RAS WT	1	5	2	2	1	2	2	2	2	2	2	2	2	2	2	2	2	2	2	2	2	2	2	2	2	2	2	2	2	2	2	2	2	2	2	2	2	2	1	2	2	2	2	2	2	2	2	2	2	2
TCGA-05-4403	1	2	K-Ox	2	3	2	2	1	2	2	1	2	2	2	2	2	2	2	2	2	2	2	2	2	2	2	2	2	2	2	2	2	2	2	2	2	2	2	1	2	2	2	2	2	2	2	2	2	2	2	2	2	2
TCGA-05-4405	1	1	K-EGFR	2	4	1	2	2	2	2	1	2	2	2	2	2	2	2	2	2	2	2	2	1	2	2	2	2	2	2	2	2	2	2	2	2	2	2	2	2	2	2	1	1	2	2	2	2	2	2	2	2	2
TCGA-05-4410	1	2	K-Ox	2	4	1	2	4	2	2	2	2	2	1	2	2	2	2	2	2	2	2	2	2	2	2	2	1	2	2	2	2	2	2	2	2	2	2	2	2	2	2	1	2	2	1	2	2	2	2	2	2	2
TCGA-05-4415	2	2	K-Ox	2	5	1	2	2	2	1	1	2	2	2	2	2	2	2	2	2	2	2	2	2	2	2	2	2	2	2	2	2	2	2	2	1	2	2	2	2	2	2	2	2	2	2	2	2	2	2	2	2	2
TCGA-05-4417	2	2	K-Ox	2	4	2	2	3	2	1	1	2	2	2	2	2	2	2	2	2	2	2	2	2	2	2	2	2	2	2	2	2	2	2	2	2	2	2	2	1	2	2	2	2	2	2	2	2	2	2	2	1	2
TCGA-05-4418	2	2	K-Ox	2	4	2	2	1	1	2	1	2	2	2	2	2	2	2	2	2	1	2	2	2	2	2	2	2	2	2	2	2	2	2	2	2	2	1	1	2	2	2	1	1	2	2	2	2	2	2	2	2	2
TCGA-05-4420	2	2	K-Ox	2	4	1	2	2	2	2	2	2	1	2	2	2	2	2	2	2	2	2	2	2	2	2	2	2	2	2	2	2	2	2	2	2	1	2	2	2	2	2	2	2	2	2	1	2	2	2	2	2	2
TCGA-05-4422	2	2	RAS WT	2	3	2	2	3	2	2	2	2	2	2	2	2	2	2	2	2	1	2	2	2	2	2	2	2	2	2	1	2	2	1	2	2	2	2	2	1	2	2	2	2	2	2	2	2	2	2	2	2	2
TCGA-05-4424	1	2	RAS WT	2	3	1	2	2	2	2	2	2	2	2	2	2	2	2	2	2	2	2	2	2	2	2	2	2	1	2	1	1	2	1	2	2	1	2	1	2	2	2	1	2	2	2	2	2	2	2	2	2	2
TCGA-05-4425	3	2	RAS WT	2	3	2	2	3	2	2	2	2	2	2	2	2	2	2	2	2	2	2	2	2	2	2	2	2	2	2	2	2	2	2	2	2	2	2	2	2	1	2	2	2	1	2	2	2	2	2	2	2	2
TCGA-05-4426	3	2	K-TP53	2	3	1	1	3	2	2	2	2	2	2	2	2	2	1	2	2	2	2	2	2	2	2	2	2	2	1	2	2	2	2	2	2	2	2	2	2	2	2	2	2	2	2	2	2	2	1	2	2	2
TCGA-05-4427	3	2	K-TP53	2	2	1	1	4	2	2	1	2	2	2	2	2	2	2	2	2	1	2	1	2	2	2	1	1	2	2	1	2	2	2	2	2	2	2	2	2	1	2	1	2	1	2	2	2	2	1	2	2	2
TCGA-05-4430	1	2	K-Ox	2	3	1	2	3	2	2	1	2	2	2	2	2	2	2	2	2	2	2	2	2	2	2	2	2	1	2	2	1	2	1	2	2	2	2	2	2	2	2	1	2	2	2	2	2	2	2	2	2	2
TCGA-05-4432	2	2	RAS WT	2	3	1	1	1	2	2	2	2	2	2	2	2	2	2	2	2	2	2	2	2	2	2	1	1	2	2	2	2	2	1	2	2	2	1	2	2	2	2	1	2	2	2	2	2	2	2	2	2	2
TCGA-05-4433	1	2	K-Ox	2	3	1	1	2	2	2	1	2	2	2	2	2	2	2	2	2	2	2	2	2	2	2	2	2	2	2	2	2	2	2	2	2	2	2	2	2	2	2	2	2	2	2	2	2	2	2	2	2	2
TCGA-05-4434	3	2	K-TP53	2	3	2	1	3	2	2	2	2	2	2	1	2	2	2	2	2	2	2	2	2	2	2	2	2	2	2	2	2	2	2	2	2	2	2	2	2	2	2	2	2	2	2	2	2	2	2	2	2	2
TCGA-05-5420	3	2	K-TP53	2	3	2	2	2	2	2	1	2	2	2	2	2	2	2	NA	NA	NA	NA	NA	NA	NA	NA	NA	NA	NA	NA	NA	NA	NA	NA	NA	NA	NA	NA	NA	NA	NA	NA	NA	NA	NA	NA	NA	NA	NA	NA	NA	NA	NA
TCGA-05-5423	1	1	RAS WT	1	4	1	2	2	2	2	2	2	2	2	2	2	2	2	2	2	2	2	2	2	2	2	2	2	2	2	2	2	2	2	2	2	2	2	2	2	2	2	2	2	2	2	2	2	2	2	2	2	2
TCGA-05-5425	2	2	RAS WT	2	4	1	2	2	2	1	2	2	2	2	2	2	2	2	2	1	2	2	2	2	2	2	2	2	2	2	2	2	1	2	2	2	2	2	2	2	2	2	1	2	2	2	2	2	2	2	2	2	2
TCGA-05-5428	3	2	K-TP53	2	4	1	2	3	2	2	2	2	2	1	2	2	2	2	2	2	2	2	2	2	2	2	2	2	2	2	2	2	2	2	2	2	2	2	2	2	2	2	2	1	2	2	2	2	2	2	2	2	2
TCGA-05-5429	2	2	RAS WT	2	3	2	2	1	2	2	2	2	2	2	2	2	2	2	2	2	2	2	2	2	2	2	2	2	2	2	2	2	2	2	2	2	2	2	2	2	2	2	2	2	2	2	2	2	2	2	2	2	2
TCGA-05-5715	1	1	K-EGFR	2	3	2	2	1	2	2	2	2	2	2	1	2	2	2	2	2	2	2	2	2	2	2	2	2	2	2	2	2	2	2	2	2	2	2	2	2	1	2	2	1	2	2	2	2	2	2	2	2	2
TCGA-35-3615	1	2	K-Ox	2	3	2	2	3	1	1	1	2	2	2	2	2	2	2	2	2	1	2	2	2	2	2	2	2	2	2	2	2	2	2	2	2	2	2	1	2	2	2	1	2	2	2	2	2	2	2	2	2	2
TCGA-35-4122	3	2	RAS WT	2	3	1	2	1	2	2	2	2	2	2	2	2	2	2	2	2	2	2	2	2	2	2	2	2	2	2	2	2	2	2	2	2	2	2	2	2	2	2	2	2	2	2	2	2	2	2	2	2	2
TCGA-35-4123	3	2	K-TP53	2	4	1	2	3	2	2	2	1	2	2	2	2	2	2	2	2	2	2	2	2	2	2	2	2	2	2	2	2	2	2	2	2	2	2	2	2	2	2	1	2	2	2	2	2	2	2	2	2	2
TCGA-35-5375	3	2	RAS WT	2	3	1	2	3	1	2	2	2	2	2	2	2	2	2	2	2	2	2	2	2	2	2	2	2	2	2	2	2	2	2	2	1	2	1	2	2	2	2	1	1	2	2	2	1	2	2	2	2	2
TCGA-38-4625	3	2	RAS WT	2	3	1	1	1	2	2	2	2	2	2	2	2	2	2	2	1	2	2	2	2	2	2	2	2	2	2	2	2	1	2	2	2	2	2	2	2	2	2	1	2	2	2	1	2	2	2	2	2	2
TCGA-38-4626	1	2	K-Ox	2	3	2	2	3	2	2	1	2	2	2	2	2	2	2	2	2	2	2	2	2	2	2	2	2	1	2	2	2	2	2	2	2	2	2	2	2	2	2	1	2	2	2	2	2	2	2	2	2	2
TCGA-38-4627	3	2	RAS WT	1	4	2	2	2	2	2	2	2	2	2	2	2	2	2	2	2	2	2	2	2	2	2	2	2	2	2	2	2	2	2	2	2	2	2	2	2	2	2	2	2	2	2	2	2	2	2	2	2	2
TCGA-38-4628	3	1	RAS WT	1	5	2	2	1	2	2	2	2	2	2	2	2	2	2	2	2	1	2	2	2	2	1	2	2	2	2	1	2	2	2	2	2	2	2	2	2	2	2	2	1	2	2	2	2	2	2	2	2	2
TCGA-38-4629	3	2	RAS WT	2	4	1	2	1	2	2	2	2	2	2	2	2	2	2	NA	NA	NA	NA	NA	NA	NA	NA	NA	NA	NA	NA	NA	NA	NA	NA	NA	NA	NA	NA	NA	NA	NA	NA	NA	NA	NA	NA	NA	NA	NA	NA	NA	NA	NA
TCGA-38-4630	3	2	RAS WT	2	2	1	2	3	2	2	2	2	2	2	2	2	2	2	NA	NA	NA	NA	NA	NA	NA	NA	NA	NA	NA	NA	NA	NA	NA	NA	NA	NA	NA	NA	NA	NA	NA	NA	NA	NA	NA	NA	NA	NA	NA	NA	NA	NA	NA
TCGA-38-4631	2	2	RAS WT	2	4	2	2	1	1	2	2	2	2	2	2	2	2	2	2	2	2	2	2	2	2	2	1	2	1	2	2	2	2	1	2	2	1	1	2	2	1	1	1	2	1	2	2	2	2	2	2	2	2
TCGA-38-4632	3	2	K-TP53	2	4	1	1	2	2	2	2	1	2	2	2	2	2	2	2	2	2	2	2	2	2	2	2	1	2	2	2	2	1	2	2	2	2	2	2	2	2	2	1	2	2	2	2	2	2	2	2	2	2
TCGA-38-6178	1	1	RAS WT	1	5	1	2	1	2	2	2	2	2	2	2	2	2	2	2	2	2	2	2	2	2	2	2	1	2	2	2	2	2	2	2	2	2	2	1	2	2	2	2	2	2	2	2	2	2	2	2	2	2
TCGA-38-7271	1	2	RAS WT	2	3	2	2	3	2	2	2	2	2	2	2	2	2	2	2	2	2	2	2	2	2	2	2	2	2	1	2	2	2	2	2	2	2	2	2	2	2	1	1	2	2	2	2	2	2	2	2	2	2
TCGA-38-A44F	1	2	K-Ox	2	3	2	2	2	2	2	1	2	2	2	2	2	2	2	2	2	2	2	2	2	2	2	2	2	2	2	2	2	2	2	2	2	2	2	2	2	2	2	2	2	2	2	2	2	2	2	2	2	2
TCGA-44-2655	1	2	K-Ox	2	3	2	2	2	1	2	1	2	2	2	2	2	2	2	2	2	2	2	2	2	2	2	2	2	2	2	2	2	2	2	2	2	2	2	2	2	2	2	2	1	2	2	2	2	2	2	2	2	2
TCGA-44-2656	3	2	K-TP53	2	4	1	2	3	2	2	2	2	2	2	1	2	2	2	2	2	2	2	2	2	2	2	2	2	2	2	2	2	2	2	2	2	1	1	2	2	2	2	2	2	2	2	2	2	2	2	2	2	2
TCGA-44-2657	1	1	RAS WT	2	4	2	2	2	2	2	2	2	2	2	2	2	2	2	2	2	2	2	2	2	2	1	2	1	2	2	2	2	2	2	2	2	1	2	2	2	2	2	2	2	2	2	2	2	2	2	2	2	2
TCGA-44-2659	1	1	RAS WT	2	4	1	2	2	2	2	2	2	2	2	2	2	2	2	2	2	2	2	2	2	2	2	2	2	1	2	2	2	2	2	2	2	1	2	2	2	2	2	1	2	2	2	2	2	2	2	2	2	1
TCGA-44-2661	1	1	RAS WT	1	4	2	2	2	2	2	2	2	2	2	2	2	2	2	NA	NA	NA	NA	NA	NA	NA	NA	NA	NA	NA	NA	NA	NA	NA	NA	NA	NA	NA	NA	NA	NA	NA	NA	NA	NA	NA	NA	NA	NA	NA	NA	NA	NA	NA
TCGA-44-2662	3	2	K-TP53	2	4	1	2	2	2	2	2	2	2	1	2	2	2	2	2	2	2	2	2	2	2	2	2	2	2	2	2	2	2	2	2	2	2	2	2	2	2	2	2	2	2	2	2	2	2	2	2	2	2
TCGA-44-2665	3	2	K-TP53	2	3	2	2	1	2	2	2	2	2	2	2	2	1	2	2	2	2	2	1	2	2	2	2	2	2	2	2	2	2	2	2	2	2	2	2	2	2	2	2	2	2	2	2	2	2	2	2	2	2
TCGA-44-2666	1	2	K-Ox	2	3	1	2	3	2	2	2	2	2	1	2	2	2	2	2	2	2	2	2	2	2	2	2	2	2	2	2	2	2	2	2	2	2	2	2	2	2	2	2	2	1	2	2	2	2	2	2	2	2
TCGA-44-2668	3	2	K-TP53	2	3	1	2	2	2	2	1	2	2	2	2	2	2	2	1	2	2	2	2	2	2	1	2	2	2	2	2	2	2	2	2	2	2	2	2	2	2	2	1	2	2	2	2	2	2	2	2	2	2
TCGA-44-3396	3	2	K-TP53	2	4	1	2	3	2	2	2	2	2	2	1	2	2	2	2	2	2	2	2	2	2	1	2	2	2	2	2	2	2	2	2	2	2	2	2	2	2	2	2	2	2	2	2	2	2	2	2	2	2
TCGA-44-3398	3	2	K-TP53	2	3	2	2	3	2	2	1	2	2	2	2	2	2	2	2	2	2	2	2	2	2	2	2	2	2	2	2	2	2	2	2	2	2	2	2	2	2	2	1	2	2	2	1	2	2	2	2	2	2
TCGA-44-3918	3	2	RAS WT	2	4	1	1	3	2	2	2	2	2	2	2	2	2	2	2	2	2	2	2	2	2	2	2	2	2	2	2	2	1	2	2	2	1	2	2	2	2	2	1	1	2	2	2	2	2	2	2	2	2
TCGA-44-3919	1	1	K-EGFR	2	4	2	2	3	2	2	2	2	2	2	1	2	2	2	2	2	2	2	2	2	2	2	2	2	2	2	2	2	2	2	2	2	2	2	2	2	2	2	2	2	2	2	2	2	2	2	2	2	2
TCGA-44-4112	3	2	RAS WT	2	4	2	2	3	2	2	2	2	2	2	2	2	2	2	2	2	2	2	2	2	2	2	1	2	1	2	2	2	1	2	2	2	2	2	1	2	2	2	1	2	2	2	1	2	2	2	2	2	1
TCGA-44-5643	2	2	RAS WT	2	2	1	1	2	2	2	2	2	2	2	2	2	2	2	1	2	2	2	2	2	2	2	2	2	2	2	2	2	2	2	2	2	2	2	2	2	2	2	2	2	2	2	2	2	2	2	2	2	2
TCGA-44-5644	2	2	RAS WT	2	4	1	2	1	2	2	2	2	2	2	2	2	2	2	2	2	2	2	2	2	2	1	1	1	2	1	2	2	2	2	2	2	1	2	2	2	2	2	1	1	2	2	2	2	2	2	2	2	1
TCGA-44-5645	1	1	RAS WT	1	4	1	1	3	2	2	2	2	2	2	2	2	2	2	1	2	2	2	2	2	2	2	2	1	2	2	2	2	2	2	2	2	2	2	2	2	2	2	2	2	2	2	1	2	2	2	2	2	2
TCGA-44-6145	3	2	K-TP53	2	2	1	2	3	2	2	1	2	2	2	2	2	2	2	2	2	2	2	2	2	2	2	2	2	2	2	2	2	2	2	2	2	2	2	2	2	2	2	1	2	2	2	2	2	2	2	2	2	2
TCGA-44-6146	1	2	K-Ox	2	3	2	2	1	2	2	1	2	2	2	2	2	2	2	2	2	2	2	2	2	2	2	2	2	2	2	2	2	2	2	2	2	2	2	2	2	2	2	2	2	2	2	2	2	2	2	2	2	2
TCGA-44-6147	1	1	RAS WT	1	4	1	2	4	2	2	2	2	2	2	2	2	2	2	NA	NA	NA	NA	NA	NA	NA	NA	NA	NA	NA	NA	NA	NA	NA	NA	NA	NA	NA	NA	NA	NA	NA	NA	NA	NA	NA	NA	NA	NA	NA	NA	NA	NA	NA
TCGA-44-6148	1	2	RAS WT	2	3	2	2	3	2	2	2	2	2	2	2	2	2	2	2	2	2	2	2	2	2	2	2	2	2	2	2	2	2	2	2	2	2	2	2	2	2	2	2	2	2	2	2	2	2	2	2	2	2
TCGA-44-6774	3	2	K-TP53	2	3	1	2	4	2	2	2	2	2	1	2	2	2	2	2	2	2	2	2	2	2	2	2	2	2	2	2	2	2	2	2	2	2	2	2	2	2	2	1	2	2	2	2	2	2	2	2	2	2
TCGA-44-6775	3	1	RAS WT	2	4	1	2	3	2	2	2	2	2	2	2	2	1	2	2	2	2	2	2	2	2	2	2	2	2	2	2	2	2	2	2	1	1	2	2	2	2	2	2	2	2	2	2	2	2	2	2	2	2
TCGA-44-6776	1	2	K-Ox	2	3	2	2	3	1	1	1	2	2	2	2	2	2	2	2	2	2	2	2	2	2	2	2	2	1	2	2	2	2	2	2	2	2	2	2	2	2	2	2	2	2	2	2	2	2	2	2	2	2
TCGA-44-6777	3	2	K-TP53	2	4	1	2	2	2	2	1	2	2	2	2	2	2	2	2	2	2	2	2	2	2	2	1	2	2	2	2	2	2	2	2	2	2	2	2	2	2	2	2	2	2	2	2	2	2	2	2	2	2
TCGA-44-6778	1	2	RAS WT	2	3	1	2	2	1	2	2	2	2	2	2	2	2	2	2	1	2	2	2	2	2	2	1	2	2	2	2	2	2	2	2	2	2	2	2	2	2	2	2	2	2	2	2	2	2	2	2	2	2
TCGA-44-6779	3	2	RAS WT	2	4	1	2	1	1	1	2	2	2	2	2	2	2	2	2	2	2	2	2	2	2	2	2	1	2	2	2	2	2	2	2	2	2	2	2	2	2	2	1	2	2	2	2	2	2	2	2	2	2
TCGA-44-7659	1	2	K-Ox	2	3	2	2	3	2	2	1	2	2	2	2	2	2	2	2	2	2	2	2	2	2	2	2	2	2	2	1	2	2	2	2	2	2	2	1	2	2	2	2	2	2	2	2	2	2	2	2	2	2
TCGA-44-7660	3	2	RAS WT	2	4	1	1	4	1	2	2	2	2	2	2	2	2	2	1	2	2	2	2	2	2	1	2	2	2	2	2	2	2	2	2	1	2	2	2	2	2	2	2	2	2	2	2	2	2	2	2	2	2
TCGA-44-7661	3	2	K-TP53	2	4	1	2	3	2	2	1	2	2	2	2	2	2	2	2	2	2	2	2	2	2	2	2	2	2	2	2	2	2	2	2	2	2	2	1	2	2	1	1	2	2	2	2	2	2	2	2	2	2
TCGA-44-7662	3	2	RAS WT	2	4	1	1	2	2	2	2	2	2	2	2	2	2	2	2	2	2	2	2	2	2	2	1	2	2	2	2	1	2	2	2	2	2	2	2	1	2	2	1	2	2	2	2	2	2	2	2	2	2
TCGA-44-7667	2	2	RAS WT	2	4	1	2	2	1	2	2	2	2	2	2	2	2	2	2	2	2	2	1	2	2	2	1	2	2	2	2	2	2	2	2	2	1	2	2	2	1	2	2	2	2	2	2	2	2	2	2	2	2
TCGA-44-7669	2	2	RAS WT	2	4	1	2	2	1	2	2	2	2	2	2	2	2	2	2	2	2	2	2	2	2	2	2	2	2	2	2	2	2	2	2	1	2	2	2	2	2	2	1	2	2	2	2	2	2	2	2	2	2
TCGA-44-7670	2	2	RAS WT	2	2	1	2	2	2	2	2	2	2	2	2	2	2	2	1	2	2	2	2	1	2	2	1	2	2	2	1	2	1	2	2	2	2	2	2	2	1	2	2	1	2	2	2	1	2	1	2	2	2
TCGA-44-7671	2	2	K-Ox	2	3	2	2	2	1	1	1	2	2	2	2	2	2	2	2	2	2	2	2	2	2	2	2	2	2	2	2	2	2	2	2	2	2	2	2	2	2	2	2	1	2	2	2	2	2	2	2	2	2
TCGA-44-7672	3	2	K-TP53	2	3	2	1	3	2	2	1	2	2	2	2	2	2	2	2	2	2	2	2	2	1	1	2	2	2	2	2	2	2	2	2	2	2	2	2	2	2	2	2	2	2	2	2	2	2	2	2	2	2
TCGA-44-8117	3	2	K-TP53	2	4	1	2	2	2	2	1	2	2	2	2	2	2	2	2	2	2	2	2	2	2	2	2	2	2	2	2	2	2	2	2	2	2	1	2	2	2	2	1	1	2	2	2	2	2	2	2	2	2
TCGA-44-8119	2	2	RAS WT	2	2	2	2	4	2	2	2	2	2	2	2	2	2	2	2	2	2	2	2	2	2	2	1	2	2	1	2	1	2	2	2	2	1	1	2	2	2	2	1	2	2	2	2	2	2	1	2	2	2
TCGA-44-8120	1	1	RAS WT	2	4	1	2	2	2	1	2	2	2	2	2	2	2	2	2	2	2	2	2	2	2	2	2	2	2	2	2	2	1	2	2	2	2	2	2	2	2	2	2	2	2	2	2	2	2	2	2	2	2
TCGA-44-A479	2	2	RAS WT	2	4	1	2	3	2	1	2	2	2	2	2	2	2	2	2	2	2	2	2	2	2	2	2	2	2	2	2	2	1	1	2	2	2	1	2	2	2	2	2	2	2	2	2	2	2	2	2	2	2
TCGA-44-A47A	1	2	K-Ox	2	3	2	2	3	2	2	1	2	2	2	2	2	2	2	2	2	2	2	2	2	2	2	2	2	2	2	2	2	2	2	2	2	2	2	1	2	2	2	1	2	2	2	2	2	2	2	2	2	2
TCGA-44-A47B	2	2	K-Ox	2	2	2	2	3	2	2	2	2	2	1	2	2	2	2	2	2	2	2	2	2	2	2	2	2	2	2	2	2	2	2	2	2	2	2	2	2	2	2	2	2	2	2	2	2	2	2	2	2	2
TCGA-44-A47G	3	2	K-TP53	2	4	1	1	3	2	2	2	2	2	2	2	2	1	2	2	2	2	2	2	2	2	2	2	2	2	2	2	2	2	2	2	2	2	2	2	2	2	2	2	2	2	2	2	2	2	2	2	2	2
TCGA-44-A4SS	3	2	RAS WT	2	4	1	2	2	1	2	2	2	2	2	2	2	2	2	2	2	2	2	1	2	2	2	2	1	1	2	2	2	1	2	2	2	2	2	2	2	2	1	1	1	2	2	2	2	2	2	1	1	2
TCGA-44-A4SU	1	1	RAS WT	1	5	1	2	1	2	2	2	2	2	2	2	2	2	2	2	2	2	2	2	2	2	2	2	2	1	2	2	2	2	2	2	2	2	2	2	2	2	2	2	1	2	2	2	2	2	2	2	2	2
TCGA-49-4486	1	2	RAS WT	2	3	2	2	2	1	1	2	2	2	2	2	2	2	2	2	2	2	2	2	2	2	2	2	2	2	2	2	2	2	2	2	2	2	2	2	2	2	2	2	2	2	1	2	2	2	2	2	2	2
TCGA-49-4487	3	2	RAS WT	2	3	1	2	2	2	1	2	2	2	2	2	2	2	2	2	2	1	2	2	2	2	2	2	1	2	2	1	2	2	2	2	2	2	2	2	2	2	2	1	2	1	2	2	2	2	2	2	2	2
TCGA-49-4488	3	2	RAS WT	2	4	1	1	4	1	2	2	2	2	2	2	2	2	2	2	2	2	2	2	2	2	2	2	2	2	2	2	2	2	2	1	2	1	2	2	2	2	2	1	2	2	2	2	2	2	2	2	2	2
TCGA-49-4490	1	1	RAS WT	1	4	1	2	1	2	2	2	2	2	2	2	2	2	2	2	2	2	2	2	2	2	2	2	2	2	2	2	2	2	2	2	2	2	2	2	2	2	2	2	2	2	2	2	2	2	2	2	2	2
TCGA-49-4494	2	2	RAS WT	1	4	2	2	1	2	2	2	2	2	2	2	2	2	2	2	2	2	2	2	2	2	2	2	2	2	2	1	2	2	2	2	2	2	2	1	2	2	2	1	1	2	2	2	2	2	2	2	2	2
TCGA-49-4501	1	1	RAS WT	1	5	2	2	2	2	2	2	2	2	2	2	2	2	2	2	2	2	2	2	2	2	2	2	2	2	2	2	2	2	2	2	2	2	2	2	2	1	2	2	2	2	2	2	2	2	2	2	2	2
TCGA-49-4505	1	1	K-EGFR	2	4	1	1	2	2	2	1	2	2	2	2	2	2	2	2	2	2	2	2	2	2	2	2	2	2	2	2	2	2	2	1	2	2	2	2	2	2	2	2	2	2	2	2	2	2	2	2	2	2
TCGA-49-4506	2	2	K-Ox	2	4	2	2	2	1	2	1	2	2	2	2	2	2	2	2	2	1	2	2	2	2	2	2	2	2	2	1	2	2	2	2	2	2	2	2	2	2	2	2	2	2	2	2	2	2	2	2	2	2
TCGA-49-4507	3	2	RAS WT	2	3	2	1	1	1	1	2	2	2	2	2	2	2	2	2	2	1	2	2	2	2	1	2	2	2	2	2	2	2	2	2	2	2	2	2	1	2	1	2	2	2	2	2	2	2	2	2	2	1
TCGA-49-4510	1	1	K-EGFR	2	4	2	2	2	2	2	1	2	2	2	2	2	2	2	2	2	2	2	2	2	2	2	2	2	2	2	2	2	2	2	2	2	2	2	2	1	2	2	2	2	2	2	2	2	2	2	2	2	2
TCGA-49-4512	1	1	RAS WT	1	4	2	2	3	2	2	2	2	2	2	2	2	2	2	2	2	2	2	2	2	2	2	2	2	2	2	2	2	2	2	2	2	2	2	2	2	2	2	2	2	2	2	2	2	2	2	2	2	2
TCGA-49-4514	2	2	RAS WT	2	3	1	2	2	1	2	2	2	2	2	2	2	2	2	2	2	1	2	2	2	2	2	2	2	2	2	2	2	1	2	2	2	2	2	2	2	2	2	2	2	2	2	2	2	2	2	2	2	2
TCGA-49-6742	2	2	RAS WT	2	4	2	2	1	1	2	2	2	2	2	2	2	2	2	2	2	2	2	2	2	2	2	2	2	2	2	2	2	2	2	2	2	2	1	2	2	2	2	1	2	2	2	2	2	2	2	2	2	1
TCGA-49-6743	2	2	RAS WT	2	3	2	2	2	1	2	2	2	2	2	2	2	2	2	1	2	2	2	2	2	2	2	2	2	2	2	1	2	2	2	2	2	2	2	2	2	2	2	1	1	2	2	2	2	2	2	2	2	2
TCGA-49-6744	1	2	K-Ox	2	3	2	2	3	2	2	1	2	2	2	2	2	2	2	2	2	2	2	2	2	2	2	2	2	2	2	2	2	2	2	1	2	2	2	2	2	2	2	2	2	1	2	2	2	2	2	2	2	2
TCGA-49-6745	3	2	K-TP53	2	4	2	2	1	2	2	2	2	2	2	2	2	1	2	2	2	2	2	1	2	2	1	2	2	2	2	2	2	2	2	2	2	2	2	2	2	2	2	1	1	2	2	2	2	2	2	1	2	2
TCGA-49-6761	3	2	K-TP53	2	4	2	2	3	2	2	1	2	2	2	2	2	2	2	2	2	2	2	2	2	2	2	2	2	2	2	2	2	2	2	2	2	2	2	2	2	2	1	2	2	2	2	1	2	2	2	2	2	2
TCGA-49-6767	3	2	RAS WT	2	4	1	2	1	2	2	2	2	2	2	2	2	2	2	2	2	2	2	2	2	2	2	1	2	2	2	2	2	2	2	2	2	1	1	2	2	2	2	2	2	2	1	2	2	2	2	2	2	2
TCGA-50-5044	3	2	K-TP53	2	3	2	2	3	2	2	2	2	2	1	2	2	2	2	1	2	2	2	2	2	2	2	2	2	2	2	2	2	2	2	2	2	2	2	2	1	2	2	2	2	1	2	2	2	2	2	2	2	2
TCGA-50-5045	3	2	RAS WT	2	4	1	2	3	2	2	2	2	2	2	2	2	2	2	2	2	2	2	2	2	2	2	2	1	2	2	2	2	2	2	2	2	1	2	2	2	2	2	2	2	2	2	2	2	2	2	2	2	2
TCGA-50-5049	3	2	RAS WT	2	4	1	2	3	2	2	2	2	2	2	2	2	2	2	2	2	2	2	2	2	2	2	1	2	1	2	2	2	2	1	2	2	2	2	2	2	2	1	1	2	2	2	2	2	2	2	2	2	2
TCGA-50-5051	2	2	K-Ox	2	3	2	1	4	1	2	1	2	2	2	2	2	2	2	2	2	2	2	2	2	2	2	2	2	2	2	2	2	2	2	2	2	2	2	2	2	2	2	1	2	2	2	2	2	2	2	2	2	2
TCGA-50-5055	1	2	K-Ox	2	4	2	2	2	2	2	2	2	2	2	2	2	1	2	2	2	2	2	2	2	2	1	2	1	2	2	2	2	2	2	2	2	2	2	2	2	2	2	2	2	2	2	2	2	2	2	2	2	2
TCGA-50-5066	3	2	RAS WT	2	4	1	2	3	1	2	2	2	2	2	2	2	2	2	2	2	2	2	2	2	2	2	2	2	1	2	2	2	2	2	2	1	2	2	2	2	2	2	2	2	2	2	2	2	2	2	2	2	2
TCGA-50-5068	3	2	RAS WT	2	3	1	2	1	1	2	2	2	2	2	2	2	2	2	2	2	2	2	1	2	2	2	2	2	2	2	2	2	2	2	2	2	1	2	2	2	2	2	2	2	2	2	2	2	2	2	2	2	2
TCGA-50-5072	2	2	RAS WT	2	4	2	2	1	2	1	2	2	2	2	2	2	2	2	2	2	2	2	2	2	2	2	2	2	2	2	2	2	2	2	2	2	1	1	2	2	2	2	2	2	2	1	2	2	2	2	2	2	2
TCGA-50-5930	2	2	K-Ox	2	4	1	2	1	2	2	2	2	2	1	2	2	2	2	2	1	2	2	2	2	2	2	2	1	2	2	2	2	1	2	2	2	2	1	2	2	2	1	1	2	2	2	1	2	2	2	2	2	2
TCGA-50-5931	2	2	RAS WT	2	2	1	2	2	2	2	2	2	2	2	2	2	2	2	2	2	2	2	2	2	2	2	2	2	2	2	2	2	2	2	2	1	2	2	2	2	2	2	2	1	2	2	2	2	2	2	2	2	2
TCGA-50-5932	2	2	K-Ox	2	3	2	2	1	2	1	1	2	2	2	2	2	2	2	2	2	2	2	2	2	2	2	2	1	2	2	2	2	2	2	2	2	2	2	2	2	2	2	2	2	2	2	2	2	2	2	2	2	2
TCGA-50-5933	3	2	K-TP53	2	5	1	2	2	2	2	1	2	2	2	2	2	2	2	2	2	2	2	2	2	2	2	2	1	2	2	2	2	2	2	2	2	2	2	2	2	2	2	1	2	2	2	2	2	2	2	2	2	2
TCGA-50-5935	1	1	RAS WT	1	3	2	2	2	2	2	2	2	2	2	2	2	2	2	2	2	1	2	2	2	2	2	2	2	2	2	1	2	2	2	2	2	2	2	2	2	2	2	2	2	2	2	2	2	2	2	2	2	2
TCGA-50-5936	2	2	K-Ox	2	4	2	2	2	1	2	1	2	2	2	2	2	2	2	2	2	2	2	2	2	2	2	2	2	2	2	2	2	2	2	2	2	2	1	2	2	2	2	1	2	2	2	2	2	2	2	2	2	2
TCGA-50-5939	3	2	RAS WT	2	5	1	2	2	1	1	2	2	2	2	2	2	2	2	2	2	2	2	2	2	2	1	2	1	2	2	2	2	2	2	2	2	2	2	2	2	2	2	2	2	2	2	2	2	2	2	2	2	2
TCGA-50-5941	3	2	K-TP53	2	4	1	1	3	2	2	1	2	2	2	2	2	2	2	2	2	2	2	2	2	2	2	2	2	2	2	2	2	2	2	1	2	2	2	2	2	2	2	1	2	1	2	2	2	2	2	2	2	2
TCGA-50-5942	1	1	K-EGFR	2	3	2	2	3	2	2	2	2	2	1	2	2	2	2	2	2	2	2	2	2	2	2	2	2	2	2	2	2	2	2	2	2	2	2	2	2	2	2	2	2	1	2	2	2	2	2	2	2	2
TCGA-50-5944	1	1	RAS WT	1	4	1	2	1	2	2	2	2	2	2	2	2	2	2	2	2	2	2	2	2	2	2	2	2	2	1	2	2	2	1	2	2	2	2	2	2	2	2	2	2	2	2	2	2	2	1	2	2	2
TCGA-50-5946	2	2	K-Ox	2	4	1	2	1	2	2	2	2	2	1	2	2	2	2	2	2	2	2	2	2	2	2	2	2	2	2	2	2	2	2	2	2	1	2	2	2	2	2	1	2	2	2	2	2	2	2	2	2	2
TCGA-50-6590	3	2	RAS WT	2	4	1	1	3	2	2	2	2	2	2	2	2	2	2	1	2	2	2	2	2	2	2	2	2	1	2	2	2	2	2	2	2	2	2	2	2	2	2	1	2	2	2	2	2	2	2	2	2	2
TCGA-50-6591	2	2	RAS WT	1	5	1	2	3	2	2	2	2	2	2	2	2	2	2	2	2	2	2	2	2	2	2	2	2	2	2	2	2	2	1	2	1	2	1	2	2	2	2	2	2	2	1	2	2	2	2	2	2	2
TCGA-50-6592	3	2	RAS WT	2	3	1	2	2	2	2	2	2	2	2	2	2	2	2	1	2	2	2	2	2	2	2	2	2	2	2	2	2	2	2	2	2	2	1	2	2	2	2	2	2	2	2	2	2	2	2	2	2	2
TCGA-50-6593	1	1	RAS WT	2	4	1	2	2	2	2	2	2	2	2	2	2	2	2	2	2	2	2	2	2	2	2	2	2	2	2	2	2	2	2	2	2	1	2	2	2	2	2	1	2	2	2	2	2	2	2	2	2	2
TCGA-50-6594	3	2	K-TP53	2	4	1	2	5	2	2	2	2	2	1	2	2	2	2	2	2	2	2	2	2	2	2	2	2	1	2	2	2	2	2	2	2	2	2	2	1	2	2	2	2	2	2	2	2	2	2	2	2	2
TCGA-50-6595	3	2	RAS WT	1	4	1	1	3	2	2	2	2	2	2	2	2	2	2	2	2	2	2	2	2	2	2	2	2	2	2	2	2	2	2	2	2	2	2	2	2	2	2	1	2	2	2	2	2	2	2	2	2	2
TCGA-50-6597	1	1	K-EGFR	2	2	2	2	2	2	2	2	2	2	2	2	2	1	2	2	2	2	2	2	2	2	2	2	2	2	2	2	2	2	2	2	2	2	2	2	2	2	2	2	2	2	2	2	2	2	2	2	2	2
TCGA-50-6673	1	2	RAS WT	1	5	1	2	2	2	2	2	2	2	2	2	2	2	2	2	2	2	2	2	2	2	2	2	2	2	2	2	2	2	2	2	2	2	2	2	2	2	2	2	2	2	2	2	2	2	2	2	2	2
TCGA-50-7109	2	2	K-Ox	2	3	2	2	2	2	1	1	2	2	2	2	2	2	2	2	2	2	2	2	2	2	2	2	2	2	2	2	2	2	2	2	2	2	2	2	2	2	2	1	2	2	2	2	2	2	2	2	2	2
TCGA-50-8457	1	1	K-EGFR	2	3	2	2	3	2	2	1	2	2	2	2	2	2	2	2	2	2	2	2	2	2	2	2	2	2	2	2	2	2	2	2	2	1	2	2	2	2	1	2	2	2	2	1	2	2	2	2	2	2
TCGA-50-8459	3	2	K-TP53	2	3	2	2	2	2	2	1	2	2	2	2	2	2	2	2	2	2	2	2	2	2	2	1	2	2	2	2	2	2	2	2	2	2	2	2	2	2	2	2	2	2	2	2	2	2	2	2	2	2
TCGA-50-8460	1	1	K-EGFR	2	3	1	2	2	2	2	2	2	2	2	2	1	2	2	2	2	2	2	2	2	2	2	2	2	2	2	2	2	2	2	1	2	2	2	2	2	2	2	2	2	2	2	2	2	2	2	2	2	2
TCGA-53-7624	3	2	RAS WT	2	4	1	2	1	1	2	2	2	2	2	2	2	2	2	2	2	2	2	2	2	2	2	1	2	2	2	2	2	2	2	2	2	2	1	2	1	2	2	1	2	1	2	2	2	2	2	2	2	2
TCGA-53-7626	1	2	K-Ox	2	3	1	1	2	2	2	1	2	2	2	2	2	2	2	2	2	2	2	2	2	1	2	1	2	2	1	2	2	1	2	2	2	2	2	2	2	2	2	1	2	2	2	2	2	1	2	2	1	1
TCGA-53-7813	2	2	K-Ox	2	2	2	2	2	2	1	1	2	2	2	2	2	2	2	2	2	2	2	2	2	2	2	2	2	2	2	2	2	1	2	2	2	2	2	2	1	2	2	2	2	2	2	2	2	2	2	2	2	2
TCGA-53-A4EZ	2	2	K-Ox	2	3	1	1	3	1	2	2	2	2	1	2	2	2	2	2	2	2	2	2	2	2	2	2	2	2	2	2	1	1	2	2	2	2	2	2	2	2	2	1	2	2	2	2	2	2	2	2	2	2
TCGA-55-1592	1	1	RAS WT	2	3	1	2	1	2	2	2	2	2	2	2	2	2	2	2	2	2	2	2	2	2	2	2	2	1	2	2	2	1	2	2	2	1	2	2	2	2	2	1	2	2	2	2	2	2	2	2	2	2
TCGA-55-1594	2	2	RAS WT	2	5	1	1	2	1	2	2	2	2	2	2	2	2	2	2	2	2	2	2	2	2	2	2	2	2	2	2	2	2	1	2	2	2	2	2	2	2	2	2	2	2	2	2	2	2	2	2	2	2
TCGA-55-1595	2	2	K-Ox	2	3	2	2	3	2	2	1	2	2	2	2	2	2	2	2	2	2	2	2	2	2	2	2	2	2	2	2	2	1	1	1	2	2	2	2	1	2	2	2	2	2	2	2	2	2	2	2	2	2
TCGA-55-1596	2	2	RAS WT	2	4	1	2	2	2	1	2	2	2	2	2	2	2	2	2	2	1	2	2	2	2	2	2	2	2	2	2	2	2	2	2	2	2	1	2	1	2	2	1	2	2	2	2	2	2	2	2	2	2
TCGA-55-5899	2	2	RAS WT	2	4	1	2	1	1	2	2	2	2	2	2	2	2	2	2	2	2	2	2	2	2	2	2	2	2	2	2	2	2	2	2	2	2	2	2	2	1	1	2	2	2	2	2	2	2	2	2	2	2

TCGA-55-6543	1	2	RAS WT	2	4	2	2	2	2	2	2	2	2	2	2	2	2	2	2	2	2	2	2	2	2	2	2	2	2	2	2	2	2	2	2	2	2	1	2	2	2	2	2	2	2	2	2	2	2	2	2	2	2
TCGA-55-6642	2	2	K-Ox	2	3	2	2	2	2	2	1	2	2	2	2	2	2	2	1	2	2	2	2	2	2	2	2	2	2	2	2	2	2	2	1	2	2	2	2	1	2	2	2	2	2	2	2	2	2	2	2	2	2
TCGA-55-6712	3	2	RAS WT	2	4	1	2	2	2	2	2	2	2	2	2	2	2	2	2	2	2	2	2	2	2	2	2	2	2	2	2	2	2	2	2	2	2	2	2	2	2	2	2	1	2	2	2	2	2	2	2	2	2
TCGA-55-6968	3	2	K-TP53	2	2	1	2	3	2	2	2	2	2	1	2	2	2	2	2	2	2	2	2	2	1	2	2	2	2	2	2	2	2	2	2	1	2	2	2	2	2	2	1	1	2	2	2	2	2	2	2	2	2
TCGA-55-6969	3	2	RAS WT	2	3	1	2	3	2	2	2	2	2	2	2	2	2	2	2	2	2	2	2	2	2	2	1	2	2	2	2	2	2	2	2	2	2	1	2	1	2	2	1	2	2	2	2	2	2	2	2	2	2
TCGA-55-6970	2	2	K-Ox	2	3	2	2	3	1	1	1	2	2	2	2	2	2	2	2	2	2	2	2	2	2	2	2	2	2	2	2	2	2	2	2	2	2	2	2	2	2	2	1	2	2	2	2	2	2	2	2	2	2
TCGA-55-6971	1	2	RAS WT	2	4	2	2	2	2	2	2	2	2	2	2	2	2	2	2	2	2	2	2	2	2	1	2	2	2	2	2	2	2	2	2	2	1	2	2	1	2	2	2	2	2	2	2	2	2	2	2	2	2
TCGA-55-6972	2	2	RAS WT	2	4	2	2	4	1	2	2	2	2	2	2	2	2	2	2	2	1	2	2	2	2	2	2	2	2	2	1	2	2	1	2	2	2	2	2	2	2	2	2	1	2	2	2	2	2	2	1	2	2
TCGA-55-6975	3	2	K-TP53	2	4	1	2	2	2	2	1	2	2	2	2	2	2	2	2	2	2	2	2	2	2	2	2	2	2	2	2	2	2	2	2	2	1	1	2	2	2	2	2	2	2	2	2	2	2	2	2	2	2
TCGA-55-6978	3	2	K-TP53	2	4	2	2	3	2	2	2	2	2	2	2	2	1	2	2	2	2	2	1	2	2	2	2	2	2	2	2	2	2	2	2	2	2	2	2	2	2	2	2	2	2	2	2	2	2	2	2	2	2
TCGA-55-6979	3	2	RAS WT	1	4	1	2	3	2	2	2	2	2	2	2	2	2	2	2	2	2	1	2	2	2	2	2	2	2	2	2	2	2	2	2	1	2	2	2	2	2	2	2	1	2	2	2	2	2	2	1	2	2
TCGA-55-6980	1	1	RAS WT	1	4	2	2	2	2	2	2	2	2	2	2	2	2	2	2	2	2	2	2	2	2	1	1	2	2	2	2	2	2	2	2	2	2	2	2	2	2	2	2	2	2	2	2	2	2	2	2	2	2
TCGA-55-6981	3	1	RAS WT	1	5	2	1	2	2	2	2	2	2	2	2	2	2	2	2	2	2	2	2	2	2	1	2	2	2	2	2	2	2	2	2	2	2	2	2	2	2	2	2	2	2	2	2	2	2	2	2	2	2
TCGA-55-6982	3	1	RAS WT	2	4	2	2	2	2	2	2	2	2	2	2	2	1	2	2	2	2	2	2	2	2	1	2	2	2	1	2	2	2	2	2	2	2	2	2	2	2	2	2	2	2	2	2	2	2	2	2	2	2
TCGA-55-6983	1	2	K-Ox	2	3	2	2	3	2	1	1	2	2	2	2	2	2	2	2	2	2	2	2	2	2	2	2	2	2	2	2	2	2	2	2	2	2	2	1	2	2	2	2	2	2	2	2	2	2	2	2	2	2
TCGA-55-6984	2	2	K-Ox	2	3	2	1	3	2	2	1	2	2	2	2	2	2	2	2	2	2	2	2	2	2	2	2	2	2	2	2	2	2	2	2	2	2	2	2	2	2	2	2	2	2	2	2	2	2	2	2	2	2
TCGA-55-6985	3	2	K-TP53	2	4	1	2	1	2	2	1	2	2	2	2	2	2	2	2	2	2	2	2	2	2	2	2	2	1	2	2	2	2	2	2	2	2	2	2	2	2	2	1	2	2	2	2	2	2	2	2	2	2
TCGA-55-6986	1	2	K-Ox	2	3	2	2	2	2	2	2	2	2	2	2	2	2	1	2	2	2	2	2	2	2	2	2	2	2	2	2	2	2	2	2	2	2	2	2	2	2	2	2	1	1	2	2	2	2	2	2	2	2
TCGA-55-6987	3	2	RAS WT	2	4	2	2	1	1	2	2	2	2	2	2	2	2	2	2	2	2	2	2	2	2	2	2	2	2	2	2	2	2	2	2	2	2	2	2	2	2	2	2	2	2	2	2	2	2	2	2	2	2
TCGA-55-7227	1	2	RAS WT	2	3	1	2	3	2	2	2	2	2	2	2	2	2	2	NA	NA	NA	NA	NA	NA	NA	NA	NA	NA	NA	NA	NA	NA	NA	NA	NA	NA	NA	NA	NA	NA	NA	NA	NA	NA	NA	NA	NA	NA	NA	NA	NA	NA	NA
TCGA-55-7281	1	1	K-EGFR	2	3	2	2	1	2	2	1	2	2	2	2	2	2	2	2	2	2	2	2	2	2	2	2	2	1	2	2	2	2	2	2	2	2	2	1	2	2	2	1	2	2	2	2	1	2	2	2	2	2
TCGA-55-7283	1	2	K-Ox	2	4	1	1	2	2	2	1	2	2	2	2	2	2	2	2	2	1	2	2	2	1	2	2	2	2	2	1	2	2	2	2	2	2	2	2	2	2	2	2	2	2	2	2	2	2	2	2	2	2
TCGA-55-7284	1	2	K-Ox	2	4	2	2	1	2	2	1	2	2	2	2	2	2	2	1	2	2	2	2	2	2	2	2	2	2	2	2	2	2	1	2	2	2	2	2	2	2	2	2	2	2	2	2	2	2	2	2	2	2
TCGA-55-7570	2	2	RAS WT	2	3	1	1	2	2	2	2	2	2	2	2	2	2	2	2	2	2	2	2	2	2	2	2	2	2	2	2	2	2	2	2	2	2	2	2	2	2	2	1	2	2	2	2	2	2	2	2	2	2
TCGA-55-7573	1	1	RAS WT	1	3	1	1	2	2	2	2	2	2	2	2	2	2	2	2	2	2	2	2	2	2	2	2	2	2	1	2	2	2	2	2	2	2	2	2	2	2	2	2	2	2	2	2	2	2	2	2	2	2
TCGA-55-7574	1	2	K-Ox	2	3	1	2	3	2	1	1	2	2	2	2	2	2	2	1	2	2	2	2	2	2	2	2	1	2	2	2	2	2	2	2	2	2	2	2	2	2	2	1	1	2	2	2	2	2	2	2	2	2
TCGA-55-7576	3	2	K-TP53	2	4	1	2	1	2	2	1	2	2	2	2	2	2	2	2	2	1	2	2	2	2	2	1	2	2	2	1	2	1	2	2	2	2	2	2	2	2	2	1	2	2	2	2	2	2	2	2	2	2
TCGA-55-7724	3	2	RAS WT	2	4	1	2	2	2	2	2	2	2	2	2	2	2	2	1	2	2	2	2	2	1	2	2	2	2	2	2	1	2	2	2	2	2	2	2	2	2	2	2	2	2	2	2	2	2	2	2	2	2
TCGA-55-7725	1	2	K-Ox	2	3	2	2	3	2	2	1	2	2	2	2	2	2	2	2	2	2	2	2	2	2	2	2	2	2	2	2	2	2	2	2	2	2	2	2	2	1	2	2	2	2	2	2	2	2	2	2	2	1
TCGA-55-7726	3	2	K-TP53	2	4	1	2	1	2	2	1	2	2	2	2	2	2	2	2	2	2	2	2	2	2	2	2	2	2	2	2	2	2	2	2	2	2	1	2	2	2	2	2	2	2	2	2	2	2	1	2	2	2
TCGA-55-7727	2	2	RAS WT	2	4	1	2	3	2	2	2	2	2	2	2	2	2	2	2	2	2	2	2	2	2	2	2	2	2	2	2	2	2	2	1	1	2	2	1	2	2	2	2	2	1	2	2	2	2	2	2	2	2
TCGA-55-7728	1	2	K-Ox	2	3	2	2	2	2	2	1	2	2	2	2	2	2	2	2	2	2	2	2	2	2	2	1	1	2	2	2	2	2	2	2	2	2	2	2	2	2	2	2	2	2	2	2	2	2	2	2	2	2
TCGA-55-7815	2	2	K-Ox	2	3	2	2	3	1	2	1	2	2	2	2	2	2	2	2	2	2	2	2	2	2	2	2	2	2	2	2	2	2	2	2	2	2	2	1	1	2	2	2	2	2	2	2	2	2	2	2	2	2
TCGA-55-7903	2	2	K-Ox	2	3	1	2	4	2	2	2	2	2	2	2	2	2	2	2	2	1	2	2	2	2	2	2	2	2	2	1	2	2	2	2	1	2	2	2	2	2	2	2	2	2	2	2	2	2	2	2	2	2
TCGA-55-7907	3	2	K-TP53	2	4	1	2	2	2	2	1	2	2	2	2	2	2	2	2	2	2	2	2	2	1	1	2	2	2	2	2	2	2	2	2	2	2	2	1	1	2	2	2	2	1	2	2	2	2	2	2	2	2
TCGA-55-7910	2	2	K-Ox	2	4	1	2	3	2	2	1	2	2	2	2	2	2	2	2	2	2	2	2	1	2	2	2	2	2	2	2	2	2	2	2	2	2	2	2	2	2	2	1	2	1	2	2	2	2	2	2	2	2
TCGA-55-7911	3	2	K-TP53	2	3	1	2	3	2	2	1	2	2	2	2	2	2	2	2	2	2	2	2	2	2	2	2	2	2	2	2	2	1	2	2	2	2	2	2	2	2	2	1	2	2	2	2	2	2	2	2	2	2
TCGA-55-7913	2	2	K-Ox	2	4	1	2	1	2	1	2	2	1	2	2	2	2	2	2	2	1	2	2	2	2	2	2	2	1	2	1	2	2	2	2	2	2	2	2	2	2	2	2	1	2	2	2	2	2	2	2	2	2
TCGA-55-7914	1	2	K-Ox	2	3	1	2	2	2	2	1	2	2	2	2	2	2	2	2	2	2	2	2	2	2	2	2	2	2	2	2	2	1	2	1	2	2	2	2	1	2	2	2	2	2	2	2	2	2	2	2	2	2
TCGA-55-7994	3	2	RAS WT	2	4	1	2	4	2	2	2	2	2	2	2	2	2	2	2	2	2	2	2	1	2	2	2	2	2	1	2	2	2	2	2	2	1	2	2	2	2	2	2	2	2	2	2	2	2	2	1	2	2
TCGA-55-7995	3	2	RAS WT	2	3	1	2	4	1	2	2	2	2	2	2	2	2	2	2	2	1	2	2	2	2	2	2	2	2	2	2	2	2	2	2	2	1	2	2	2	2	2	2	2	2	2	2	2	2	2	1	2	2
TCGA-55-8085	2	2	RAS WT	2	4	1	2	3	1	2	2	2	2	2	2	2	2	2	2	2	2	1	2	2	2	2	2	2	2	2	2	2	2	2	2	2	2	2	2	2	2	2	1	2	2	2	2	2	2	2	2	2	2
TCGA-55-8087	1	1	RAS WT	1	3	2	2	3	2	2	2	2	2	2	2	2	2	2	2	2	2	2	2	2	2	2	2	2	2	2	2	2	2	2	2	2	2	2	2	2	2	2	1	2	2	2	2	2	2	2	2	2	2
TCGA-55-8089	3	2	RAS WT	2	5	1	1	2	2	2	2	2	2	2	2	2	2	2	2	2	2	2	1	2	2	2	2	2	2	2	2	2	2	2	1	2	1	1	2	2	1	2	2	2	2	2	2	2	2	2	1	2	1
TCGA-55-8090	1	2	K-Ox	2	3	2	2	1	2	2	1	2	2	2	2	2	2	2	2	2	2	2	2	2	2	2	2	2	2	2	2	2	2	2	2	2	2	2	2	1	2	1	2	2	2	2	2	2	2	2	1	1	2
TCGA-55-8091	3	2	RAS WT	2	4	2	2	1	2	2	2	2	2	2	2	2	2	2	2	2	2	2	2	2	2	2	2	2	2	2	2	2	1	2	2	2	2	2	2	2	2	2	2	2	2	2	2	2	2	2	2	2	2
TCGA-55-8092	3	2	K-TP53	2	4	1	2	2	2	1	2	2	2	1	2	2	2	1	2	2	2	2	2	2	2	1	2	2	2	1	2	2	2	1	2	2	2	2	2	2	2	2	1	2	2	2	2	2	2	2	2	2	2
TCGA-55-8094	2	2	K-Ox	2	3	1	2	2	1	2	1	2	2	2	2	2	2	2	2	2	1	2	2	2	2	2	2	1	2	2	2	1	2	1	2	2	2	2	2	2	2	2	2	2	2	2	2	2	2	2	2	2	2
TCGA-55-8096	3	1	RAS WT	1	5	1	2	3	2	2	2	2	2	2	2	2	2	2	2	2	2	2	2	2	1	2	2	2	2	2	2	2	2	2	2	2	2	2	2	2	2	2	2	2	2	2	2	2	2	2	2	2	2
TCGA-55-8097	1	1	K-EGFR	2	3	2	2	2	2	2	1	2	2	2	2	2	2	2	2	2	2	2	2	2	2	2	2	2	2	2	2	2	2	2	2	2	2	2	1	2	2	2	2	2	2	2	2	2	2	2	2	2	2
TCGA-55-8203	2	2	K-Ox	2	3	2	2	2	2	1	1	2	2	2	2	2	2	2	2	2	1	2	2	2	2	2	2	2	1	2	2	1	2	2	2	2	2	2	1	2	2	2	2	2	2	2	2	2	2	2	2	2	2
TCGA-55-8204	3	2	RAS WT	2	3	1	2	2	2	2	2	2	2	2	2	2	2	2	2	2	2	2	2	2	2	2	2	2	2	2	2	2	2	2	2	2	1	2	2	2	2	2	1	2	1	2	2	2	2	2	2	2	2
TCGA-55-8205	3	2	RAS WT	2	4	1	1	3	2	2	2	2	2	2	2	2	2	2	2	2	2	2	2	2	2	2	1	2	2	2	2	2	2	2	2	2	1	2	1	2	2	2	2	2	2	2	2	2	2	2	2	2	2
TCGA-55-8206	1	1	RAS WT	1	4	2	2	2	2	2	2	2	2	2	2	2	2	2	2	2	2	2	2	2	2	2	2	2	2	2	2	2	2	2	1	2	2	2	2	2	2	2	2	2	2	2	1	2	2	2	2	2	2
TCGA-55-8207	1	1	K-EGFR	2	3	2	2	2	2	2	1	2	2	2	2	2	2	2	2	2	2	2	2	2	2	2	2	2	2	2	2	2	2	2	2	2	2	2	2	2	2	2	1	2	2	2	2	2	2	2	2	2	2
TCGA-55-8208	3	2	RAS WT	2	3	1	2	3	2	2	2	2	2	2	2	2	2	2	2	2	1	2	2	2	2	2	2	2	2	1	2	2	2	2	2	1	2	2	2	2	2	2	2	1	2	2	2	2	2	2	2	2	2
TCGA-55-8299	3	2	K-TP53	2	3	1	2	3	2	1	1	2	2	2	2	2	2	2	2	2	2	2	2	2	2	2	2	2	1	2	2	2	2	2	2	2	2	2	2	2	2	2	2	2	2	2	2	2	2	2	2	2	2
TCGA-55-8301	3	2	RAS WT	2	4	1	2	4	2	2	2	2	2	2	2	2	2	2	2	2	2	2	2	2	2	2	2	2	2	2	2	2	2	2	2	2	1	2	2	2	2	2	2	2	2	2	2	2	2	2	2	2	2
TCGA-55-8302	3	2	RAS WT	2	4	1	2	3	1	2	2	2	2	2	2	2	2	2	2	2	2	2	2	2	2	2	2	2	2	2	2	2	2	2	2	2	2	2	2	2	2	2	2	2	2	2	2	2	2	2	2	2	2
TCGA-55-8505	2	2	RAS WT	2	4	2	2	1	1	2	2	2	2	2	2	2	2	2	2	2	2	2	2	2	2	2	2	2	2	2	2	2	2	2	2	2	2	2	2	2	2	2	2	2	2	2	2	2	2	2	2	2	2
TCGA-55-8506	3	1	RAS WT	1	3	1	2	2	1	2	2	2	2	2	2	2	2	2	2	2	2	2	2	2	2	1	2	2	2	2	2	2	2	2	2	2	2	2	2	2	1	2	2	1	2	1	2	2	2	2	2	2	2
TCGA-55-8507	2	2	RAS WT	2	1	1	1	2	2	1	2	2	2	2	2	2	2	2	2	1	2	2	2	1	2	2	1	2	1	2	2	2	2	2	2	2	2	2	2	2	2	1	1	2	2	2	2	2	2	1	2	2	2
TCGA-55-8508	2	2	K-Ox	2	3	2	2	3	2	1	1	2	2	2	2	2	2	2	2	2	2	2	2	2	1	2	2	2	2	2	2	2	2	2	2	2	2	2	2	1	2	2	2	2	2	2	2	2	2	2	2	2	2
TCGA-55-8510	1	1	RAS WT	2	4	1	2	2	1	2	2	2	2	2	2	2	2	2	2	2	2	2	2	2	2	2	2	2	2	2	2	2	2	2	2	2	2	2	2	2	2	1	1	2	2	2	2	2	2	2	2	1	2
TCGA-55-8511	3	2	K-TP53	2	4	1	1	3	2	2	2	2	2	2	2	2	2	2	2	2	1	2	2	2	1	2	1	2	2	2	1	2	2	2	2	2	1	2	2	2	1	2	2	2	1	2	2	2	2	2	2	2	2
TCGA-55-8512	1	2	K-Ox	2	3	2	2	2	2	2	1	2	2	2	2	2	2	2	2	2	2	2	2	2	2	2	2	2	2	2	2	2	2	1	2	2	2	2	2	2	2	2	1	2	2	2	2	2	2	2	2	2	2
TCGA-55-8513	1	2	RAS WT	2	3	2	2	3	2	2	2	2	2	2	2	2	2	2	2	2	2	2	2	2	2	2	2	2	2	2	2	2	2	2	2	2	2	2	2	2	2	2	2	2	2	2	2	2	2	2	2	2	2
TCGA-55-8514	1	1	K-EGFR	2	5	1	2	4	2	2	1	2	2	2	2	2	2	2	2	2	1	2	2	2	2	2	2	2	2	2	2	2	2	2	2	2	2	2	2	2	2	2	1	2	2	1	2	2	2	2	2	2	2
TCGA-55-8614	3	2	RAS WT	2	3	1	2	2	2	2	2	2	2	2	2	2	2	2	2	2	2	2	2	2	2	2	2	2	2	2	2	2	2	2	2	2	2	2	2	2	2	2	2	2	2	2	2	2	2	2	2	2	2
TCGA-55-8615	2	2	K-Ox	2	4	2	2	4	2	1	1	2	2	2	2	2	2	2	2	2	2	2	2	2	2	2	2	2	2	2	2	2	2	2	2	2	2	2	2	2	2	2	1	2	2	2	2	2	2	2	2	2	2
TCGA-55-8616	1	1	K-EGFR	2	4	1	1	3	2	2	1	2	2	2	2	2	2	2	2	2	2	2	2	2	2	2	2	2	2	1	2	2	2	2	2	2	2	2	2	2	2	2	1	2	2	2	2	2	1	2	2	2	2
TCGA-55-8619	1	2	RAS WT	2	3	1	1	3	2	2	2	2	2	2	2	2	2	2	2	2	2	2	2	2	2	2	2	2	2	2	2	2	2	2	2	2	2	2	2	2	2	1	2	2	2	2	2	2	2	2	2	2	2
TCGA-55-8620	2	2	RAS WT	2	1	1	1	4	1	2	2	2	2	2	2	2	2	2	2	2	2	2	2	2	2	2	1	2	1	2	2	2	2	2	2	2	2	2	2	2	2	2	1	2	2	2	1	2	1	2	2	2	2
TCGA-55-8621	1	2	RAS WT	2	3	1	2	3	2	2	2	2	2	2	2	2	2	2	2	2	2	2	2	2	2	2	2	2	2	2	2	2	2	2	2	2	2	1	2	2	2	2	1	2	2	2	2	2	2	2	2	2	2
TCGA-55-A48X	1	1	K-EGFR	2	3	1	1	2	2	2	1	2	2	2	2	2	2	2	2	2	2	2	2	2	2	2	2	2	2	2	2	2	2	2	2	2	2	2	2	2	2	2	2	2	2	2	2	2	2	2	2	2	2
TCGA-55-A48Z	3	1	RAS WT	1	5	1	2	4	2	2	2	2	2	2	2	2	2	2	2	2	2	2	2	2	2	1	2	2	2	2	2	2	2	2	2	2	1	2	1	2	2	2	2	2	2	2	2	2	2	2	2	2	2
TCGA-55-A491	3	2	RAS WT	2	4	1	2	3	2	2	2	2	2	2	2	2	2	2	2	2	2	2	2	2	2	2	2	2	2	2	2	2	2	2	2	2	2	2	2	1	2	2	2	2	2	1	2	2	2	2	2	2	2
TCGA-55-A492	1	2	K-Ox	2	4	2	1	2	1	1	1	2	2	2	2	2	2	2	2	2	2	2	2	2	2	2	2	2	2	1	2	2	2	2	2	2	2	2	2	2	2	1	2	2	2	2	2	2	2	2	2	2	2
TCGA-55-A493	3	2	K-TP53	2	4	1	1	2	2	2	1	2	2	2	2	2	2	2	2	2	2	2	2	2	2	2	2	2	2	2	2	2	2	2	2	2	2	2	2	2	2	2	1	2	2	2	2	2	2	2	2	2	2
TCGA-55-A494	2	2	K-Ox	2	4	2	2	3	2	2	1	2	2	2	2	2	2	2	2	2	2	2	2	2	2	2	2	2	1	1	2	2	2	1	2	2	2	2	2	2	2	2	2	2	2	2	2	2	2	2	2	2	2
TCGA-55-A4DF	2	2	RAS WT	2	3	1	2	5	1	2	2	2	2	2	2	2	2	2	2	2	2	2	2	2	2	2	2	2	2	2	2	2	2	2	2	2	2	2	2	2	2	2	1	2	2	2	2	2	2	2	2	2	2
TCGA-55-A4DG	1	1	RAS WT	2	4	1	2	2	2	2	2	2	2	2	2	2	2	2	2	2	2	2	2	2	2	2	2	2	2	2	2	2	2	2	2	2	1	2	2	2	2	2	1	2	2	2	2	2	2	2	1	2	2
TCGA-62-8394	2	1	RAS WT	1	4	1	2	3	2	2	2	2	2	2	2	2	2	2	2	2	2	2	2	2	2	2	2	2	2	2	2	2	2	2	2	2	2	2	2	2	2	2	2	2	2	2	2	2	2	2	2	2	2
TCGA-62-8395	1	1	RAS WT	2	3	2	2	2	2	2	2	2	2	2	2	2	2	2	2	2	2	2	2	2	2	2	2	2	2	2	2	2	2	2	2	2	2	2	2	2	2	2	1	2	2	1	2	2	2	2	2	2	2
TCGA-62-8397	1	1	RAS WT	1	4	2	1	2	2	2	2	2	2	2	2	2	2	2	2	2	2	2	2	2	2	2	2	2	2	2	2	2	2	2	2	2	2	2	2	2	2	2	2	2	2	2	2	2	2	2	2	2	2
TCGA-62-8398	2	2	RAS WT	2	4	2	2	1	1	2	2	2	2	2	2	2	2	2	2	2	2	2	2	2	2	2	2	2	2	2	2	2	2	2	2	2	2	2	2	1	1	2	2	2	2	2	2	2	2	2	2	2	1
TCGA-62-8399	2	2	RAS WT	2	4	1	2	2	2	2	2	2	2	2	2	2	2	2	2	2	2	2	1	2	2	2	2	2	2	2	2	2	2	2	2	2	2	1	2	2	2	2	2	2	2	1	2	2	2	2	2	2	2
TCGA-62-8402	3	2	RAS WT	1	2	1	2	1	2	2	2	2	2	2	2	2	2	2	2	2	2	2	2	2	2	2	2	2	2	2	2	2	2	2	2	2	2	2	2	2	2	2	2	2	1	2	2	2	2	2	2	2	2
TCGA-62-A46O	2	2	RAS WT	2	4	1	2	1	1	2	2	2	2	2	2	2	2	2	2	2	2	2	2	2	2	2	1	1	2	2	2	2	2	1	2	2	2	1	1	2	2	2	2	2	2	2	2	2	2	2	2	2	2
TCGA-62-A46P	2	2	RAS WT	2	4	2	2	3	2	1	2	2	2	2	2	2	2	2	2	2	2	2	2	2	2	2	2	2	2	2	2	2	2	1	2	2	2	2	2	2	2	2	1	2	1	2	2	2	2	2	2	2	2
TCGA-62-A46R	3	2	K-TP53	2	4	1	2	4	2	2	1	2	2	2	2	2	2	2	2	2	2	2	2	2	2	2	1	2	2	2	2	2	2	2	2	2	2	2	2	2	2	2	2	2	2	2	2	2	2	2	2	2	2
TCGA-62-A46S	1	2	K-Ox	2	3	2	2	3	2	1	1	2	2	2	2	2	2	2	2	2	2	2	2	2	2	2	2	2	2	2	2	2	2	2	1	2	2	2	2	2	2	2	2	2	2	2	2	2	2	2	1	2	2
TCGA-62-A46U	3	2	RAS WT	1	3	1	2	3	2	2	2	2	2	2	2	2	2	2	2	2	1	2	2	2	2	2	2	2	2	1	2	2	2	2	2	2	2	2	2	2	2	2	2	2	2	2	2	2	2	2	2	2	2
TCGA-62-A46V	1	1	K-EGFR	2	3	2	2	2	2	2	2	2	1	2	2	2	2	2	2	1	2	2	2	2	2	2	2	2	2	2	2	2	2	2	2	2	2	2	2	2	2	2	2	2	2	2	2	2	2	2	2	2	2
TCGA-62-A46Y	1	1	K-EGFR	2	4	1	2	2	2	2	2	2	2	2	2	2	2	2	2	2	2	2	2	2	2	2	2	1	2	2	2	2	2	2	2	2	2	2	1	2	2	2	1	2	2	2	2	2	2	2	2	2	2
TCGA-62-A470	2	2	RAS WT	2	3	2	2	4	1	2	2	2	2	2	2	2	2	2	2	2	2	2	2	2	2	2	2	2	2	2	2	2	2	2	2	2	2	2	2	2	2	2	2	2	2	2	2	2	2	2	2	2	2
TCGA-62-A471	2	2	RAS WT	2	2	1	2	1	2	1	2	2	2	2	2	2	2	2	2	2	2	2	2	2	2	2	2	1	2	2	2	2	2	2	2	2	2	2	2	2	2	2	2	2	2	2	2	2	2	1	2	2	2
TCGA-62-A472	3	2	K-TP53	2	4	1	2	2	2	2	2	2	1	2	2	2	2	2	2	2	2	2	2	2	2	2	2	2	2	2	2	2	2	2	2	2	2	1	2	2	2	2	2	2	2	2	2	2	2	2	2	2	2
TCGA-64-1676	3	2	RAS WT	2	4	1	2	2	2	2	2	2	2	2	2	2	2	2	2	1	1	2	2	2	2	2	2	2	2	2	1	2	2	2	2	2	2	1	2	2	2	2	1	2	2	2	2	2	2	2	2	2	2
TCGA-64-1677	2	2	K-Ox	2	3	1	2	3	2	2	1	2	2	2	2	2	2	2	1	2	2	2	2	2	2	2	2	2	2	2	2	2	2	2	2	2	2	2	2	2	2	2	1	1	2	2	2	2	2	2	1	2	2
TCGA-64-1678	2	2	RAS WT	2	3	1	2	2	2	2	2	2	2	2	2	2	2	2	NA	NA	NA	NA	NA	NA	NA	NA	NA	NA	NA	NA	NA	NA	NA	NA	NA	NA	NA	NA	NA	NA	NA	NA	NA	NA	NA	NA	NA	NA	NA	NA	NA	NA	NA
TCGA-64-1679	3	1	RAS WT	2	4	1	2	1	2	2	2	2	2	2	2	2	2	2	2	2	2	2	2	2	2	2	2	2	2	2	2	2	2	1	2	2	2	2	2	2	2	2	1	2	2	2	2	2	2	2	2	2	2
TCGA-64-1680	1	2	K-Ox	2	4	2	2	1	2	2	2	2	2	2	2	2	2	2	1	2	2	2	2	2	2	2	2	2	2	2	2	2	2	1	2	2	2	2	2	2	2	2	2	2	2	2	2	2	2	2	2	2	2
TCGA-64-1681	1	1	RAS WT	1	4	1	2	1	2	2	2	2	2	2	2	2	2	2	2	2	2	2	2	2	2	2	2	2	2	2	2	2	2	1	2	2	2	2	1	1	2	2	2	2	2	2	2	2	2	2	2	2	2
TCGA-64-5774	2	2	K-Ox	2	3	2	2	4	2	1	1	2	2	2	2	2	2	2	2	2	2	2	2	2	2	2	2	2	2	2	2	2	2	2	2	1	2	2	2	1	2	2	2	2	2	2	2	2	2	1	2	2	2
TCGA-64-5775	3	2	K-TP53	2	3	1	2	4	2	2	1	2	2	2	2	2	2	2	2	2	2	2	2	2	2	2	2	2	2	2	2	1	2	2	1	2	2	2	2	1	2	2	1	2	2	2	2	2	2	2	2	2	2
TCGA-64-5778	1	2	K-Ox	2	3	1	2	3	2	2	1	2	2	2	2	2	2	2	2	2	2	2	2	2	2	2	1	2	1	2	2	2	2	2	2	2	2	2	2	2	2	2	1	1	2	2	2	2	2	2	2	2	2
TCGA-64-5779	2	2	RAS WT	2	4	2	2	3	2	2	2	2	2	2	2	2	2	2	2	2	2	2	2	2	2	2	2	1	2	1	2	2	2	2	2	2	2	2	2	2	2	2	2	1	2	2	2	2	2	2	2	2	2
TCGA-64-5781	3	2	RAS WT	2	4	2	2	2	1	2	2	2	2	2	2	2	2	2	1	2	2	2	2	2	2	2	1	1	2	2	2	2	2	2	2	2	2	2	2	2	2	2	2	2	2	1	2	2	2	2	2	2	1
TCGA-64-5815	3	2	K-TP53	2	3	2	2	1	2	2	1	2	2	2	2	2	2	2	2	2	2	2	2	1	2	2	2	2	2	2	2	2	2	2	2	2	2	2	2	2	2	2	2	2	2	2	2	2	2	2	2	2	2
TCGA-67-3770	1	1	RAS WT	1	4	1	2	1	2	2	2	2	2	2	2	2	2	2	2	2	2	2	2	2	2	2	2	2	1	2	2	2	2	2	2	2	2	2	1	2	2	2	2	2	2	2	2	2	2	2	2	2	2
TCGA-67-3771	2	2	RAS WT	2	4	1	1	2	1	2	2	2	2	2	2	2	2	2	1	2	2	2	1	2	2	2	1	2	2	1	2	2	2	2	2	2	2	1	2	1	2	2	1	2	2	2	2	2	2	2	2	2	2
TCGA-67-3772	1	1	RAS WT	1	4	2	2	1	2	2	2	2	2	2	2	2	2	2	2	2	2	2	2	2	2	2	2	2	2	2	2	2	2	2	2	2	2	2	2	2	2	1	2	2	2	2	2	2	2	2	2	2	2
TCGA-67-3773	1	2	K-Ox	2	3	2	2	3	2	2	1	2	2	2	2	2	2	2	2	2	2	2	2	2	2	2	2	2	1	2	2	2	2	2	2	2	2	2	2	2	2	2	2	1	2	2	2	2	2	2	2	2	2
TCGA-67-3774	1	2	K-Ox	2	2	2	2	2	1	1	1	2	2	2	2	2	2	2	2	2	2	2	2	2	1	2	2	2	2	2	2	2	2	2	2	2	2	2	2	2	2	2	2	2	2	2	2	2	2	2	2	2	2
TCGA-67-4679	1	1	K-EGFR	2	3	2	2	3	2	2	1	2	2	2	2	2	2	2	2	2	2	2	2	2	2	2	1	2	2	2	2	2	2	1	2	2	2	2	2	2	2	2	1	2	2	2	2	2	2	2	2	2	2
TCGA-67-6215	1	2	K-Ox	2	3	2	2	3	2	2	2	2	2	2	2	1	2	2	2	2	2	2	2	2	2	2	2	2	2	2	2	2	2	2	2	2	2	2	1	2	2	2	2	2	2	2	2	2	2	2	2	2	2
TCGA-67-6216	1	2	K-Ox	2	3	2	2	2	2	2	2	2	2	2	2	1	2	2	2	2	2	2	2	2	2	2	2	2	2	2	2	2	2	2	2	2	2	2	2	2	2	2	2	2	1	2	2	2	2	2	2	2	2
TCGA-67-6217	1	1	RAS WT	1	4	2	2	2	2	2	2	2	2	2	2	2	2	2	2	2	2	2	2	2	2	2	2	2	2	2	2	2	2	2	2	2	2	2	2	2	2	2	2	2	2	2	2	2	2	2	2	2	2
TCGA-69-7760	2	2	RAS WT	1	5	1	2	2	2	2	2	2	2	2	2	2	2	2	2	2	2	2	2	2	2	2	2	2	2	2	2	2	2	2	2	2	2	2	2	2	2	2	2	2	2	2	2	2	2	2	2	2	2
TCGA-69-7761	3	2	RAS WT	2	3	2	2	1	2	2	2	2	2	2	2	2	2	2	2	2	2	2	2	2	2	2	2	2	2	2	2	2	2	2	2	2	2	2	2	2	2	2	2	2	2	2	2	2	2	2	2	2	2
TCGA-69-7763	1	2	K-Ox	2	1	2	2	2	2	1	1	2	2	2	2	2	2	2	2	2	2	2	2	2	2	2	2	2	1	2	2	2	2	2	2	2	2	2	2	2	2	2	1	1	2	2	2	2	2	2	2	2	2
TCGA-69-7764	1	2	RAS WT	2	4	2	2	2	2	2	2	2	2	2	2	2	2	2	2	2	2	2	2	2	2	2	2	2	2	1	2	2	2	2	2	2	1	2	2	2	2	2	1	2	2	2	2	2	2	2	2	2	2
TCGA-69-7765	3	2	K-TP53	2	4	1	2	3	2	2	2	2	2	2	1	2	2	2	2	2	2	2	2	2	2	2	2	2	1	1	2	2	2	1	2	2	1	2	2	2	2	2	1	2	1	2	2	2	2	2	2	2	2
TCGA-69-7973	2	2	K-Ox	2	3	2	2	4	2	2	1	2	2	2	2	2	2	2	2	2	2	2	2	2	2	2	2	2	2	2	2	2	2	2	2	2	2	2	2	1	2	2	2	2	2	2	2	2	2	2	2	2	2
TCGA-69-7974	3	2	K-TP53	2	4	1	2	2	2	2	1	2	2	2	2	2	2	2	2	2	2	2	2	2	2	2	1	1	2	2	2	2	2	2	2	2	2	2	2	2	2	2	1	1	2	1	2	2	1	2	1	2	2
TCGA-69-7978	3	2	K-TP53	2	4	1	2	3	2	2	1	2	2	2	2	2	2	2	2	2	1	2	2	1	2	2	1	2	2	1	2	2	2	1	2	2	2	2	2	2	2	2	1	2	2	2	2	2	2	2	2	2	1
TCGA-69-7979	2	2	RAS WT	2	4	1	2	4	2	2	2	2	2	2	2	2	2	2	2	2	2	2	2	2	2	2	2	1	1	2	2	2	2	2	2	2	1	2	1	2	2	2	2	1	2	2	2	2	2	2	2	2	2
TCGA-69-7980	1	2	K-Ox	2	4	1	2	1	2	2	1	2	2	2	2	2	2	2	2	2	2	2	2	2	2	2	2	2	2	2	2	2	2	2	2	2	2	2	2	2	2	2	2	2	2	1	1	2	2	2	2	2	2
TCGA-69-8253	1	2	K-Ox	2	3	2	2	4	1	2	1	2	2	2	2	2	2	2	2	2	2	2	2	2	2	2	1	2	2	2	2	2	1	2	2	2	2	1	2	2	2	2	2	2	2	2	2	2	2	2	2	2	2
TCGA-69-8254	1	2	K-Ox	2	3	2	2	3	2	2	1	2	2	2	2	2	2	2	2	2	2	2	2	2	2	2	2	2	2	2	2	2	2	2	2	2	2	2	1	2	2	2	1	2	2	2	2	2	2	2	2	2	2
TCGA-69-8255	2	2	RAS WT	2	4	1	2	2	2	2	2	2	2	2	2	2	2	2	2	2	2	2	2	2	2	2	2	2	2	2	2	2	2	2	2	2	1	1	2	2	2	2	1	2	2	2	2	2	2	1	2	2	2
TCGA-69-A59K	3	2	RAS WT	2	4	1	2	5	2	2	2	2	2	2	2	2	2	2	2	2	2	2	2	2	2	2	1	2	2	2	2	2	2	2	2	2	2	2	2	2	2	2	1	1	2	2	2	2	2	2	2	2	2
TCGA-71-6725	1	1	RAS WT	1	4	2	2	3	2	2	2	2	2	2	2	2	2	2	2	2	2	2	2	2	2	2	2	2	2	2	2	2	2	2	2	2	2	2	2	2	2	2	2	2	2	2	2	2	2	2	2	2	2
TCGA-71-8520	3	1	RAS WT	2	3	1	2	3	2	2	2	2	2	2	2	2	2	2	NA	NA	NA	NA	NA	NA	NA	NA	NA	NA	NA	NA	NA	NA	NA	NA	NA	NA	NA	NA	NA	NA	NA	NA	NA	NA	NA	NA	NA	NA	NA	NA	NA	NA	NA
TCGA-73-4658	3	2	RAS WT	2	4	2	1	3	2	2	2	2	2	2	2	2	2	2	2	2	2	2	2	2	2	2	1	2	2	2	2	2	2	2	2	2	1	2	2	2	2	2	1	2	1	2	1	2	2	2	2	2	2
TCGA-73-4659	2	2	K-Ox	2	4	2	2	4	2	1	1	2	2	2	2	2	2	2	2	2	2	2	2	2	2	2	2	2	2	1	2	2	2	2	2	2	2	2	2	1	2	1	2	2	2	2	2	2	2	2	2	2	2
TCGA-73-4662	1	2	K-Ox	2	4	2	2	2	2	2	1	2	2	2	2	2	2	2	2	2	2	2	2	2	2	2	2	2	2	1	2	2	2	2	2	2	2	2	2	2	2	2	1	2	2	2	2	2	2	2	2	2	2
TCGA-73-4666	3	2	RAS WT	2	3	1	2	4	1	2	2	2	2	2	2	2	2	2	2	2	2	2	2	2	2	2	2	1	2	2	2	2	2	2	2	2	2	2	2	2	2	2	1	1	2	1	2	2	2	2	2	2	2
TCGA-73-4668	3	2	K-TP53	2	4	2	2	1	2	2	1	2	2	2	2	2	2	2	2	2	2	2	2	2	2	2	2	1	2	2	2	2	2	2	2	2	2	2	2	2	2	2	1	1	2	2	2	2	2	2	2	2	2
TCGA-73-4670	2	2	K-Ox	2	4	1	2	1	1	1	1	2	2	2	2	2	2	2	2	2	2	2	2	2	1	2	2	2	2	2	1	1	2	2	2	2	2	2	2	2	2	2	2	2	2	2	2	2	2	2	2	2	2
TCGA-73-4675	2	2	RAS WT	2	4	2	2	2	2	2	2	2	2	2	2	2	2	2	2	2	1	2	2	2	2	2	2	2	2	2	2	2	2	2	2	2	2	2	2	2	2	2	2	2	2	2	2	2	2	2	2	2	2
TCGA-73-4676	3	2	RAS WT	2	4	1	2	3	1	2	2	2	2	2	2	2	2	2	2	2	1	1	2	2	2	2	2	2	2	1	2	2	2	2	2	2	1	2	2	2	2	2	2	2	1	2	2	2	2	2	2	2	2
TCGA-73-4677	1	2	K-Ox	2	3	2	2	3	1	1	1	2	2	2	2	2	2	2	2	2	1	2	2	2	2	2	2	2	2	2	1	2	2	2	2	2	2	2	2	2	2	2	2	2	2	2	2	2	2	2	2	2	2
TCGA-73-7498	1	1	K-EGFR	2	3	2	2	3	1	1	1	2	2	2	2	2	2	2	2	2	2	2	2	2	2	2	2	2	2	2	2	2	2	2	2	2	2	2	1	2	2	2	2	2	2	2	2	2	2	2	2	2	2
TCGA-73-7499	3	1	RAS WT	2	4	1	2	2	2	2	2	2	2	2	1	2	2	2	1	2	1	2	2	2	2	2	2	2	2	2	2	2	2	2	2	1	2	2	2	2	2	2	2	2	2	2	2	2	2	2	2	2	2
TCGA-75-5122	3	2	K-TP53	2	4	2	2	2	2	2	2	2	2	2	2	2	1	2	2	2	2	2	2	2	1	2	2	2	2	2	2	2	2	2	2	2	2	2	2	2	2	2	2	2	2	2	2	2	2	2	2	2	2
TCGA-75-5125	3	2	RAS WT	2	4	1	2	1	2	2	2	2	2	2	2	2	2	2	2	2	2	2	2	2	2	2	2	2	2	2	1	2	2	2	2	2	2	2	2	2	2	2	1	2	2	2	2	2	2	2	2	2	2
TCGA-75-5126	3	2	K-TP53	2	4	2	2	4	2	2	1	2	2	2	2	2	2	2	2	2	2	2	2	2	2	2	2	2	2	2	2	2	2	2	2	2	2	2	2	2	2	2	1	2	1	2	2	2	2	2	2	2	2
TCGA-75-5146	1	2	K-Ox	2	2	1	2	3	2	1	2	2	2	1	2	2	2	2	2	2	2	2	2	2	2	2	2	2	2	2	2	2	2	2	2	2	2	2	2	2	2	2	2	2	2	2	2	2	2	2	2	2	2
TCGA-75-5147	2	1	RAS WT	2	3	1	2	3	2	2	2	2	2	2	2	2	2	2	2	2	2	2	2	2	2	2	2	2	2	2	2	2	2	2	2	2	2	2	2	2	2	2	2	2	2	2	2	2	2	2	2	2	2
TCGA-75-6203	1	2	RAS WT	2	4	2	2	3	2	2	2	2	2	2	2	2	2	2	2	2	2	2	2	2	2	2	2	2	2	2	2	2	2	2	2	2	2	2	2	2	2	2	2	2	2	2	2	2	2	2	2	2	2
TCGA-75-6205	3	2	K-TP53	2	4	2	2	2	2	2	2	2	2	2	2	2	2	2	2	2	2	2	2	2	2	2	2	2	2	2	2	2	2	2	2	2	2	2	2	2	2	2	1	2	2	2	2	2	2	2	2	2	2
TCGA-75-6206	1	2	K-Ox	2	3	2	2	3	1	1	1	2	2	2	2	2	2	2	2	2	2	2	2	2	2	2	2	2	2	2	2	2	2	2	2	2	2	2	2	2	2	2	1	2	2	2	2	2	2	2	2	2	1
TCGA-75-6207	3	2	RAS WT	1	4	1	2	2	2	2	2	2	2	2	2	2	2	2	2	2	2	2	2	2	2	2	2	2	2	2	2	2	2	2	2	2	2	2	1	2	1	2	2	2	2	2	2	2	2	2	2	2	2

TCGA-75-6211	2	2	RAS WT	2	5	1	2	2	1	2	2	2	2	2	2	2	2	2	2	1	2	2	2	2	2
TCGA-75-6212	1	1	RAS WT	1	3	2	2	2	2	2	2	2	2	2	2	2	2	2	2	2	2	2	2	2	2
TCGA-75-6214	2	2	RAS WT	2	5	1	2	2	2	2	2	2	2	2	2	2	2	2	2	2	2	2	2	2	2
TCGA-75-7025	1	1	RAS WT	1	4	2	2	2	2	2	2	2	2	2	2	2	2	2	2	2	2	2	2	2	2
TCGA-75-7027	2	2	K-Ox	2	3	2	2	1	1	2	1	2	2	2	2	2	2	2	2	2	2	2	2	2	2
TCGA-75-7030	1	2	K-Ox	2	3	2	2	3	2	2	1	2	2	2	2	2	2	2	2	2	2	2	2	2	2
TCGA-75-7031	2	2	RAS WT	2	3	1	2	2	2	1	2	2	2	2	2	2	2	2	2	2	2	2	2	2	2
TCGA-78-7145	3	2	K-TP53	2	4	1	2	1	2	2	1	2	2	2	2	2	2	2	2	2	2	2	2	2	2
TCGA-78-7146	3	2	RAS WT	2	4	1	2	2	1	2	2	2	2	2	2	2	2	2	1	2	1	2	2	2	2
TCGA-78-7147	1	1	RAS WT	1	4	1	2	2	2	2	2	2	2	2	2	2	2	2	2	2	2	2	2	2	2
TCGA-78-7148	2	2	K-Ox	2	3	2	1	2	2	1	1	2	2	2	2	2	2	2	2	2	2	2	2	2	2
TCGA-78-7149	2	2	RAS WT	2	3	1	2	2	1	2	2	2	2	2	2	2	2	2	2	2	1	2	2	2	2
TCGA-78-7150	2	2	RAS WT	2	4	1	2	1	2	2	2	2	2	2	2	2	2	2	2	2	2	2	2	2	2
TCGA-78-7152	1	2	RAS WT	2	3	2	1	3	2	1	2	2	2	2	2	2	2	2	2	2	2	2	1	2	2
TCGA-78-7153	2	2	K-Ox	2	3	2	1	3	1	2	2	2	2	1	2	2	2	2	1	2	2	2	2	2	2
TCGA-78-7154	2	2	RAS WT	2	4	1	2	3	1	2	2	2	2	2	2	2	2	2	2	2	2	2	2	2	2
TCGA-78-7155	2	2	RAS WT	2	5	1	2	3	2	2	2	2	2	2	2	2	2	2	2	2	2	2	2	2	2
TCGA-78-7156	2	2	RAS WT	2	3	2	2	1	1	1	2	2	2	2	2	2	2	2	2	2	2	2	2	2	2
TCGA-78-7158	1	2	RAS WT	2	3	2	2	3	1	2	2	2	2	2	2	2	2	2	2	2	2	2	2	2	2
TCGA-78-7159	2	2	K-Ox	2	3	2	1	3	2	1	1	2	2	2	2	2	2	2	2	2	2	2	2	2	2
TCGA-78-7160	2	2	K-Ox	2	4	2	2	2	2	1	1	2	2	2	2	2	2	2	2	2	1	2	2	2	2
TCGA-78-7161	2	2	K-Ox	2	4	2	2	2	2	1	1	2	2	2	2	2	2	2	2	2	1	2	2	2	2
TCGA-78-7162	1	2	RAS WT	2	3	2	2	3	1	2	2	2	2	2	2	2	2	2	2	2	2	2	2	2	2
TCGA-78-7163	1	1	K-EGFR	2	4	2	2	4	2	2	2	2	2	2	2	1	2	2	2	2	2	2	2	2	2
TCGA-78-7166	2	2	K-Ox	2	3	2	2	1	1	1	1	2	2	2	2	2	2	2	2	2	2	2	2	2	2
TCGA-78-7167	1	2	K-Ox	2	2	2	2	2	1	1	1	2	2	2	2	2	2	2	2	2	2	2	2	2	2
TCGA-78-7220	2	2	K-Ox	2	1	1	2	1	1	1	2	2	2	2	2	2	1	2	2	2	2	2	2	2	1
TCGA-78-7535	1	1	RAS WT	2	4	1	2	3	2	1	2	2	2	2	2	2	2	2	2	2	2	2	2	2	2
TCGA-78-7536	2	2	RAS WT	2	3	1	2	3	2	1	2	2	2	2	2	2	2	2	2	2	2	2	2	1	1
TCGA-78-7537	1	2	K-Ox	2	3	2	1	3	2	1	2	2	2	1	2	2	2	2	2	2	2	2	2	2	2
TCGA-78-7539	1	1	K-EGFR	2	3	2	2	3	2	2	1	2	2	2	2	2	2	2	2	2	1	1	2	2	2
TCGA-78-7540	1	2	K-Ox	2	4	1	2	1	2	2	1	2	2	2	2	2	2	2	2	2	2	2	2	2	2
TCGA-78-7542	3	2	RAS WT	2	3	1	2	1	2	2	2	2	2	2	2	2	2	2	2	2	2	2	2	2	2
TCGA-78-7633	2	2	K-Ox	2	3	2	2	2	1	2	2	2	2	1	2	2	2	2	2	2	2	2	2	2	2
TCGA-78-8640	2	2	RAS WT	2	3	1	2	2	2	2	2	2	2	2	2	2	2	2	2	2	2	2	2	2	2
TCGA-78-8648	3	2	RAS WT	2	3	2	2	3	2	2	2	2	2	2	2	2	2	2	2	2	2	2	2	2	2
TCGA-78-8655	1	2	K-Ox	2	4	2	2	1	2	2	1	2	2	2	2	2	2	2	2	2	2	2	2	2	2
TCGA-78-8660	3	2	RAS WT	2	3	1	2	2	2	2	2	2	2	2	2	2	2	2	2	1	2	2	2	2	2
TCGA-78-8662	2	2	K-Ox	2	4	1	2	3	2	2	2	2	2	1	2	2	2	2	2	2	2	1	2	2	2
TCGA-80-5607	1	2	K-Ox	2	3	2	2	3	2	2	2	2	2	1	2	2	2	2	1	2	2	2	2	2	2
TCGA-80-5608	2	2	K-Ox	2	3	2	2	4	2	1	1	2	2	2	2	2	2	2	2	2	2	2	2	2	2
TCGA-80-5611	3	2	RAS WT	2	4	2	2	2	2	2	2	2	2	2	2	2	2	2	2	2	1	2	2	2	2
TCGA-83-5908	3	2	K-TP53	2	4	1	2	3	2	2	1	2	2	2	2	2	2	2	2	2	2	2	2	2	2
TCGA-86-6562	3	2	K-TP53	2	4	2	2	1	2	2	2	2	2	2	1	2	2	2	2	2	1	2	2	2	2
TCGA-86-6851	3	2	K-TP53	2	3	1	2	1	2	2	1	2	2	2	2	2	2	2	2	2	2	2	2	2	2
TCGA-86-7701	2	2	K-Ox	2	4	1	2	3	2	1	1	2	2	2	2	2	2	2	1	2	2	2	2	2	1
TCGA-86-7711	3	2	RAS WT	2	2	1	2	2	2	2	2	2	2	2	2	2	2	2	2	2	2	2	2	1	2
TCGA-86-7713	2	2	K-Ox	2	4	2	2	2	2	1	1	2	2	2	2	2	2	2	2	2	2	2	2	2	2
TCGA-86-7714	1	2	K-Ox	2	3	2	2	3	2	2	2	2	2	1	2	2	2	2	1	2	2	2	2	2	2
TCGA-86-7953	3	2	K-TP53	2	4	2	2	4	2	2	1	2	2	2	2	2	2	2	2	2	2	2	2	2	2
TCGA-86-7954	3	1	RAS WT	2	3	1	2	2	2	2	2	2	2	2	2	2	2	2	2	2	2	2	2	2	2
TCGA-86-7955	2	2	RAS WT	2	4	2	1	2	2	2	2	2	2	2	2	2	2	2	2	2	2	1	2	2	2
TCGA-86-8054	2	2	K-Ox	2	3	2	2	2	2	1	1	2	2	2	2	2	2	2	2	2	1	2	2	2	2
TCGA-86-8055	3	1	RAS WT	1	5	1	2	1	2	2	2	2	2	2	2	2	2	2	2	2	2	2	2	2	2
TCGA-86-8056	1	2	K-Ox	2	3	1	2	2	2	2	1	2	2	2	2	2	2	2	2	2	1	2	2	2	2
TCGA-86-8073	2	2	RAS WT	2	4	1	2	3	2	2	2	2	2	2	2	2	2	2	2	2	2	2	2	2	2
TCGA-86-8074	3	1	RAS WT	1	5	1	1	2	2	2	2	2	2	2	2	2	2	2	2	2	1	2	2	2	2
TCGA-86-8075	3	1	RAS WT	1	4	1	2	2	2	2	2	2	2	2	2	2	2	2	2	2	2	2	2	2	2
TCGA-86-8076	1	2	K-Ox	2	3	2	2	3	2	2	1	2	2	2	2	2	2	2	2	2	2	2	2	2	2
TCGA-86-8278	3	1	RAS WT	2	4	2	2	3	2	2	2	2	2	2	2	2	2	1	2	2	2	2	2	2	2
TCGA-86-8279	3	1	RAS WT	2	4	1	2	2	2	2	2	2	2	2	2	2	2	2	2	2	2	2	2	1	2
TCGA-86-8280	1	1	RAS WT	1	4	1	2	1	2	1	2	2	2	2	2	2	2	2	2	2	2	2	2	2	2
TCGA-86-8281	2	2	K-Ox	2	3	2	2	3	1	2	1	2	2	2	2	2	2	2	2	2	2	2	2	2	2
TCGA-86-8358	2	2	RAS WT	2	2	1	1	2	1	2	2	2	2	2	2	2	2	2	2	2	2	2	2	2	1
TCGA-86-8359	2	2	K-Ox	2	4	2	1	3	2	1	1	2	2	2	2	2	2	2	2	2	2	2	2	2	2
TCGA-86-8585	3	2	RAS WT	2	3	1	1	2	2	2	2	2	2	2	2	2	2	2	2	2	2	1	2	2	2
TCGA-86-8668	1	1	RAS WT	1	4	2	2	1	2	2	2	2	2	2	2	2	2	2	2	2	2	2	2	2	2
TCGA-86-8669	2	2	RAS WT	2	3	2	2	4	2	2	2	2	2	2	2	2	2	2	2	2	2	2	2	2	2
TCGA-86-8671	1	1	RAS WT	1	3	2	2	3	2	2	2	2	2	2	2	2	2	2	2	2	2	2	2	2	2
TCGA-86-8672	3	2	RAS WT	2	2	1	1	2	2	2	2	2	2	2	2	2	2	2	2	2	2	2	2	2	2
TCGA-86-8673	2	2	RAS WT	2	4	1	2	3	2	2	2	2	2	2	2	2	2	2	2	2	2	2	2	2	2
TCGA-86-8674	2	2	K-Ox	2	3	2	2	3	1	2	1	2	2	2	2	2	2	2	2	2	2	2	2	2	2
TCGA-86-A456	1	1	K-EGFR	2	4	2	2	2	2	2	1	2	2	2	2	2	2	2	2	2	2	2	2	2	2
TCGA-86-A4D0	2	2	RAS WT	2	2	1	1	3	1	1	2	2	2	2	2	2	2	2	NA	NA	NA	NA	NA	NA	NA
TCGA-86-A4JF	3	2	K-TP53	2	3	1	2	3	2	1	1	2	2	2	2	2	2	2	2	2	1	2	2	2	2
TCGA-86-A4P7	1	1	RAS WT	1	4	1	2	3	2	2	2	2	2	2	2	2	2	2	2	2	2	2	2	2	2
TCGA-86-A4P8	1	2	K-Ox	2	3	2	2	3	2	2	2	2	2	2	2	1	2	2	2	2	2	2	2	2	2
TCGA-91-6828	2	2	K-Ox	2	2	2	2	4	2	2	1	2	2	2	2	2	2	2	2	2	2	2	2	2	2
TCGA-91-6829	3	2	RAS WT	2	3	1	2	2	2	2	2	2	2	2	2	2	2	2	2	2	2	2	2	2	2
TCGA-91-6830	1	2	RAS WT	2	3	2	2	2	2	2	2	2	2	2	2	2	2	2	2	2	2	2	2	2	2
TCGA-91-6831	2	2	RAS WT	2	4	1	2	1	2	2	2	2	2	2	2	2	2	2	2	2	2	2	2	2	2
TCGA-91-6835	1	2	RAS WT	1	4	2	2	2	2	2	2	2	2	2	2	2	2	2	2	2	1	2	2	2	2
TCGA-91-6836	3	2	K-TP53	2	3	1	2	3	2	2	1	2	2	2	2	2	2	2	2	2	1	2	2	2	2
TCGA-91-6840	2	2	RAS WT	2	2	1	2	2	2	2	2	2	2	2	2	2	2	2	2	2	2	2	2	2	2
TCGA-91-6847	2	2	RAS WT	1	5	1	2	2	2	2	2	2	2	2	2	2	2	2	2	2	2	2	2	2	2
TCGA-91-6848	3	2	RAS WT	2	4	1	2	2	1	2	2	2	2	2	2	2	2	2	2	2	2	2	2	2	2
TCGA-91-6849	1	2	K-Ox	2	3	2	2	2	1	1	1	2	2	2	2	2	2	2	2	2	2	2	2	2	2
TCGA-91-7771	1	2	RAS WT	2	3	2	2	3	2	2	2	2	2	2	2	2	2	2	2	1	2	2	2	2	2
TCGA-91-8496	1	1	RAS WT	1	4	2	1	3	2	2	2	2	2	2	2	2	2	2	2	2	2	1	2	2	2
TCGA-91-8497	1	1	K-EGFR	2	3	2	2	1	2	2	1	2	2	2	2	2	2	2	2	2	2	2	2	2	2
TCGA-91-8499	3	2	RAS WT	2	2	1	2	2	2	2	2	2	2	2	2	2	2	2	2	2	2	2	2	2	2
TCGA-91-A4BC	3	2	RAS WT	2	4	1	2	3	2	2	2	2	2	2	2	2	2	2	2	1	2	2	2	2	2
TCGA-91-A4BD	1	2	K-Ox	2	3	2	2	3	2	2	2	2	2	1	2	2	2	2	2	2	2	2	2	2	2
TCGA-93-7347	1	2	K-Ox	2	3	2	2	3	2	2	1	2	2	2	2	2	2	2	2	2	2	2	2	2	2
TCGA-93-7348	1	1	K-EGFR	2	4	2	2	1	2	2	2	2	2	2	2	2	1	2	1	2	2	2	2	2	2
TCGA-93-8067	2	2	K-Ox	2	2	1	1	1	2	2	1	2	2	2	2	2	2	2	2	2	2	2	2	2	2
TCGA-93-A4JN	3	1	RAS WT	2	3	1	2	2	2	2	2	2	2	2	2	2	1	2	2	2	2	2	2	2	2
TCGA-93-A4JO	1	2	K-Ox	2	4	1	2	3	2	2	1	2	2	2	2	2	2	2	2	2	2	2	2	2	2
TCGA-93-A4JP	1	1	RAS WT	1	4	1	2	3	2	2	2	2	2	2	2	2	2	2	2	2	2	2	2	2	2
TCGA-93-A4JQ	3	2	K-TP53	2	3	2	2	2	2	2	2	2	2	2	2	2	1	2	2	2	2	2	2	2	2
TCGA-95-7039	3	2	K-TP53	2	4	1	2	4	2	2	1	2	2	2	2	2	2	2	2	2	2	2	2	2	2
TCGA-95-7043	2	2	RAS WT	2	3	1	2	3	1	2	2	2	2	2	2	2	2	2	2	2	2	2	2	1	2
TCGA-95-7562	3	2	K-TP53	2	4	1	2	2	2	2	1	2	2	2	2	2	2	2	1	2	2	2	2	2	1
TCGA-95-7567	3	2	K-TP53	2	2	1	2	2	2	2	1	2	2	2	2	2	2	2	2	2	2	2	2	2	2
TCGA-95-7944	3	2	RAS WT	2	4	1	2	3	2	2	2	2	2	2	2	2	2	2	2	2	2	2	2	2	2
TCGA-95-7947	2	2	RAS WT	2	4	1	2	4	1	2	2	2	2	2	2	2	2	2	1	2	2	2	2	2	2
TCGA-95-7948	3	1	RAS WT	2	3	2	2	4	2	2	2	2	2	2	2	2	2	2	1	2	2	2	1	2	2
TCGA-95-8039	1	1	K-EGFR	2	3	1	1	1	2	2	2	2	2	2	2	2	1	2	2	2	2	2	2	2	2
TCGA-95-8494	3	2	RAS WT	2	4	1	2	2	2	2	2	2	2	2	2	2	2	2	2	2	2	2	2	2	2
TCGA-95-A4VK	2	2	K-Ox	2	3	2	2	1	2	2	1	2	2	2	2	2	2	2	2	2	2	2	2	2	2
TCGA-95-A4VN	3	2	K-TP53	2	4	2	2	4	2	2	1	2	2	2	2	2	2	2	2	2	2	2	2	2	2
TCGA-95-A4VP	2	2	K-Ox	2	2	2	2	2	2	1	1	2	2	2	2	2	2	2	2	2	2	2	2	2	2
TCGA-97-7546	1	1	RAS WT	2	3	2	1	3	2	2	2	2	2	2	2	2	2	2	2	2	2	2	2	2	2
TCGA-97-7547	1	2	RAS WT	2	3	2	2	2	2	2	2	2	2	2	2	2	2	2	2	2	2	2	2	2	2
TCGA-97-7552	1	2	K-Ox	2	3	2	2	1	2	2	1	2	2	2	2	2	2	2	2	2	2	2	2	2	2
TCGA-97-7553	1	2	RAS WT	2	4	1	2	4	2	2	2	2	2	2	2	2	2	2	2	2	2	2	2	2	2
TCGA-97-7554	3	2	K-TP53	2	4	1	2	4	2	2	1	2	2	2	2	2	2	2	2	2	2	2	2	2	2
TCGA-97-7937	3	1	RAS WT	2	4	1	2	2	2	2	2	2	2	2	2	2	2	2	2	2	2	2	2	2	2
TCGA-97-7938	1	2	K-Ox	2	3	1	2	2	2	2	1	2	2	2	2	2	2	2	2	2	2	2	2	2	2
TCGA-97-7941	1	2	K-Ox	2	3	2	2	3	2	2	1	2	2	2	2	2	2	2	2	2	2	2	2	2	2
TCGA-97-8171	1	1	RAS WT	1	4	2	2	3	2	2	2	2	2	2	2	2	2	2	2	2	2	2	2	2	2
TCGA-97-8172	1	2	K-Ox	2	3	1	2	3	2	2	1	2	2	2	2	2	2	2	2	2	2	2	2	2	2
TCGA-97-8174	1	2	RAS WT	2	2	2	2	2	2	2	2	2	2	2	2	2	2	2	2	2	2	2	2	2	2
TCGA-97-8175	3	2	K-TP53	2	4	1	2	1	2	2	2	2	2	2	1	2	2	2	2	2	2	2	2	2	2
TCGA-97-8176	2	2	K-Ox	2	4	2	2	2	1	2	1	2	2	2	2	2	2	2	2	2	2	2	2	2	2
TCGA-97-8177	1	1	RAS WT	1	4	2	2	2	2	2	2	2	2	2	2	2	2	2	1	2	2	2	2	2	2
TCGA-97-8179	2	2	K-Ox	2	4	1	2	2	1	1	1	2	2	2	2	2	2	2	2	2	2	2	2	2	2
TCGA-97-8547	3	1	RAS WT	1	4	1	2	2	2	2	2	2	2	2	2	2	2	2	2	2	2	2	2	2	2
TCGA-97-8552	1	1	RAS WT	1	4	2	1	2	2	2	2	2	2	2	2	2	2	2	1	2	2	2	2	2	2
TCGA-97-A4LX	3	2	K-TP53	2	3	1	2	3	2	2	1	2	2	2	2	2	2	2	2	2	2	2	2	2	2
TCGA-97-A4M0	1	1	K-EGFR	2	3	1	2	2	2	2	1	2	2	2	2	2	2	2	2	2	2	2	2	2	2
TCGA-97-A4M1	1	1	RAS WT	1	4	1	2	2	2	2	2	2	2	2	2	2	2	2	2	2	2	2	2	2	2
TCGA-97-A4M2	1	2	RAS WT	2	3	2	2	3	2	2	2	2	2	2	2	2	2	2	2	2	2	2	2	2	2
TCGA-97-A4M3	2	2	RAS WT	2	3	2	2	3	1	1	2	2	2	2	2	2	2	2	2	2	2	2	1	2	2
TCGA-97-A4M5	1	2	K-Ox	2	2	2	2	3	2	2	1	2	2	2	2	2	2	2	2	2	2	2	2	2	2
TCGA-97-A4M6	1	1	RAS WT	1	3	2	2	1	2	2	2	2	2	2	2	2	2	2	2	2	2	2	2	2	2
TCGA-97-A4M7	1	1	RAS WT	1	4	2	2	2	2	2	2	2	2	1	2	2	2	2	2	2	2	2	2	2	2
TCGA-99-7458	1	2	K-Ox	2	3	1	1	3	2	2	1	2	2	2	2	2	2	2	2	2	2	2	2	2	2
TCGA-99-8025	2	2	K-Ox	2	4	1	2	4	2	1	1	2	2	2	2	2	2	2	2	2	2	2	2	2	2
TCGA-99-8028	3	2	K-TP53	2	3	1	2	3	2	2	1	2	2	2	2	2	2	2	2	2	2	2	2	2	2
TCGA-99-8032	2	2	K-Ox	2	2	2	2	2	2	1	1	2	2	2	2	2	2	2	2	2	2	2	2	2	2
TCGA-99-8033	2	2	K-Ox	2	4	1	2	2	1	2	1	2	2	2	2	2	2	2	2	2	2	2	2	2	2
TCGA-J2-8192	3	1	RAS WT	1	4	2	2	1	2	2	2	2	2	2	2	2	2	2	2	2	2	2	2	2	2
TCGA-J2-8194	2	2	K-Ox	2	4	2	2	3	1	1	1	2	2	2	2	2	2	2	2	2	2	1	2	2	2
TCGA-J2-A4AD	2	1	RAS WT	2	4	1	2	2	2	2	2	2	2	2	2	2	2	2	2	2	2	2	2	2	2
TCGA-J2-A4AE	1	1	K-EGFR	2	4	2	2	3	2	2	2	2	2	2	2	2	1	2	2	2	1	2	2	2	2
TCGA-J2-A4AG	1	1	K-EGFR	2	3	2	2	2	2	2	1	2	2	2	2	2	2	2	2	2	2	2	2	2	2
TCGA-L4-A4E5	2	1	RAS WT	2	4	1	2	3	2	2	2	2	2	2	2	2	2	2	2	2	2	2	2	2	2
TCGA-L4-A4E6	1	2	RAS WT	2	3	2	2	3	2	2	2	2	2	2	2	2	2	2	2	2	2	2	2	2	2
TCGA-L9-A443	1	2	K-Ox	2	4	2	1	3	2	2	1	2	2	2	2	2	2	2	2	2	2	2	2	2	2
TCGA-L9-A444	3	2	RAS WT	2	5	1	2	4	2	2	2	2	2	2	2	2	2	2	2	2	2	2	2	2	2
TCGA-MN-A4N1	2	1	RAS WT	2	4	1	2	1	2	2	2	2	2	1	2	2	2	2	2	2	2	2	2	2	2
TCGA-MN-A4N4	3	2	RAS WT	2	4	1	2	2	2	2	2	2	2	2	2	2	2	2	2	2	2	2	2	2	2
TCGA-MN-A4N5	3	2	K-TP53	2	4	1	2	2	2	2	1	2	2	2	2	2	2	2	2	2	2	1	2	2	2
TCGA-MP-A4SV	2	2	RAS WT	2	3	1	2	2	2	2	2	2	2	2	2	2	2	2	2	2	1	2	2	2	2

TCGA-75-6211	2	2	2	2	2	1	2	2	2	2	2	2	1	2	2	2	2	2	2	2	2	2	2	2	2	2	2	2	2
TCGA-75-6212	2	2	2	2	2	2	2	2	2	2	2	2	2	2	2	2	2	2	2	2	2	2	2	2	2	2	2	2	2
TCGA-75-6214	2	2	2	2	2	2	2	2	2	2	2	2	2	2	1	2	2	2	1	2	1	2	2	2	2	1	2	2	2
TCGA-75-7025	2	2	2	2	2	2	2	2	2	2	2	2	2	2	2	2	2	2	2	2	1	2	2	2	2	2	2	2	2
TCGA-75-7027	2	2	2	2	2	2	2	2	2	2	2	2	2	2	2	2	2	2	2	2	2	2	2	2	2	2	2	2	2
TCGA-75-7030	2	2	2	2	2	2	2	2	2	2	2	2	2	2	2	2	2	2	2	2	2	2	2	2	2	2	2	2	2
TCGA-75-7031	2	2	2	2	2	2	2	1	2	2	2	2	2	2	2	2	2	2	1	2	2	2	2	2	2	2	2	2	2
TCGA-78-7145	2	2	2	2	2	2	2	2	2	2	1	2	2	2	2	2	2	2	1	2	2	2	2	2	2	2	2	2	2
TCGA-78-7146	2	2	2	2	2	2	2	2	1	2	2	2	1	2	2	2	2	2	2	2	2	2	2	2	2	2	2	2	2
TCGA-78-7147	2	2	2	2	2	2	2	2	2	2	2	1	2	2	2	2	2	2	2	2	2	2	2	2	2	2	2	2	2
TCGA-78-7148	2	2	1	2	2	2	2	2	2	2	2	2	2	1	1	2	2	2	2	2	2	2	2	2	2	2	2	2	2
TCGA-78-7149	2	2	2	2	2	2	1	2	2	2	2	2	2	2	2	2	2	2	2	2	2	2	2	2	2	2	2	2	2
TCGA-78-7150	2	2	2	1	1	2	2	2	2	2	2	2	2	2	2	2	2	2	1	2	1	2	2	2	2	2	2	2	2
TCGA-78-7152	2	2	2	2	2	2	2	2	2	2	2	2	2	2	2	2	2	2	2	2	2	2	2	2	2	2	2	2	2
TCGA-78-7153	2	2	2	2	2	2	2	2	2	2	2	2	2	2	2	2	2	1	2	2	2	2	2	2	2	2	2	2	1
TCGA-78-7154	2	2	2	2	2	2	2	2	1	2	2	2	1	2	2	2	2	2	1	2	2	2	2	2	2	2	2	2	2
TCGA-78-7155	2	2	2	1	2	2	2	1	2	2	2	2	2	2	2	2	2	2	1	2	2	2	1	2	2	1	2	2	2
TCGA-78-7156	2	2	2	2	2	2	2	2	2	2	2	2	2	2	2	2	1	2	2	1	2	1	2	2	2	1	2	2	2
TCGA-78-7158	2	2	2	2	2	2	2	2	1	2	2	2	2	2	1	2	2	2	2	2	2	2	2	2	2	1	2	2	2
TCGA-78-7159	2	2	2	2	2	2	2	2	2	2	2	2	2	2	2	1	2	2	1	2	2	2	2	2	2	2	2	2	2
TCGA-78-7160	2	2	2	2	2	2	1	2	2	2	2	2	1	2	2	2	2	2	2	2	2	2	2	2	2	2	2	2	2
TCGA-78-7161	2	2	2	2	2	2	1	2	2	2	2	2	2	2	2	2	2	2	2	2	2	2	2	2	2	2	2	2	2
TCGA-78-7162	2	2	2	2	2	2	2	2	2	2	2	2	2	2	2	2	2	2	2	2	2	2	2	2	2	2	2	2	2
TCGA-78-7163	2	2	2	2	2	2	1	2	2	2	2	2	2	2	2	2	2	2	2	1	2	2	2	2	2	2	2	2	2
TCGA-78-7166	2	2	2	2	1	2	2	2	2	2	2	2	2	2	1	2	2	2	1	2	2	2	2	2	2	2	2	2	2
TCGA-78-7167	2	2	2	2	2	2	2	2	2	2	2	2	2	2	2	2	1	2	2	2	2	2	2	2	2	2	2	2	2
TCGA-78-7220	1	2	2	1	2	2	2	2	2	2	2	2	2	2	1	2	2	1	1	1	2	1	2	2	2	1	2	1	2
TCGA-78-7535	2	2	2	2	2	2	2	2	2	2	2	2	1	2	2	2	2	2	2	2	2	2	2	2	2	2	2	2	2
TCGA-78-7536	1	2	2	2	2	2	2	2	2	2	2	1	1	2	2	2	1	2	2	1	2	2	2	2	2	2	2	1	2
TCGA-78-7537	2	2	2	2	2	2	2	2	2	1	2	2	2	2	1	2	2	2	2	2	2	2	2	2	2	2	2	2	2
TCGA-78-7539	2	2	2	2	2	2	1	2	2	2	2	2	2	2	2	1	2	2	2	2	2	2	2	1	2	2	2	2	2
TCGA-78-7540	2	2	2	2	2	2	2	2	1	1	2	2	2	1	2	2	2	2	2	2	2	2	2	2	2	2	2	2	2
TCGA-78-7542	2	2	2	2	1	2	2	2	1	2	2	2	1	2	2	2	2	2	2	2	2	2	2	2	2	2	2	2	2
TCGA-78-7633	2	2	2	2	2	2	2	2	2	2	2	2	2	2	2	2	2	2	2	1	2	2	2	2	2	2	2	2	2
TCGA-78-8640	2	2	2	1	2	2	2	2	2	2	2	2	2	1	2	2	2	2	1	2	2	2	2	2	2	1	2	2	2
TCGA-78-8648	2	2	2	2	2	2	2	2	2	2	2	2	2	2	2	2	2	2	2	2	2	2	2	2	2	2	2	2	2
TCGA-78-8655	2	2	2	1	2	2	2	2	2	2	1	2	2	2	2	2	2	2	2	2	2	2	2	2	2	2	2	2	2
TCGA-78-8660	2	2	2	2	2	2	2	2	2	2	2	2	2	1	2	2	2	2	2	2	2	2	2	2	1	2	2	2	2
TCGA-78-8662	2	2	1	2	1	2	2	1	2	2	2	2	2	2	2	2	2	1	1	2	2	2	1	2	2	2	2	2	1
TCGA-80-5607	2	2	2	2	2	2	2	2	2	2	2	2	2	2	2	1	2	2	2	2	1	2	2	1	2	2	2	2	2
TCGA-80-5608	2	2	1	2	2	2	2	2	2	2	2	2	2	2	2	1	2	2	1	2	2	2	2	2	2	2	2	2	2
TCGA-80-5611	2	2	2	2	2	2	1	2	2	2	2	2	2	1	2	2	2	2	1	2	2	2	2	2	2	2	2	2	1
TCGA-83-5908	2	2	2	2	2	2	2	2	2	2	2	2	2	2	2	2	2	2	1	2	2	2	2	2	2	2	2	2	2
TCGA-86-6562	2	2	2	2	2	2	1	2	2	2	2	2	2	2	2	2	2	2	2	2	2	2	2	2	2	2	2	2	2
TCGA-86-6851	2	2	2	2	2	2	2	2	1	1	2	2	2	2	2	2	2	2	1	2	2	2	2	2	2	2	1	2	2
TCGA-86-7701	1	2	2	2	2	2	2	2	2	2	2	2	2	2	2	2	2	2	1	2	2	2	2	2	2	2	2	1	2
TCGA-86-7711	2	2	2	2	2	2	2	2	1	2	2	2	2	1	2	2	2	2	2	2	2	2	2	2	2	2	2	2	2
TCGA-86-7713	2	2	2	2	2	2	2	2	2	2	2	2	2	2	2	2	2	2	2	2	2	2	2	2	2	2	2	2	2
TCGA-86-7714	2	2	2	2	2	2	2	2	2	2	2	2	2	2	2	2	2	2	2	2	1	2	2	2	2	2	2	2	2
TCGA-86-7953	2	2	2	2	2	2	2	2	2	2	2	2	2	2	2	2	2	2	2	2	2	2	2	2	2	2	2	2	2
TCGA-86-7954	2	1	2	2	2	2	2	2	2	2	2	2	2	2	1	2	2	2	2	2	2	2	2	2	2	2	2	2	2
TCGA-86-7955	2	2	2	2	2	2	2	2	2	2	2	2	2	1	2	2	2	2	2	1	2	2	2	2	2	2	2	2	2
TCGA-86-8054	2	2	2	2	2	2	1	2	2	1	2	2	2	2	2	1	2	2	2	2	2	2	2	2	2	2	2	2	2
TCGA-86-8055	2	2	2	2	2	2	2	2	2	2	2	2	2	2	2	2	2	2	2	2	2	2	2	2	2	2	2	2	2
TCGA-86-8056	2	2	2	2	2	1	1	2	2	2	2	1	1	2	2	2	2	2	2	2	2	2	2	2	2	2	2	2	2
TCGA-86-8073	2	2	2	2	2	2	2	2	2	2	2	2	1	2	2	2	2	2	2	2	2	2	2	2	2	2	2	2	2
TCGA-86-8074	2	2	2	2	1	2	2	2	2	2	2	1	2	2	2	2	2	2	2	2	2	2	2	2	2	2	2	2	2
TCGA-86-8075	2	2	2	2	2	2	2	2	2	2	2	2	2	2	2	2	2	2	2	2	2	2	2	2	2	2	2	2	2
TCGA-86-8076	2	2	2	2	2	2	2	2	2	2	2	2	2	2	2	2	2	2	2	2	2	2	2	2	2	2	2	2	2
TCGA-86-8278	2	2	2	2	2	2	2	2	2	2	2	2	2	2	2	2	1	2	2	2	1	2	2	2	2	2	2	2	2
TCGA-86-8279	2	2	2	2	1	2	2	2	2	2	2	2	1	2	2	2	2	2	1	2	2	2	2	2	2	1	2	2	2
TCGA-86-8280	2	2	2	2	2	2	2	2	2	2	2	2	2	2	2	2	2	2	2	2	2	2	2	2	2	2	2	2	2
TCGA-86-8281	2	2	2	2	2	2	2	2	2	2	2	2	2	1	2	2	2	2	2	2	2	2	2	2	2	2	2	2	1
TCGA-86-8358	1	2	2	2	1	2	2	2	2	2	2	1	2	2	2	2	2	2	2	2	2	2	2	2	2	1	2	2	2
TCGA-86-8359	2	2	2	2	2	2	2	2	2	1	2	2	2	2	2	2	2	2	1	2	2	2	2	2	2	2	2	2	2
TCGA-86-8585	2	2	2	2	2	2	2	2	2	2	1	2	2	1	2	2	2	2	2	2	2	2	2	1	2	1	2	2	2
TCGA-86-8668	2	2	2	2	2	1	2	2	2	2	2	2	2	2	2	2	2	2	2	2	2	2	2	2	2	2	2	2	2
TCGA-86-8669	2	2	2	2	2	2	2	1	2	1	2	2	1	2	2	2	2	2	2	2	2	2	2	2	2	2	2	2	2
TCGA-86-8671	2	2	2	2	2	2	2	2	2	2	2	2	2	2	2	2	2	2	2	2	2	2	2	2	2	2	2	2	2
TCGA-86-8672	2	2	2	2	2	2	2	2	2	2	2	2	2	2	2	2	2	2	1	2	2	2	2	2	2	2	2	2	2
TCGA-86-8673	2	2	2	2	2	2	2	2	2	2	2	2	2	2	2	2	2	2	1	2	1	2	2	2	2	2	2	2	2
TCGA-86-8674	2	2	2	2	2	2	2	2	1	2	2	2	2	1	2	2	2	2	2	2	2	2	2	2	2	2	2	2	2
TCGA-86-A456	2	2	2	2	2	2	2	2	2	2	2	2	2	2	2	2	2	2	1	2	2	2	2	2	2	2	2	2	2
TCGA-86-A4D0	NA	NA	NA	NA	NA	NA	NA	NA	NA	NA	NA	NA	NA	NA	NA	NA	NA	NA	NA	NA	NA	NA	NA	NA	NA	NA	NA	NA	NA
TCGA-86-A4JF	2	2	2	2	2	2	2	2	2	2	2	2	2	2	2	1	2	2	2	2	2	1	1	2	2	1	2	2	2
TCGA-86-A4P7	2	2	2	2	2	2	2	2	2	2	2	2	2	2	2	2	2	2	2	2	2	2	2	2	2	2	2	2	2
TCGA-86-A4P8	2	2	2	2	2	2	2	2	2	2	2	2	2	2	2	2	2	2	2	2	2	2	2	2	2	2	2	2	2
TCGA-91-6828	2	2	2	2	2	2	2	2	1	1	2	2	2	2	1	2	2	2	1	2	2	2	2	2	2	2	2	1	2
TCGA-91-6829	2	2	2	1	2	1	2	1	1	2	2	1	1	2	2	2	2	2	2	1	2	2	2	2	2	2	2	2	2
TCGA-91-6830	2	2	2	2	2	2	2	2	2	2	2	2	2	2	2	2	2	2	1	2	2	2	2	2	2	1	2	2	2
TCGA-91-6831	2	2	2	2	2	2	2	2	2	2	2	2	2	2	2	2	1	2	2	2	2	2	2	2	2	2	2	2	2
TCGA-91-6835	2	2	2	2	2	2	1	2	2	2	2	2	2	2	2	1	2	2	2	2	2	2	2	2	2	2	2	2	1
TCGA-91-6836	2	2	1	2	2	2	1	2	2	2	2	2	2	2	2	2	2	2	1	2	1	1	2	2	1	2	2	2	2
TCGA-91-6840	2	2	2	2	2	2	2	2	2	2	2	2	2	2	2	2	2	1	2	2	2	2	2	2	2	2	2	2	2
TCGA-91-6847	2	2	2	2	2	2	2	2	2	2	2	1	2	2	2	2	2	2	2	2	2	2	2	2	2	2	2	2	2
TCGA-91-6848	2	2	2	2	1	2	1	2	2	2	2	2	2	2	1	2	1	2	1	2	1	2	2	2	2	2	2	2	1
TCGA-91-6849	2	2	1	2	2	2	2	2	2	2	2	2	2	2	2	2	2	2	2	2	2	2	2	2	2	2	2	2	2
TCGA-91-7771	2	2	2	2	2	2	2	2	2	2	2	2	2	2	2	2	1	2	2	2	2	2	2	2	2	2	2	2	2
TCGA-91-8496	2	2	2	2	2	2	2	2	2	2	2	2	2	2	2	2	2	2	2	2	2	2	2	2	2	2	2	2	2
TCGA-91-8497	2	2	2	2	2	2	2	2	2	2	2	2	2	2	2	2	2	2	2	2	2	2	2	2	2	2	2	2	2
TCGA-91-8499	2	2	2	2	2	2	2	2	2	2	2	2	2	2	2	2	2	2	2	2	2	2	2	2	1	2	2	2	2
TCGA-91-A4BC	2	2	2	2	2	2	2	2	2	2	2	2	2	2	2	2	2	2	1	2	2	2	2	2	2	2	2	2	2
TCGA-91-A4BD	2	2	2	2	2	2	2	2	2	2	2	2	2	2	2	2	2	2	2	1	2	2	2	2	2	2	2	2	2
TCGA-93-7347	2	2	2	2	2	2	2	2	2	2	2	2	2	2	2	2	2	2	1	2	2	2	2	2	2	2	2	2	2
TCGA-93-7348	2	2	2	2	2	2	2	2	2	2	2	2	2	2	2	2	2	2	2	2	2	2	2	2	2	2	2	2	2
TCGA-93-8067	2	2	1	2	2	2	2	2	2	2	2	2	2	2	2	2	2	2	1	2	2	2	1	2	2	2	1	2	2
TCGA-93-A4JN	2	2	2	2	2	2	2	2	2	2	2	1	2	2	2	1	2	2	2	2	2	2	2	2	2	2	2	2	2
TCGA-93-A4JO	2	2	2	2	2	2	2	2	2	2	2	2	2	2	2	2	2	2	2	2	2	2	2	2	2	2	2	2	2
TCGA-93-A4JP	2	2	2	2	2	2	2	2	2	2	2	1	2	2	2	2	2	2	2	2	2	2	2	2	2	2	2	2	2
TCGA-93-A4JQ	2	2	2	2	2	2	2	2	2	2	2	1	2	2	2	2	2	2	2	1	2	2	2	2	2	2	2	2	2
TCGA-95-7039	2	2	2	2	1	2	2	2	2	2	2	2	2	2	2	2	2	1	1	1	2	2	2	2	2	2	1	2	2
TCGA-95-7043	2	2	2	2	2	2	2	2	1	2	2	2	1	2	2	2	2	2	2	1	2	2	2	2	1	2	2	2	2
TCGA-95-7562	1	2	1	2	1	2	2	2	2	2	2	1	2	2	2	2	2	2	1	2	2	2	2	2	2	2	2	1	2
TCGA-95-7567	2	2	1	2	2	2	2	2	2	1	2	2	2	2	2	1	2	2	1	2	2	2	2	2	1	1	2	2	1
TCGA-95-7944	2	2	2	2	2	2	2	2	2	2	2	2	1	2	2	2	2	2	2	2	2	2	2	2	2	2	2	2	2
TCGA-95-7947	2	2	2	2	2	2	2	2	2	2	2	1	2	2	2	2	2	2	2	2	2	2	2	2	2	2	2	2	2
TCGA-95-7948	2	2	2	2	2	2	2	2	2	2	2	2	2	2	2	2	2	2	2	2	2	2	2	2	2	2	2	2	2
TCGA-95-8039	2	2	2	2	2	2	2	2	2	2	2	2	2	2	2	2	2	2	2	2	2	2	2	2	2	2	2	2	2
TCGA-95-8494	2	2	2	2	2	2	2	2	2	2	2	2	2	2	2	2	2	2	2	2	2	2	2	2	2	2	2	2	2
TCGA-95-A4VK	2	2	2	2	2	2	2	2	2	2	2	2	2	1	2	2	2	2	1	2	2	2	2	2	2	2	2	2	2
TCGA-95-A4VN	2	2	2	2	2	1	2	2	2	2	2	2	2	2	2	2	2	2	2	2	2	2	1	2	2	1	2	2	2
TCGA-95-A4VP	2	2	2	2	2	2	2	2	2	2	2	2	2	2	2	1	2	2	2	2	2	2	2	2	2	2	2	2	2
TCGA-97-7546	2	2	2	1	2	2	2	2	2	2	2	2	2	2	2	2	2	2	2	2	2	2	2	2	2	2	2	2	2
TCGA-97-7547	2	2	2	2	2	2	2	1	2	2	2	2	1	2	2	2	2	2	2	2	2	2	2	2	2	1	2	2	2
TCGA-97-7552	2	2	2	2	2	2	2	2	2	2	2	2	2	2	2	2	2	2	2	2	2	2	2	2	2	2	2	2	2
TCGA-97-7553	2	2	2	2	2	2	2	2	2	2	2	1	1	2	2	2	2	2	2	2	2	2	2	2	2	2	2	2	2
TCGA-97-7554	2	2	2	2	2	2	2	1	2	2	2	2	2	2	2	2	2	2	1	2	2	2	2	2	2	2	2	2	2
TCGA-97-7937	2	2	2	2	2	2	2	2	2	1	2	2	2	2	2	2	2	2	1	2	2	2	2	2	2	2	2	2	2
TCGA-97-7938	2	2	2	1	2	2	2	2	2	2	2	2	2	2	2	2	1	2	1	2	2	2	2	2	2	2	2	2	2
TCGA-97-7941	2	2	2	2	2	2	2	2	2	2	2	2	2	2	2	2	2	2	2	2	1	2	2	2	2	2	2	2	2
TCGA-97-8171	2	2	2	2	2	2	2	2	2	2	2	2	2	2	2	2	2	2	2	2	2	2	2	2	2	2	2	2	2
TCGA-97-8172	2	2	2	2	2	2	2	2	1	1	2	1	2	2	2	2	2	2	2	2	2	2	2	2	2	2	2	2	1
TCGA-97-8174	2	2	2	2	2	1	2	2	2	2	2	2	2	2	2	2	2	2	2	2	2	2	2	2	2	2	2	2	2
TCGA-97-8175	2	2	2	2	2	2	2	2	2	2	2	2	2	1	2	2	2	2	2	2	2	2	2	2	2	2	2	2	2
TCGA-97-8176	2	2	2	2	2	2	2	2	2	2	2	2	2	1	2	1	2	2	2	2	2	2	2	2	2	2	2	2	2
TCGA-97-8177	2	2	2	2	2	2	2	2	2	2	2	2	2	2	2	2	2	2	2	2	2	2	2	2	2	2	2	2	2
TCGA-97-8179	2	2	2	2	2	2	2	2	2	2	2	2	2	2	2	2	2	2	2	2	2	2	2	2	2	2	2	2	2
TCGA-97-8547	2	2	2	2	2	2	2	2	2	2	2	2	2	2	2	2	2	2	2	2	2	2	2	2	2	2	2	2	2
TCGA-97-8552	2	2	2	2	2	2	2	2	2	2	2	2	2	2	2	2	2	2	2	2	2	2	2	2	2	2	2	2	2
TCGA-97-A4LX	2	2	2	2	1	2	2	2	2	2	2	2	2	2	2	2	2	2	1	1	2	2	2	2	2	2	2	2	2
TCGA-97-A4M0	2	2	2	1	1	2	2	2	2	2	2	2	2	2	2	2	2	2	2	2	2	2	2	2	2	2	2	2	2
TCGA-97-A4M1	2	2	2	2	2	2	2	2	2	2	2	2	2	2	2	2	2	2	2	2	2	2	2	2	2	2	2	2	2
TCGA-97-A4M2	2	2	2	2	2	2	2	2	2	2	2	2	2	2	2	2	2	2	2	2	2	2	2	2	2	2	2	2	2
TCGA-97-A4M3	2	2	2	2	2	2	2	2	2	2	2	2	2	2	2	1	2	2	1	2	2	2	2	2	2	2	2	2	1
TCGA-97-A4M5	2	2	2	2	2	2	2	2	2	2	2	2	2	2	2	2	2	2	2	2	2	2	2	2	2	2	2	2	2
TCGA-97-A4M6	2	1	2	2	2	2	2	2	2	2	2	2	2	2	2	2	2	2	1	2	1	2	2	2	2	2	2	2	2
TCGA-97-A4M7	2	2	2	2	2	2	2	2	2	2	2	2	2	2	1	2	2	2	2	2	1	2	2	2	2	2	2	2	2
TCGA-99-7458	2	2	2	1	2	2	2	2	2	2	2	2	2	2	2	2	2	2	2	2	2	2	2	2	2	2	2	2	2
TCGA-99-8025	2	2	2	1	2	2	2	2	2	2	2	1	2	2	2	2	1	2	1	2	2	2	2	2	2	2	2	2	2
TCGA-99-8028	2	2	1	2	2	2	2	2	2	2	2	2	2	2	2	2	2	2	1	2	2	2	2	2	2	2	2	2	2
TCGA-99-8032	2	2	2	2	2	2	2	2	2	2	2	2	2	2	2	2	2	2	1	2	2	2	2	2	2	2	2	2	2
TCGA-99-8033	2	2	2	2	2	2	2	2	2	2	2	2	2	2	2	2	2	2	2	2	2	2	2	2	2	1	2	2	2
TCGA-J2-8192	2	2	2	1	2	2	2	2	2	2	2	2	2	2	2	2	2	2	2	2	2	2	2	2	2	2	2	2	2
TCGA-J2-8194	2	2	2	2	2	2	2	2	2	2	2	2	2	1	2	2	2	2	1	2	2	2	2	2	2	1	2	2	2
TCGA-J2-A4AD	2	2	2	2	2	2	2	2	2	2	2	1	2	2	2	2	2	2	2	2	2	2	2	2	2	2	2	2	2
TCGA-J2-A4AE	2	2	2	2	2	2	2	2	2	2	2	2	2	2	2	2	2	2	1	2	2	2	1	2	2	1	2	2	2
TCGA-J2-A4AG	2	2	2	2	1	2	1	2	2	2	2	1	1	2	2	2	1	2	1	2	2	2	1	2	2	2	2	2	2
TCGA-L4-A4E5	2	2	2	2	2	2	2	2	2	2	2	2	2	2	2	2	2	2	2	2	2	2	2	2	2	2	2	2	2
TCGA-L4-A4E6	[illegible]	[illegible]	[illegible]	[illegible]	[illegible]	[illegible]	[illegible]	[illegible]	[illegible]	[illegible]	[illegible]	[illegible]	[illegible]	[illegible]	[illegible]	[illegible]	[illegible]	[illegible]	[illegible]	[illegible]	[illegible]	[illegible]	[illegible]	[illegible]	[illegible]	[illegible]	[illegible]	[illegible]	[illegible]
TCGA-L9-A443	2	2	2	2	2	2	2	2	2	2	2	2	2	2	2	1	2	2	1	2	2	2	2	2	2	2	2	2	2
TCGA-L9-A444	2	2	2	2	1	2	1	2	2	2	2	2	1	2	2	2	2	2	1	1	2	2	2	2	2	2	2	2	2
TCGA-MN-A4N1	2	2	2	1	2	2	2	2	2	2	2	2	2	2	2	2	2	2	1	1	2	2	2	2	2	2	1	2	2
TCGA-MN-A4N4	2	2	2	2	2	2	2	1	2	1	2	2	2	2	2	1	1	2	1	2	2	2	2	2	2	1	2	2	2
TCGA-MN-A4N5	2	1	2	2	2	2	2	2	2	2	1	2	2	2	2	2	2	2	2	2	2	2	2	2	2	2	2	2	2
TCGA-MP-A4SV	2	2	1	1	2	2	1	2	2	2	2	2	2	2	1	2	2	2	2	2	2	2	1	2	2	2	2	2	2

TCGA-MP-A4SW	1	1	RAS WT	1	5	2	2	1	2	2	2	2	2	2	2	2	2	2	2	2	2	2	2	2	2	2	2	2	2	2	2	2	2	2	2	2	2	2	1	2	2	2	2	2	2	2	2	2	2	2	2	2	2
TCGA-MP-A4SY	3	2	RAS WT	2	4	2	2	2	2	2	2	2	2	2	2	2	2	2	1	2	2	2	2	2	2	2	2	2	2	2	2	2	2	2	2	2	1	1	2	2	2	2	2	1	1	2	2	2	2	2	1	2	2
TCGA-MP-A4T4	3	2	K-TP53	2	3	1	2	3	2	2	1	2	2	2	2	2	2	2	2	2	2	2	2	2	2	2	2	2	2	2	2	1	2	2	1	2	2	2	2	2	2	2	1	2	2	2	2	2	2	2	2	2	2
TCGA-MP-A4T6	1	1	RAS WT	1	4	2	2	1	2	2	2	2	2	2	2	2	2	2	2	2	2	2	2	2	2	2	2	2	2	2	2	2	2	2	2	2	2	2	2	2	2	2	2	2	2	2	2	2	1	2	2	2	2
TCGA-MP-A4T7	2	2	K-Ox	2	3	2	2	1	1	2	1	2	2	2	2	2	2	2	2	2	1	2	2	2	2	2	2	2	2	2	1	2	2	2	2	2	2	2	2	2	2	2	2	2	2	2	2	2	2	2	2	2	2
TCGA-MP-A4T8	2	2	K-Ox	2	4	2	2	2	2	2	1	2	2	2	2	2	2	2	2	2	2	2	2	2	2	2	2	2	2	2	2	2	2	2	2	2	2	2	2	2	2	2	1	2	2	2	2	2	2	2	2	2	2
TCGA-MP-A4T9	1	1	RAS WT	1	5	1	2	2	2	2	2	2	2	2	2	2	2	2	2	2	2	2	2	2	2	2	2	2	2	2	2	2	2	2	2	2	2	2	2	2	2	2	2	2	2	2	2	2	2	2	2	2	2
TCGA-MP-A4TA	2	2	RAS WT	2	4	1	2	2	2	2	2	2	2	2	2	2	2	2	2	2	2	2	2	2	2	2	2	2	2	2	2	1	2	2	2	2	2	1	2	2	2	2	2	2	2	2	2	2	2	2	2	2	2
TCGA-MP-A4TC	3	2	RAS WT	2	4	1	2	4	2	2	2	2	2	2	2	2	2	2	2	2	2	2	2	2	2	2	2	2	2	2	2	2	2	2	2	2	2	2	2	2	1	2	2	2	2	2	2	2	2	2	2	2	2
TCGA-MP-A4TD	2	2	K-Ox	2	3	2	1	2	2	1	1	2	2	2	2	2	2	2	2	2	2	2	2	2	2	2	2	2	2	2	2	2	2	2	2	2	2	2	2	2	2	2	1	2	2	2	2	2	2	2	2	2	2
TCGA-MP-A4TF	2	2	K-Ox	2	5	1	2	1	2	1	1	2	2	2	2	2	2	2	2	2	2	2	2	2	2	2	1	2	1	2	2	2	1	2	2	2	2	2	2	2	2	2	1	2	2	2	2	2	2	2	1	2	2
TCGA-MP-A4TH	1	2	K-Ox	2	3	2	2	3	2	1	1	2	2	2	2	2	2	2	2	2	2	2	2	2	2	2	2	2	2	2	2	2	2	2	2	2	2	2	2	2	2	2	1	2	2	2	1	2	2	2	1	2	2
TCGA-MP-A4TI	3	2	K-TP53	2	3	1	2	3	1	2	1	2	2	2	2	2	2	2	1	2	2	2	2	2	2	1	2	2	2	2	2	2	2	2	2	2	2	2	2	1	1	2	1	2	2	2	2	2	2	2	2	2	2
TCGA-MP-A4TJ	1	2	K-Ox	2	3	2	2	4	2	2	1	2	2	2	2	2	2	2	1	2	2	2	2	2	2	2	2	2	2	2	2	2	2	2	2	2	2	2	2	1	2	2	2	2	2	2	2	2	2	2	2	2	2
TCGA-MP-A4TK	3	2	K-TP53	2	4	1	2	2	2	2	1	2	2	2	2	2	2	2	2	2	2	2	2	2	2	1	2	2	2	2	2	2	2	2	2	2	2	2	2	2	2	2	2	2	2	2	2	2	1	2	2	2	2
TCGA-MP-A5C7	2	2	RAS WT	2	3	2	2	2	1	2	2	2	2	2	2	2	2	2	2	2	2	1	2	2	2	2	2	2	2	2	2	2	2	2	2	2	2	2	2	2	2	1	2	2	2	2	2	2	2	2	2	2	2
TCGA-NJ-A4YF	2	2	RAS WT	2	4	1	2	3	2	1	2	2	2	2	2	2	2	2	2	2	2	2	2	2	2	2	2	2	2	2	2	2	2	2	2	2	2	2	2	2	2	2	1	2	2	2	2	2	2	2	2	2	2
TCGA-NJ-A4YI	1	1	K-EGFR	2	3	2	1	2	2	2	1	2	2	2	2	2	2	2	2	2	2	2	2	2	2	2	2	2	2	2	2	2	2	2	2	2	2	2	2	2	2	2	1	2	2	2	2	2	2	2	2	2	2
TCGA-NJ-A4YP	2	2	K-Ox	2	3	2	2	3	2	1	1	2	2	2	2	2	2	2	2	2	2	1	2	2	2	2	2	2	2	2	2	2	2	2	2	2	2	2	2	1	2	2	2	2	2	2	1	2	2	2	2	2	2
TCGA-NJ-A4YQ	3	2	RAS WT	2	3	2	2	2	1	2	2	2	2	2	2	2	2	2	2	2	2	2	2	2	2	2	1	1	1	2	2	2	2	2	2	2	2	1	2	2	2	2	1	2	2	2	2	2	1	2	2	2	2
TCGA-NJ-A55A	1	2	RAS WT	2	3	2	2	3	2	2	2	2	2	2	2	2	2	2	2	2	2	2	2	2	2	2	2	2	2	2	2	2	2	2	2	2	1	2	2	2	2	2	1	2	2	2	2	2	2	2	2	2	2
TCGA-NJ-A55O	1	2	K-Ox	2	3	2	2	3	2	1	1	2	2	2	2	2	2	2	2	2	2	2	2	2	2	2	2	2	2	2	2	2	1	2	2	2	2	2	2	2	2	2	2	2	2	2	2	2	2	2	2	2	2
TCGA-NJ-A55R	2	2	K-Ox	2	3	2	1	3	1	1	1	2	2	2	2	2	2	2	2	2	2	2	2	2	2	2	1	1	2	2	2	2	2	2	2	2	2	2	2	2	2	2	1	2	2	2	2	2	2	2	2	2	2
TCGA-O1-A52J	1	1	RAS WT	1	4	1	2	2	2	2	2	2	2	2	2	2	2	2	2	2	2	2	2	2	2	1	2	2	2	2	2	2	2	2	2	2	2	2	2	2	2	2	1	2	2	2	2	2	2	2	2	2	2
